# Evaluating the implementation and impact of the HEart faiLure carer support Programme (HELP) in the United Kingdom: A study protocol for a multi-centre, mixed-method, implementation study

**DOI:** 10.1371/journal.pone.0347037

**Published:** 2026-04-17

**Authors:** Gareth Thompson, Judy Bradley, Martin Dempster, Patrick Stark, Mike Clarke, Nicola Johnston, Lana Dixon, Patricia Campbell, Patrick Donnelly, Theresa McDonagh, Susan Piper, Yvonne Millerick, Loreena Hill, Donna Fitzsimons

**Affiliations:** 1 School of Nursing and Midwifery, Queen’s University Belfast, Belfast, United Kingdom; 2 School of Medicine, Dentistry, and Biomedical Sciences, Queen’s University Belfast, Belfast, United Kingdom; 3 School of Psychology, Queen’s University Belfast, Belfast, United Kingdom; 4 Royal Victoria Hospital, Belfast Health and Social Care Trust, Belfast, United Kingdom; 5 Craigavon Area Hospital, Southern Health and Social Care Trust, Portadown, United Kingdom; 6 Ulster Hospital, South Eastern Health and Social Care Trust, Dundonald, United Kingdom; 7 King’s College Hospital, NHS Foundation Trust, London, United Kingdom; 8 Department of Nursing, Community and Public Health, Glasgow Caledonian University, Glasgow, United Kingdom; 9 Glasgow Royal Infirmary, NHS Greater Glasgow and Clyde, Glasgow, United Kingdom; 10 School of Nursing and Paramedic Science, Ulster University, Londonderry, United Kingdom; PLOS: Public Library of Science, UNITED STATES OF AMERICA

## Abstract

**Background:**

Informal carers (*i.e.,* family members or friends) of patients with heart failure are ill-prepared and under-supported for their caregiving role. To address this issue, the HEart faiLure carer support Programme was co-designed with carers and healthcare professionals, with pilot testing demonstrating intervention feasibility and acceptability. In the current article, we present the study protocol for evaluating the implementation and impact of the HEart faiLure carer support Programme in real-world, clinical settings across the United Kingdom (ClinicalTrials.gov ID: NCT07373041).

**Methods:**

A mixed-method, implementation study adopting a multi-centre, prospective cohort study design, with nested process and economic evaluations will be conducted. Nurses will deliver the HEart faiLure carer support Programme to 180 carers of patients with symptomatic heart failure across five sites spanning three nations of the United Kingdom (Northern Ireland, England, and Scotland). The patients (approximately 180) of carers enrolled in the project will be invited to provide outcome data. Recruited carers will receive weekly, nurse-led, online support group sessions and supplementary, self-directed educational resources (a booklet and website) for six weeks. Quantitative (*i.e.,* questionnaires and logs) and qualitative (*i.e.,* interviews) data will be collected from carers, patients, and healthcare professionals throughout the study. These data will evaluate the acceptability, fidelity, context, economic cost, and impact of HEart faiLure carer support Programme delivery in real-world clinical settings. An integrative analysis with mapping to the Consolidated Framework for Implementation Research and Normalisation Process Theory domains will be conducted, which will identify key synergies across quantitative and qualitative data sets.

**Discussion:**

The results will elucidate the factors underpinning successful intervention translation to clinical practice and identify any required contextual adaptations, along with generating preliminary evidence of intervention impact. These findings will inform a large-scale, type 2 hybrid study, advancing the HEart faiLure carer support Programme towards routine rollout across the United Kingdom.

## 1.0 Introduction

The global prevalence of heart failure (HF) is approximately 55.5 million, with this condition affecting more than one million individuals in the United Kingdom (UK) [1]. Effective self-management by these patients constitutes a key strategy for reducing hospital readmissions and improving quality of life (QoL) [2]. As HF progresses, self-management of symptom burden becomes increasingly challenging, often resulting in patients relying on support from informal carers (*i.e.,* family members or friends) [2]. However, previous research has comprehensively reported that these carers experience diminished mental and physical health and QoL, which correlates with the physical and emotional challenges of their caring role and the health status of the patient they care for [3,4].

The vital role fulfilled by carers in supporting patients with self-management is reflected in the European Society of Cardiology guidelines for HF treatment [2]. However, given the challenges faced, these carers require improved education and emotional support for sufficient empowerment in their caregiving role [3–7]. A recent systematic review and meta-analysis demonstrated a scarcity of interventions supporting carers of patients with HF, with the few studies available showing insignificant results and poor reporting of intervention development [8]. Moreover, there were no supportive interventions co-designed with carers in the UK to target their specific psychoeducational needs and requirements [8].

To address this significant area of unmet need, our research team systematically co-developed the ‘HEart faiLure carer support Programme (HELP)’ according to the UK Medical Research Council (MRC) framework for developing and evaluating complex interventions [9]. The intervention development process is comprehensively reported elsewhere [10]. In summary, previous evidence and theory [3,4,8] underpinned an interdisciplinary stakeholder group (carers, healthcare professionals, and academics) who co-developed and refined the design and content of HELP using an inclusive, sequential process [10]. This resulted in the production of a nurse-led, psychoeducational intervention comprising weekly online support group sessions and self-directed educational resources (booklet and website) delivered over a six-week period.

Following HELP development, a convergent, mixed-method, pilot study was conducted in the UK [10]. This research demonstrated that HELP is feasible and acceptable for carers of patients with HF, with participants highly rating the usefulness of the intervention. Moreover, there was preliminary evidence of potential improvements in carer preparedness and knowledge; emotional wellbeing; and empowerment in their care giving role [10]. However, there is uncertainty whether the findings of this pilot study can be replicated in the real-world environment, and the key factors underpinning successful integration of HELP into clinical environments are unclear. In this article, we report the study protocol for evaluating the implementation and impact of HELP in clinical practice, which will inform advancements of HELP towards routine rollout across the UK.

### 1.1 Aim

The overarching aim is evaluating the implementation and impact of HELP in real-world clinical settings across the UK.

### 1.2 Objectives

1) Evaluate the multi-faceted implementation strategy via a process evaluation of acceptability, fidelity, and context of HELP delivery across real-world clinical sites.
2) Estimate the economic cost of HELP implementation.
3) Explore the short and long-term impact of HELP on outcomes for carers and patients.

## 2.0 Methods

**Fig 1:**
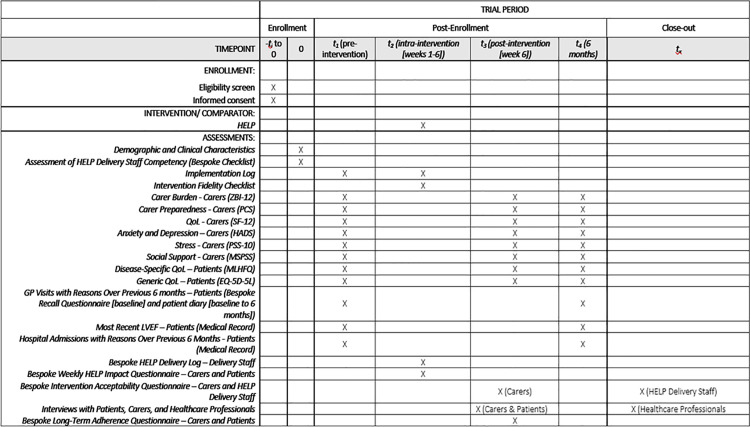
SPIRIT 2025 schedule of enrolment, interventions, and assessments. **Legend:** HELP = HEart faiLure carer support Programme; ZBI-12 = Zarit Caregiver Burden Interview Short Form; PCS = Preparedness for Caregiving Scale; QoL = Quality of Life; SF-12 = 12-Item Short Form Survey; HADS = Hospital Anxiety and Depression Scale; PSS-10 = Perceived Stress Scale; MSPSS = Multidimensional Scale of Perceived Social Support; MLHFQ = Minnesota Living with Heart Failure Questionnaire; EQ-5D-5L = EuroQol Five Dimension Questionnaire; GP = General Practitioner; and LVEF = Left Ventricular Ejection Fraction.

This study protocol is reported in accordance with the Standard Protocol Items: Recommendations for Interventional Trials (SPIRIT) guidelines (Fig 1 and S1 Checklist) [11] and the Standards for Reporting Implementation Studies (StaRI) (S2 Checklist) [12]. This study was registered on ClinicalTrials.gov (ID: NCT07373041). Queen’s University Belfast will lead all aspects of the study and serve as the sponsor.

### 2.1 Patient and public involvement

A Steering Group was established to oversee the design, conduct, and reporting of the trial. This group comprises academics, carers, patients with HF, healthcare professionals, and representatives from patient and carer support organisations. During project delivery, HELP champions (carers who received the intervention) will be recruited across the collaborating sites to the Steering Group to provide input on their experience and any required intervention adaptations. A range of protected characteristics (*i.e.,* age, socio-economic status, and ethnicity) are and will be present across the members to ensure equality and diversity. The Steering Group will meet monthly and virtually over the project lifetime to provide critical assessment, monitoring, and consensus agreement on project delivery (*i.e.,* context-specific tailoring), issue resolution (*i.e.,* recruitment and retention), and dissemination activities. Communication between meetings will occur as required.

### 2.2 Design

A mixed-method, implementation study adopting a multi-centre prospective cohort study design, with nested process and economic evaluations will be conducted. The implementation strategy assessments will be underpinned by the integration of the Consolidated Framework for Implementation Research (CFIR) [13] and Normalisation Process Theory (NPT) [14], which will provide holistic insights into the macro (*i.e.,* system-level) and micro (*i.e.,* individual-level) factors influencing implementation outcomes [15].

#### 2.2.1 Setting.

This study will be conducted across five hospital sites spanning three regions of the UK (Northern Ireland, England, and Scotland). Local HF nurses will deliver HELP to carers of patients with HF within their corresponding region. The collaborating sites will constitute early adopters of HELP and will be designated as ‘Beacon Sites’. These Beacon Sites will be used to model programme implementation, which is a method of informing and supporting embedding of innovative practice [16]. The collaborating sites were selected due to their ability to commit to HELP implementation, to diversify sample inclusion, and increase geographic spread.

#### 2.2.2 Sample and recruitment.

This study will recruit participants across three populations: 1. Carers, 2. Patients with HF, and 3. Healthcare Professionals. In compliance with the Screened, Eligible, Approached, Randomised/ Recruited (SEAR) framework [17], anonymised screening and recruitment logs will be used to document numbers and percentages of eligible and recruited participants, along with reasons for non-participation and withdrawal. These data will be monitored by the Steering Group to identify recruitment barriers and inform necessary strategies to optimise enrolment across the collaborating sites.

#### 2.2.4 Carers.

The target population of HELP is informal carers of patients with symptomatic HF. These individuals will be eligible to participate if they are: 1) Aged 18 years or older; 2) Physically and mentally capable of operating a digital device; 3) Able to engage comfortably in conversation with peers and capable of reading written materials; 4) Caring (regular, unpaid care) for a person with a clinical diagnosis of HF who is receiving standard evidence-based HF therapies and has either HF symptoms or one episode of decompensation (symptoms requiring medical attention) in previous six months; and 5) Capable of speaking and understanding English.

There will be five programme rollouts of HELP (six weeks each) across each of the three collaborating nations over a one-year period, with twelve carers per rollout (60 carers per nation; 180 carers in total). This sample size was pragmatically determined by the maximum study duration within the funding limit, along with the recruitment rate demonstrated in the pilot study [10].

#### 2.2.5 Patients.

Patients who are receiving informal support from the recruited carers will be invited to participate via the provision of data, which will allow their perspectives on HELP implementation to be captured, whilst also facilitating an assessment of the potential, dyadic impact of the intervention. These patients will be eligible to participate if they are: 1) Aged 18 years or older; 2) Physically and mentally able to engage comfortably in conversation and capable of reading written materials; 3) Clinically diagnosed with HF and receiving standard evidence-based HF therapies and either have HF symptoms or one episode of decompensation (symptoms requiring medical attention) in previous six months; 4) Able to speak and understand English; and 5) Receiving regular, unpaid support with managing their condition from a loved one (*i.e.,* a relative or spouse) or friend. Patients will be ineligible if their carers refused to participate in HELP. Carers will remain eligible for participation regardless of patient enrolment. One patient per carer will be invited to participate to enhance sample diversity (approximately 180 patients in total).

#### 2.2.6 Carer and patient recruitment.

Local cardiology teams will identify and approach potential participants during routine, in and out-patient HF appointments. Eligible patients will receive information packs for both them and their carer. Those interested in participation will provide permission to be contacted by the research team for recruitment. During this contact, patients will also signpost the research team to their interested carers. Subsequently, these carers will be contacted by the research team for eligibility screening and recruitment. The collaborating hospital sites cover regions with a range of socio-demographic characteristics (*i.e.,* education level, socio-economic status, and ethnicity), which will maximise the diversity of the recruited sample.

#### 2.2.7 Healthcare professionals.

Healthcare professionals (*i.e.,* HELP delivery staff, cardiologists, and HF nurses) across the collaborating sites will be invited to provide process evaluation data. These individuals will be supplied with information sheets and will be asked to contact the research team if interested in participation. Healthcare professionals will be eligible if they are 1) Members of the cardiology team, 2) Aged 18 years or older, and 3) Willing to provide informed consent.

#### 2.2.8 Intervention design.

HELP design was based on prior literature and comprehensive input from carers of patients with HF and healthcare professionals, with the co-development and feasibility testing of this intervention previously reported in detail [10]. HELP constitutes a six-week, nurse-led, psychoeducational programme comprising two components:

1) *Online support group sessions*

Nurse-led, weekly online support group sessions are delivered to groups of approximately twelve carers over a six-week period. Each session lasts sixty minutes and is underpinned by a standardised, educational presentation covering the topics displayed in Table 1. Following each educational presentation, the HF nurse facilitates a group discussion to issue tailored advice, guidance, and signposting, along with providing a platform for carers to engage in peer support.

**Table 1:** Support Group Schedule.

Session Number	Topic	Content
1	Introduction	Group introductions Summary of HELP content Guidance on access and usage of self-directed educational resources (booklet and website)
2	Understanding HF	Overview of HF Symptoms Prognosis Palliative care
3	Personal Wellbeing	Looking after physical and emotional wellbeing Management strategies for complex emotions
4	Communication and Support	Communicating with loved ones and healthcare professionals
5	Practical Skills for Supporting Self-Management	Controlling diet and salt intake Management of fluid and medication Exercise Monitoring symptoms at home
6	Feedback	Discussion of experience and final comments/ questions Feedback on HELP and areas of improvement

**Legend** HELP = HEart faiLure carer support Programme and HF = heart failure.

2) *Self-directed, educational resources (booklet and website)*

Upon HELP enrolment, carers will receive an educational booklet (S3 Text) and access to a supplementary website. Both resources were co-designed and professionally produced. The educational booklet complements the online support group sessions by facilitating self-directed learning across the following five chapters:

Chapter 1: What is HF?
Chapter 2: Planning for the future
Chapter 3: Looking after yourself
Chapter 4: Communication
Chapter 5: Your role as a carer

Each chapter is completed over the week leading to the corresponding online support group session. The self-directed website supplements the booklet by presenting the same educational information in a potentially more engaging format (*i.e.,* animations, interactive content, and topic summaries). Additionally, the website provides access to the standardised educational presentations for the online support group sessions, along with facilitating the submission of non-urgent questions for the HF nurse to answer at the subsequent online support group session.

**Fig 2:**
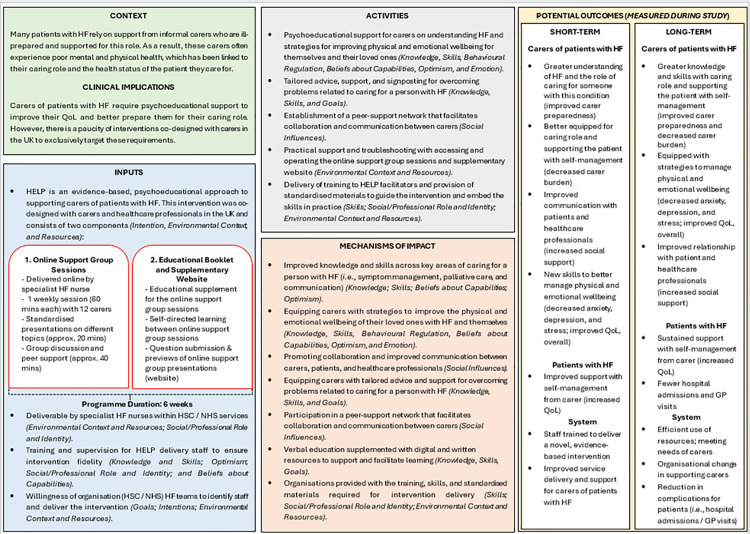
HELP Logic Model. **Legend** HF = Heart Failure; QoL = Quality of Life; UK = United Kingdom; HELP = HEart faiLure carer support Programme; HSC = Health and Social Care; NHS = National Health Service; and in parentheses () and italics = mapping to Theoretical Domains Framework.

HELP is underpinned by the Transactional Theory of Stress and Coping [18], which postulates that adaptive coping strategies can moderate the relationship between the perception of potentially stressful experiences and negative outcomes (*i.e.,* burnout and distress). By facilitating improved access to information and support from peers and healthcare professionals, the intervention will deliver key practical and emotional coping resources to alleviate stress, burden, and improve carer wellbeing, ultimately empowering carers in their caregiving role. The intervention components are mapped to the Theoretical Domains Framework, which facilitates further theoretical grounding of the mechanisms of impact for behavior change [19]. Consistent with MRC guidance for complex interventions [20], Fig 2 presents a preliminary logic model illustrating the key aspects of HELP, which will evolve according to the results of this study.

#### 2.2.9 Intervention delivery.

HELP will be delivered by local specialist HF nurses across the collaborating sites. Following recruitment, carers will be assigned a start date for HELP. One week prior to the first online support group session, carers will receive the educational booklet and access to the supplementary website, along with supporting programme materials (*i.e.,* online support group schedule and guidance on joining online sessions). Carers will be advised to read each chapter of the educational booklet and supplementary website before the corresponding online support group session. The online support group sessions will run on ‘Zoom’, with a link and password to each session issued to carers before commencement. Carers will receive a reminder email two days before each online support group session. The research team will remain in contact with all participants to provide ongoing support with accessing the online support group sessions and supplementary website.

The nurse-led, online support group sessions will be delivered using an informal approach to facilitate a comfortable environment for the carers. Each session will consist of twelve carers and last approximately sixty minutes. The sessions will initiate with a standardised, educational presentation (approximately 20 minutes) on the allocated topic, with the remainder of the session (approximately 40 minutes) dedicated to questions raised during the session or submitted via the website; group discussion; tailored advice and signposting; and peer support. Carers will receive the option of having their cameras switched on or off during the sessions and will be encouraged to use the chat function to ask questions if unwilling to speak in front of the group.

Five programme rollouts of HELP (six weeks each) will be delivered across each of the three collaborating nations over a year period, with twelve carers per rollout (60 carers per nation; 180 carers in total). Rollouts will be separated by four-week windows, which will account for staff holidays and provide recruitment intervals for the subsequent rollout.

#### 2.2.10 Implementation strategy.

The development of implementation strategies is an evidence-based method of promoting uptake of a novel intervention by healthcare professionals [21]. Accordingly, guided by the NPT framework [14], we engaged with healthcare professionals (approximately forty HF nurses and cardiologists) across our five collaborating sites in the UK to co-develop a multi-faceted implementation strategy for HELP, which is theoretically grounded across: 1) Coherence (*i.e.,* does the intervention make sense?), 2) Cognitive participation (how do people engage?), 3) Collective action (how do people work with the intervention?), and 4) Reflexive monitoring (how do people appraise the intervention?). This iterative, developmental process spanned three, one-hour stakeholder meetings, resulting in the identification of four principal implementation agents (1. HELP Delivery Staff, 2. Referral staff, 3. Clinical Leadership, and 4. Carers). A multi-faceted implementation strategy was developed based on clinical and academic input, which employs tailored methods of supporting HELP implementation for each of the implementation agents (S4 Table).

Depending on preference, intervention delivery staff will receive online or in-person training from the project team, which will employ an interactive approach including content talks, case scenario discussions, and role plays. This training programme will familiarise delivery staff with intervention design and cover essential skills for implementation (*i.e.,* effective communication, building trusting relationships, managing conflict, and delivering behaviour change techniques). These staff will also receive access to standardised intervention materials and support documents (*i.e.,* intervention protocol and fidelity checklist), with regular support and monitoring by the project team at and between Steering Group meetings. As detailed in S4 Table, the other key implementation agents (*i.e.,* HELP referral staff, clinical leadership, and carers) will be supported via communication with the research team, information from project documents (*i.e.,* screening log and information sheets), and monitoring at Steering Group meetings.

### 2.3 Outcomes

**Fig 3:**
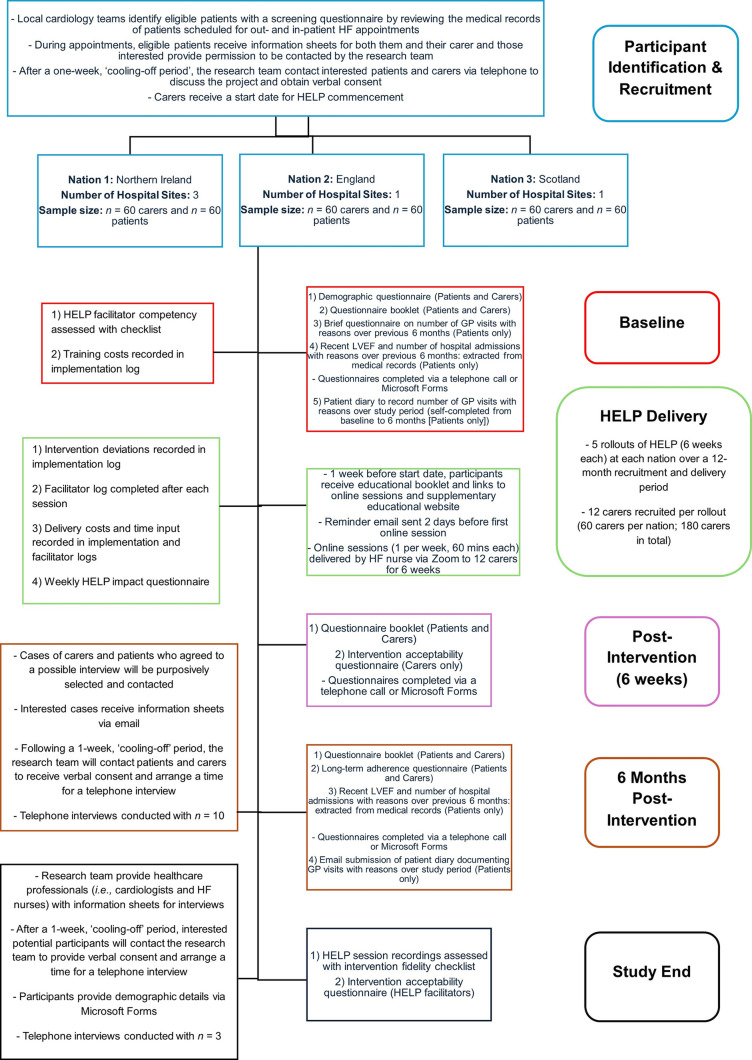
Study Flow and Data Collection Process. **Legend** UK = United Kingdom; HELP = HEart faiLure carer support Programme; GP = General Practitioner; HF = heart failure, and LVEF = left ventricular ejection fraction.

An overview of the study flow and data collection process is presented in Fig 3. Guided by CFIR and NPT integration [13,14], quantitative (*i.e.,* questionnaires, logs, and checklists) and qualitative (*i.e.,* interviews) data will be collected across the participants at five time points: 1. Baseline, 2. Intra-intervention, 3. Post-intervention (six weeks), 4. Six months post-intervention, and 5. Study end (eighteen months). For questionnaire data, participants will have the option of completion via Microsoft Forms or telephone calls, depending on preference.

#### 2.3.1. Demographic characteristics.

Participants will be asked to complete a demographic questionnaire at baseline (*i.e.,* age, gender, ethnicity, education level, employment status, and duration of care provision). These data will be used to contextualise the samples and findings.

#### 2.3.2 Process evaluation.

The multi-faceted implementation strategy will be assessed via a process evaluation of acceptability, fidelity, and context of HELP delivery across the collaborating clinical sites. Consistent with the framework published by the National Institutes of Health Behaviour Change Consortium [22], the fidelity examination will include the following:

1. Study design: Protocol and intervention deviations will be documented and reported in implementation and HELP delivery staff logs.
2. Provider training: Following training, intervention competency of HELP delivery staff will be assessed during a ‘role play’ session with a fidelity checklist.
3. Intervention delivery: Online support group session recordings will be evaluated using an intervention fidelity checklist spanning: 1) Interactional style (*i.e.,* carer-centred approach); 2) Intervention components covered and those omitted; 3) Components added; 4) Dosage (*i.e.,* session duration and attendance); and 5) Competency (*i.e.,* maintenance of skill set).
4. Treatment receipt and enactment: Carers’ understanding of the information provided and ability to implement the skills and recommendations delivered by HELP will be investigated via bespoke, weekly HELP impact (completed intra-intervention) and long-term adherence questionnaires (completed six months post-intervention), along with deeper exploration via interviews (discussed below).

Contextual factors will be assessed via data (*i.e.,* ‘background noise’ and protocol deviations) recorded in the implementation and HELP delivery staff logs, along with an evaluation of bespoke, intervention acceptability questionnaires completed by carers and HELP delivery staff. Individual, semi-structured interviews (approximately 60 minutes in duration) will also be conducted via telephone calls with participants at 6 months or study end (eighteen months), which will add rich, qualitative data to the assessment of acceptability, fidelity, and context. A “laddered-style approach” will be implemented in the interviews to explore emerging ideas [23], with the topic guides (S5 Text) based on the domains of CFIR and NPT [13,14]. Approximately thirty-three interviews will be conducted with carers (number (*n*) = 10), patients (*n* = 10), HELP delivery staff (*n* = 3), and healthcare professionals (*n* = 10 in total, *i.e., n* = 5 HF nurses and *n* = 5 cardiologists). This sample size is based on recommendations for a study of this scope [24]. A purposive sampling strategy [25] will maximise representation and diversity across the samples (*i.e.,* age, gender, ethnicity, education, and socio-economic status), with equal numbers of cases recruited across the collaborating sites. All interviews will be audio-recorded before verbatim transcription.

#### 2.3.3 Economic evaluation.

The economic cost of intervention implementation in the UK will be estimated via the collection of the following data from implementation and HELP delivery staff logs:

1) Training costs and time input for supervision of HELP delivery staff.
2) The time, expenditure, resources required, and adaptations made for HELP implementation by delivery staff.

#### 2.3.4 Participant outcomes.

The short and long-term impact of HELP on outcomes for carers and patients will be assessed across data from validated questionnaires, patient diaries, and medical records (see Table 2). Participant outcome data will be collected at three time points: 1. Baseline, 2. Post-intervention (week six), and 3. Six months post-intervention.

**Table 2:** Participant Outcomes and Measurement Instruments.

Sample	Outcome	Measurement Instrument	Time Point		
			Baseline	Post-Intervention (Week 6)	6 Months Post-Intervention
**Carers**	Carer Burden	Zarit Burden Interview, Short Form [26]	X	X	X
	Carer Preparedness	Preparedness for Caregiving Scale [27]	X	X	X
	QoL	12-Item Short Form Survey [28]	X	X	X
	Anxiety and Depression	Hospital Anxiety and Depression Scale [29]	X	X	X
	Stress	Perceived Stress Scale [30]	X	X	X
	Social Support	Multidimensional Scale of Perceived Social Support [31]	X	X	X
**Patients with HF**	Disease-specific, QoL	Minnesota Living with HF Questionnaire [32]	X	X	X
	Generic, QoL	EuroQoL Five Dimension Questionnaire [33]	X	X	X
	GP Visits with Reasons over Previous 6 Months	Bespoke, Recall Questionnaire at Baseline and Patient Diary Self-Completed from Baseline to 6 Months	X		X
	Most Recent LVEF Measurement	Medical Record	X		X
	Hospital Admissions with Reasons over Previous 6 Months	Medical Record	X		X

**Legend** QoL = Quality of Life; HF = Heart Failure; GP = General Practitioner; and LVEF = Left Ventricular Ejection Fraction.

### 2.4 Data management

Data management will comply with the requirements of the General Data Protection Regulation (2018). Personal data will be pseudonymised, with the master list linking participant identification numbers to personal information stored securely and separately from other data in a locked office within the School of Nursing and Midwifery, Queen’s University Belfast. A pseudonymised case report form (CRF) will serve as a consolidated resource documenting the data collected from each participant, with access limited to authorised individuals from the central research team at Queen’s University Belfast. These individuals will be responsible for data entry and validation at the point of entry into the CRF. Data from collaborating sites (*i.e.,* information from patient medical records) will be sent to the central research team via encrypted emails containing pseudonymised, password-protected data collection forms. Transcripts of audio-recordings will be pseudonymised, with transcription undertaken by a specialist service who has completed a data sharing and confidentiality agreement. All audio-recordings will be destroyed after the transcripts have been checked for accuracy. All paper data will be stored in a Master File and Site Files held within locked offices at the School of Nursing and Midwifery, Queen’s University Belfast and across the collaborating centres, respectively. All electronic data will be password protected and stored on an encrypted and password protected computer, inside a locked office at the School of Nursing and Midwifery, Queen’s University Belfast. On request, access to data will be granted for authorised representatives of the sponsor (Queen’s University Belfast) and regulatory authorities to facilitate study-related monitoring, audits, and inspections.

### 2.5 Data analysis

#### 2.5.1 Quantitative data.

Quantitative data analysis will be managed on Statistical Package for the Social Sciences (IBM SPSS Statistics, Version 31). Continuous data will be presented as mean ± standard deviation, with discrete data reported as absolute numbers and percentages. Categorical data will be displayed as frequency/ percentages. For participant outcomes, repeated measures analysis of variance will be used to investigate the effect of time point on outcome scores. Due to the absence of statistical power, the results will be used to identify preliminary evidence of promise for the intervention across different participant outcomes, with potential effect sizes generated to inform the required sample size for a future definitive evaluation.

#### 2.5.2 Qualitative data.

Qualitative data will be managed on NVivo (QSR International Pty Ltd. Version 15). Transcripts will be analysed using adaptive framework analysis [34] based on the CFIR and NPT domains [13,14], whilst allowing the integration of data that could not be placed in pre-specified categories. An a priori codebook will be established to guide the analysis. Definitions of CFIR and NPT domains will be tailored to the project, which will improve coder consistency. A member of the central research team will undergo data familiarisation before mapping major themes to the CFIR and NPT domains. Anonymised quotes from participants will be used to support the generation of themes. Differences between and within regions will be compared. A second member of the central research team will independently analyse and verify a random selection of transcripts. Subsequently, the results will be reviewed by the wider project team, with discrepancies discussed until consensus agreement.

#### 2.5.3 Data integration.

Data integration will be conducted according to the recommendations of Farmer et al. [35]. Guided by CFIR and NPT domains [13,14], this integrative analysis will involve the development of a convergent coding matrix to identify synergies (key findings) across the analytic components (*i.e.,* acceptability, fidelity, context, and participant outcomes). Two members of the central research team will integrate quantitative and qualitative data separately, with discrepancies discussed and reviewed by the wider project team until consensus agreement.

#### 2.5.4 Economic analysis.

HELP delivery costs will be estimated according to resource utilisation data collected during the project and unit costs for resource use obtained from published national or National Health Service sources [36]. Resource use will consist of time input from HELP facilitators, supervision for facilitators, training costs for facilitators, and consumables/ adaptations required for HELP delivery. Delivery costs will be estimated at session and programme-level and reported in pounds sterling (£).

### 2.6 Ethical considerations

This study has been approved by the West Midlands – South Birmingham Research Ethics Committee (Reference: 25/WM/0110, Approved 07 August 2025) and the Health Research Authority (Reference: 353770, Approved 17 November 2025). Audio-recorded, verbal informed consent will be obtained from all participants by the central research team at Queen’s University Belfast. The study will be conducted according to Good Clinical Practice guidelines, whilst adhering to the General Data Protection Regulations and the research governance frameworks across the sponsor and collaborating sites. Given the low-risk nature of HELP (nurse-led, psychoeducational support) and the acceptability demonstrated in the pilot study [10], we do not expect any intervention-related harm for participants. However, as the study will involve the discussion of sensitive topics (*i.e.,* palliative care), we have established distress management protocols for implementation in the event of participant distress. We consulted our Steering Group (*i.e.,* carers, patients with HF, and healthcare professionals) on the development of this study protocol, procedures, and materials to mitigate any areas of ethical concern and ensure suitability for participants. This Steering Group will continue to provide monitoring and input over the study duration. Any protocol amendments will receive the required regulatory approvals before the central research team communicate important changes to the collaborating sites.

### 2.7 Dissemination policy

The results will be reported to the funder and shared in a lay format with participants. To broaden interest and draw attention to the capacity and requirements for scaling and regional implementation of HELP, we will seek to publish the findings in peer-reviewed scientific journals, along with delivering presentations at national and international stakeholder events and scientific conferences. Members of the Steering Group (*i.e.,* patients and carers) will guide the dissemination of results and be involved with co-authorship of papers and abstracts where possible.

### 2.8 Status and timeline of the study

This study has received approval from the West Midlands – South Birmingham Research Ethics Committee (Reference: 25/WM/0110, Approved 07 August 2025) and the Health Research Authority (Reference: 353770, Approved 17 November 2025), with recruitment, intervention delivery, and data collection expected to run from 01/04/2026–01/10/2027. To date, no challenges or changes have occurred.

### 2.9 Data availability

Upon project completion, anonymised data sets will be allocated a unique identifier and transferred to a research information management system (Pure) for long-term preservation, as per participant consent. These data will be publicly accessible and will contain links to the project and relevant publications.

## 3.0 Discussion

To the best of our knowledge, HELP represents the first co-designed, theoretically underpinned service for carers of patients with HF across the UK, which addresses a critical area of unmet emotional and educational need [3,4,8]. Whilst pilot work demonstrated intervention feasibility and preliminary evidence of improvements in carer-related outcomes [10], the results do not inform whether HELP would generate the same outcomes in real-world, clinical settings [37]. Moreover, prior to large-scale, routine implementation, it is vital to comprehensively understand the active components of an intervention, how closely real-world delivery follows what is intended, and any contextual pre-requisites for successful translation to clinical practice [13].

This study will shed light on the beforementioned uncertainties by evaluating the implementation and impact of HELP in real-world clinical environments across UK. Through conducting process and economic evaluations underpinned by implementation science frameworks (CFIR and NPT) [13,14], the study will generate a comprehensive understanding of the macro (*i.e.,* system-level) and micro (*i.e.,* individual-level) factors influencing successful implementation in clinical practice [15]. Moreover, the acceptability and costs of HELP delivery will be investigated, along with demonstrating preliminary intervention effects on patient and carer outcomes following real-world delivery. These findings will inform the design and conduct of a large-scale, type 2 hybrid (clinical effectiveness – implementation) study [38], which provides crucial evidence for advancing HELP towards routine rollout across the UK.

This body of work may lead to national adoption and embedding of a novel intervention that addresses a significant area of unmet need for carers of patients with HF. Through the empowerment of carers, HELP may also have a positive impact on enhancing patient health (*i.e.,* reduced hospital readmissions and improved QoL) by enabling carers to effectively support patients with self-management activities [2]. Moreover, the online delivery of HELP supports the current digitalisation of health care services [39], whilst facilitating greater geographical spread and circumventing the carbon emissions of travel. If successful, HELP has the capacity to be expanded beyond the UK and may also be adapted to other disease and carer populations.

### 3.1 Strengths and limitations

This mixed-method, implementation study will subject a novel, co-designed intervention to real-world process and economic evaluations across three nations of the UK (Northern Ireland, England, and Scotland). Underpinned by implementation science frameworks (CFIR and NPT) [13,14], the holistic findings will underscore key factors influencing successful translation to clinical settings. The collaborating hospital sites cover a range of settings and characteristics (*i.e.,* urban, rural, education level, socio-economic status, and ethnicity), which will maximise the diversity of the recruited sample. Through establishing beacon sites, the study will facilitate a real-world, clinical platform to model programme implementation, which will support future scaling. Whilst the study is limited to English-speaking participants, the Steering Group will ensure HELP is optimised for future language translation. Whilst digital access was not a barrier to participation in the pilot study [10], we have a stock of digital devices to provide to those without access in the current study. Given the non-randomised design and lack of statistical power, the participant outcomes will not represent definitive findings. This assessment will be conducted in a future study. Finally, HELP is currently limited to carers, future work will expand this intervention to include patients.

## Supporting information

S1 Checklist SPIRIT 2025 Checklist. (DOCX)

S2 Checklist Standards for Reporting Implementation Studies: The StaRI Checklist For Completion. (DOCX)

S3 Text HELP Educational Booklet. (PDF)

S4 Table HELP Multi-Faceted Implementation Strategy Mapped to the NPT Framework. (DOCX)

S5 Text Topic Guides for Semi-Structured Interviews with Participants. (DOCX)

S6 Text Copy of the protocol that was approved by the ethics committee. (DOCX)

S7 Text Copy of ethical approval from West Midlands – South Birmingham Research Ethics Committee. (PDF)

S8 Text Copy of approval from Health Research Authority (PDF)
