# Supplementary material for: Evaluating the implementation and impact of the HEart faiLure carer support Programme (HELP) in the United Kingdom: A study protocol for a multi-centre, mixed-method, implementation study
Source: PLoS One. 2026 Apr 17;21(4):e0347037. doi: 10.1371/journal.pone.0347037 (PMC13089873; doi:10.1371/journal.pone.0347037)
Supplement: S3 Text — (PDF) [file pone.0347037.s003.pdf]

A GUIDE FOR CARERS

Support For Carers Of Patients With Heart Failure

QUEEN'S UNIVERSITY BELFAST

British Heart Foundation

Chest Heart & Stroke

About this booklet

This booklet is based on previous research and has been developed with carers of people with heart failure. It gives information and practical advice to improve how carers look after themselves and their loved ones with heart failure. Quotations from previous research with carers are used throughout.

This booklet is suitable for any family carers, as there is increasing awareness that carers have needs too.

Contents (read before online support group)

1. **What is heart failure?** (before Session 2)
2. **Planning for the future** (before Session 2)
3. **Looking after yourself** (before Session 3)
4. **Communication** (before Session 4)
5. **Your role as a carer** (before Session 5)

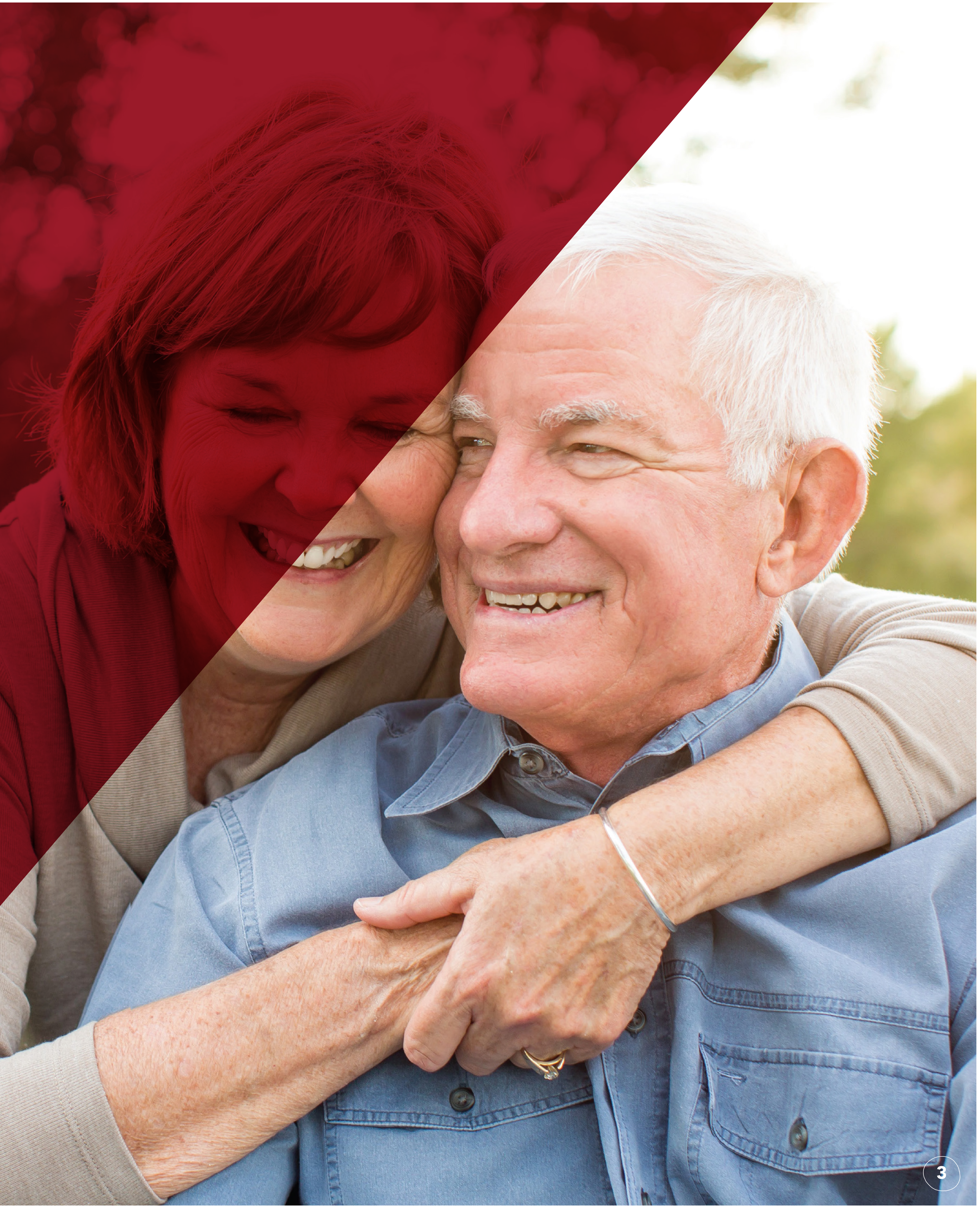

QUEEN'S UNIVERSITY BELFAST

British Heart Foundation

Chest Heart & Stroke

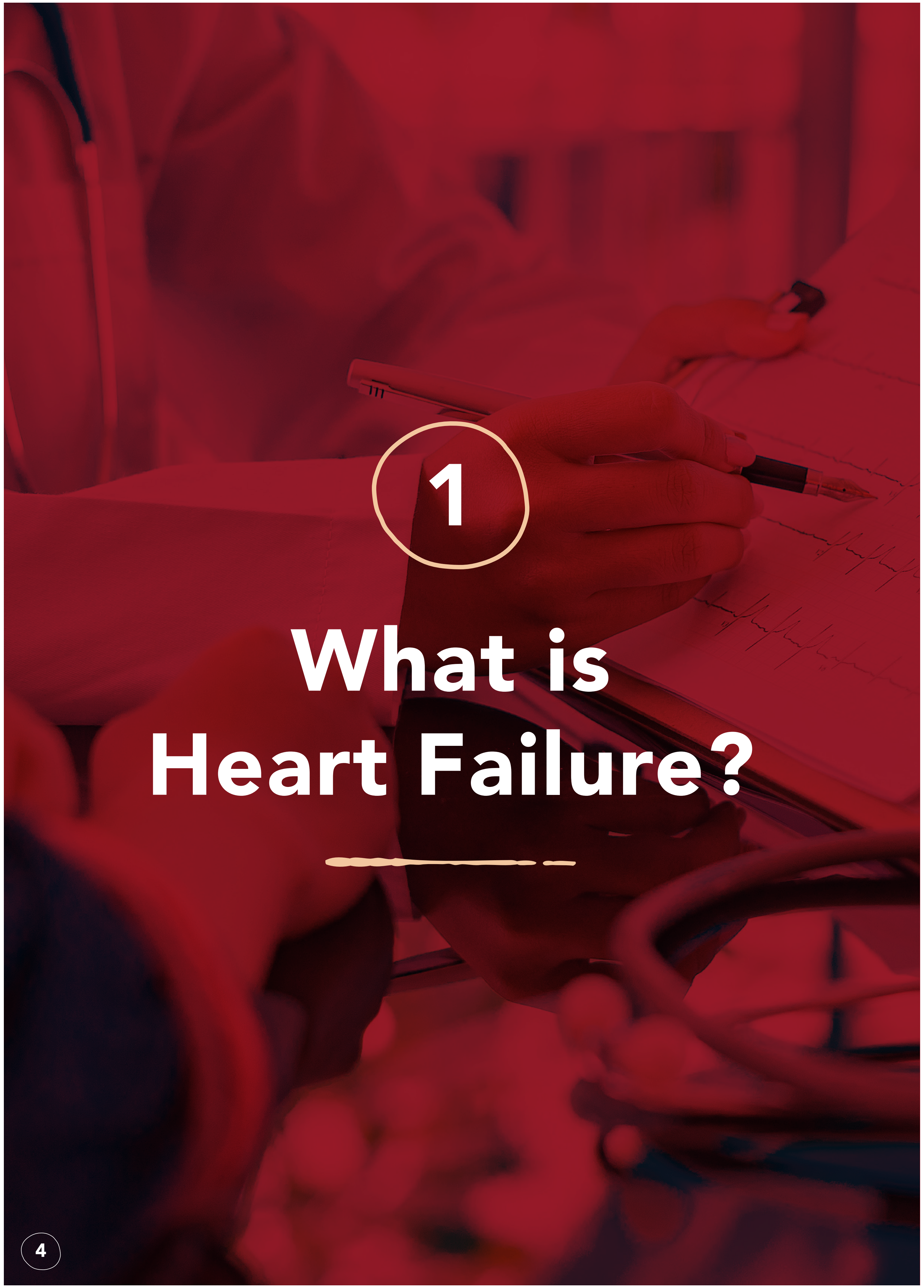

1

What is Heart Failure?

Heart failure means that the heart cannot pump blood around the body as well as it used to. This can cause symptoms like breathlessness, tiredness, and swollen ankles. This booklet can help you understand how to relieve these symptoms and improve quality of life.

- Heart failure can happen as a result of a heart attack, high blood pressure, or another cause.
- It causes the heart to weaken, which means blood cannot be pumped around the body properly.
- Fluid can gather in the legs, ankles, and other parts of the body like the lungs.
- Heart failure can occur at any age, but is most common in people over 65 years old. It progresses at different rates for each person.
- There are good treatments available for heart failure, which can relieve symptoms. However, a heart transplant is the only cure, but this is not suitable for everyone.
- Supportive care is available and talking with your loved one and health care professional can help plan for the future.

> **“I don’t think I knew what it meant. To be honest I just thought he had a bit of fluid on his lungs that he needed these tablets and that would keep it at bay.”**

Heart failure can affect each person differently however with careful management and monitoring of symptoms quality of life can be improved.

Signs to look out for

Keeping an eye on these things are important:

Shortness of breath: this is caused by a build-up of fluid in the lungs. Some people develop a troublesome cough with frothy sputum.
Extra pillows in bed at night can help.

Swollen ankles, legs or tummy: this can happen due to the fluid build-up.
Encourage your loved one to be seated and put their legs on a stool to raise them. Check your loved one hasn't missed any medication. Encourage them to get some light activity they can tolerate, like walking about.

Weight gain: sudden weight gain (2kg over 3 days) can happen due to fluid build-up in the body.
Be cautious with their liquid intake, encourage your loved one to weigh themselves every morning.

Dizziness: reduced blood flow to the brain can lead to feeling dizzy or lightheaded.
Make sure your loved one does not try to get up from a sitting position too quickly. Report dizziness to a nurse or doctor, especially if it causes your loved one to stumble or fall.

Increased heart rate: the heart beats faster to compensate for the lack of pumping power.
Tell a nurse or doctor if this becomes a problem.

Fatigue: the heart's inability to pump blood properly means that the muscles tire more quickly.
As a result extreme tiredness and loss of energy may be experienced.
This fatigue can be difficult for you and your loved one. Try to be patient with them and encourage your loved one to get fresh air, engage in light exercise daily, and pace their activities.

Reduced appetite: some people can accumulate fluid around the tummy which makes them feel full or 'bloated'.
Offer smaller portions or snacks throughout the day. Monitor salt intake and reduce as much as possible.

You know your loved one best and could notice even small changes, so it is important to let a doctor or nurse know if you have any concerns about changes in symptoms.

If you would like to find out more information about heart failure then visit:

www.heartfailure.org

www.heartfailurematters.org

www.cardiomyopathy.org

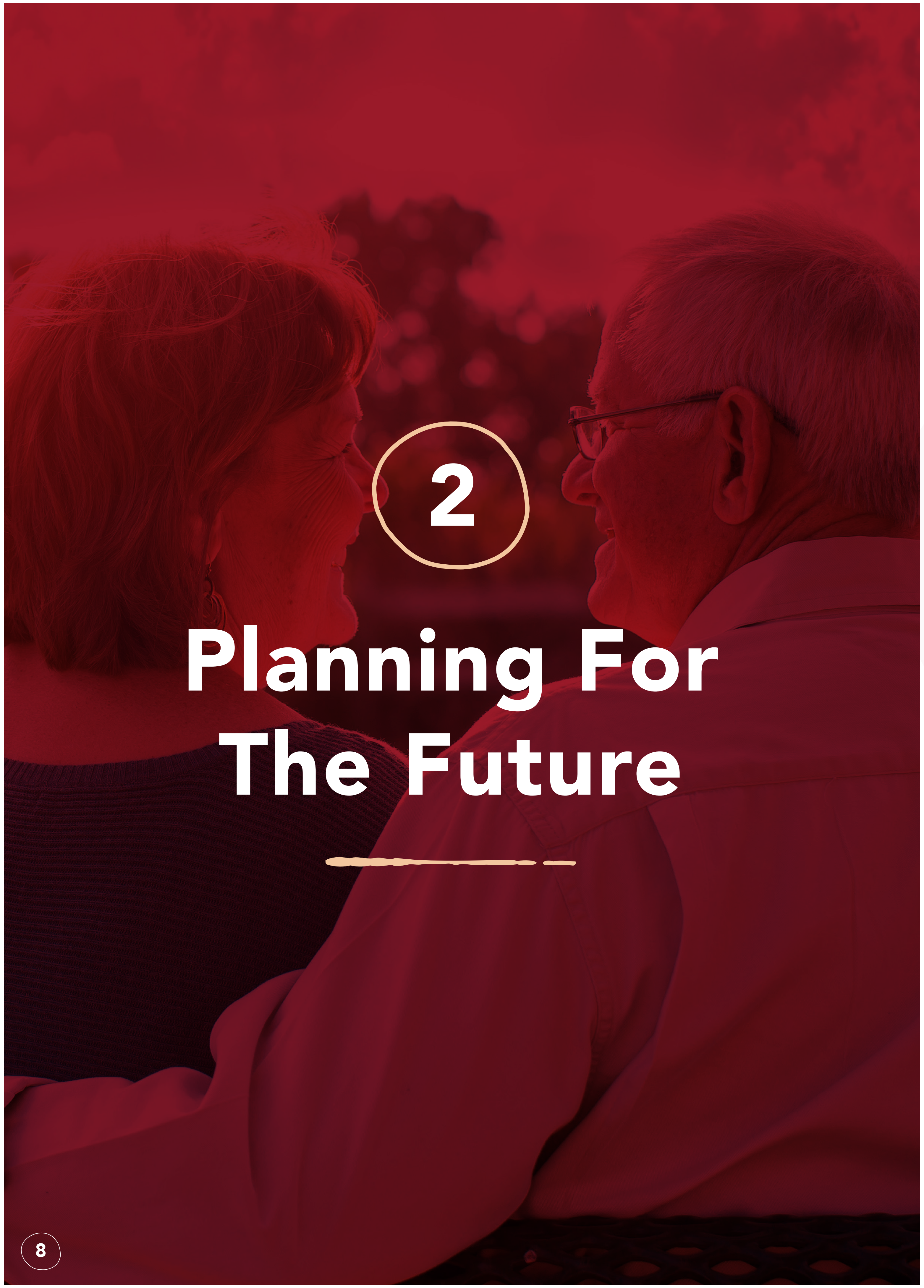

Heart failure is a progressive and unpredictable condition that can be managed at home long-term. In some situations it can be difficult when caring for your loved one, but help is available.

When your loved one is discharged from hospital it can be difficult to understand and adjust to their needs. There may be a fear of readmission to hospital, knowing how to monitor and manage their symptoms, the medications prescribed and how to care for your loved one.

> **"It was a very stressful time, I think I was at that stage where I wanted somebody to tell me what to do"**

Here are some things you can do to make life after a hospital discharge better:

- Ask if you can discuss the discharge plan with the healthcare team before leaving the hospital. This way you can share any concerns or worries you may have about looking after your loved one at home
- Bring a notepad with you to write down any information you are afraid you might forget or ask permission to record the conversation on your mobile phone.
- Ask your doctor or heart failure nurse for information on what would be an "emergency situation" and who to contact
- Ask for advice on how to manage any further deterioration in your loved one's condition

Caring for someone with declining symptoms and quality of life can be difficult. Frequent hospital appointments, changes to medications and changing emotions can put a strain on your relationship. Take 'time out', or accept offers of help from friends and family.

In some situations, there may be a point when your loved one's healthcare professional no longer has a solution. Although this may be an emotional time, it is important to talk about long term needs. Discussing this with a GP, heart failure nurse, or doctor can help you and your loved one prepare for the future.

Here are a few signs that indicate your loved one may be at this point:

- Increased breathlessness on minimal physical activity
- Requiring numerous pillows to sleep and awakening during the night with breathlessness
- Sudden change in weight - increase or decrease
- Experiencing anxiety
- Frequent palpitations

If these symptoms are new or have got worse then you should contact your heart failure nurse, doctor or GP. If they are severe, you may need to call 999 for an ambulance.

Supportive and Palliative Care

Because heart failure is a chronic condition, treatment should always focus on controlling symptoms and improving quality of life. Active treatment and palliative care go hand in hand. As time progresses tablets and other treatments may not work as well as they once did and symptoms of heart failure may be harder to manage.

Supportive and palliative care is about managing symptoms and helping you and your loved one get the most out of every day and keeping your loved one comfortable.

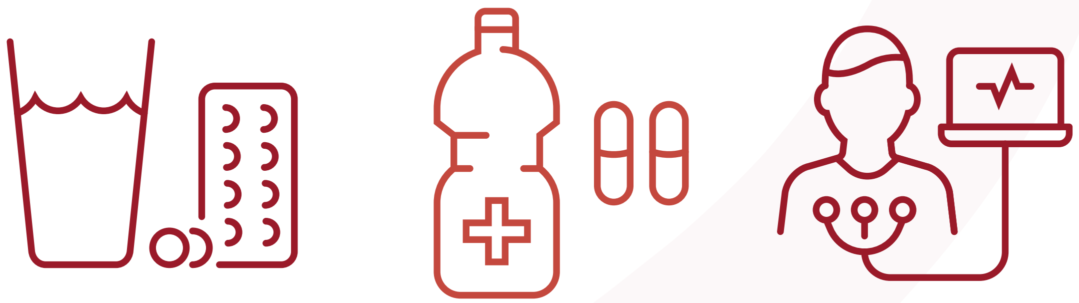

You might discuss things with the palliative care team like:

- Treatment options
- Blood tests
- Medications

Caring for someone in the last stages of their life can be stressful and hard to deal with; however there is always help and support available. Having the right information, practical help and emotional support will make things easier to deal with.

Marie Curie
www.mariecurie.org.uk

Northern Ireland Hospice
www.nihospice.org/our-services/adult-services/bereavement-support/

Although caring for someone with heart failure can be difficult, carers have told us it can also be a rewarding and positive experience

Here are some things to remember if you ever feel like you are having a bad day:

- Being a carer allows you to spend valuable time with your loved one and make precious memories
- It can bring you and your loved one closer together and strengthen your relationship
- It can help you develop skills to manage your own health better
- It can give you the chance to discuss the things that are important to you and your loved one and plan ahead

"We are still very much in love... I still care for her an awful lot and am happy doing it. There is a lot of satisfaction in it for me."

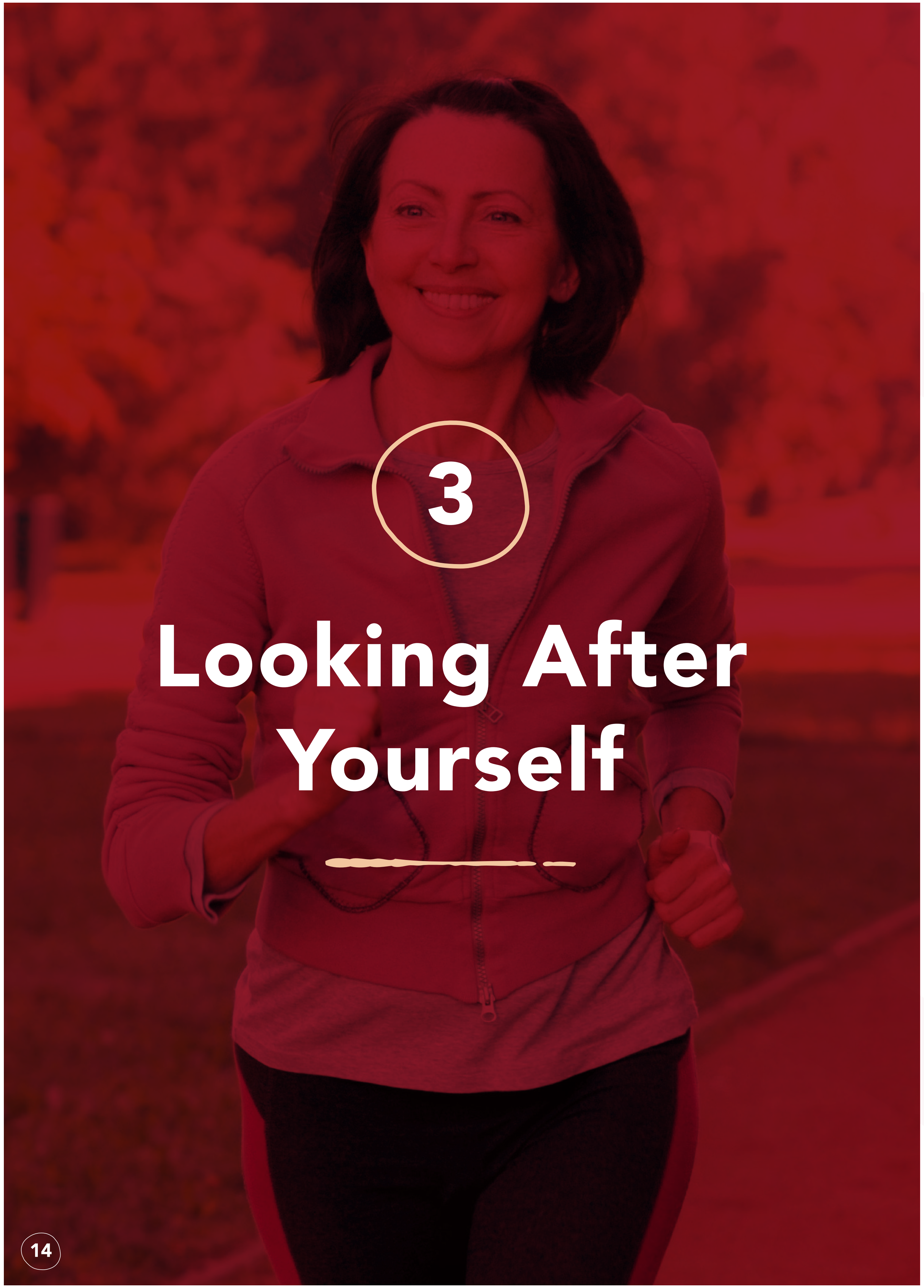

3

Looking After Yourself

Looking after your own physical and mental health is extremely important and can make a huge difference to how you cope with being a carer.

Eating healthily, sleeping well and remaining active can help you to stay on top of your own health.

Remember your own health needs and attend check-up appointments. If you stay well it will be easier to keep your loved one well too.

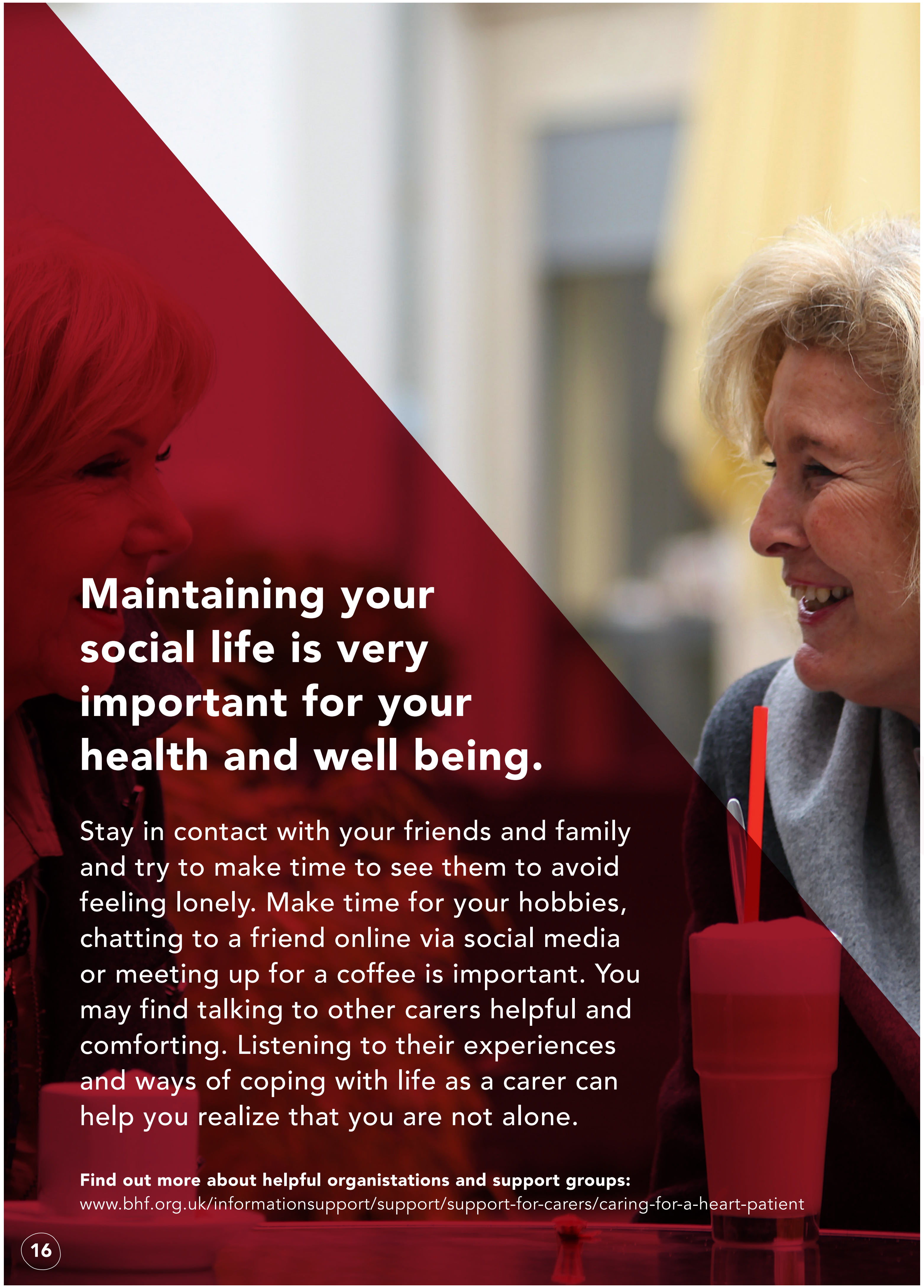

Maintaining your social life is very important for your health and well being.

Stay in contact with your friends and family and try to make time to see them to avoid feeling lonely. Make time for your hobbies, chatting to a friend online via social media or meeting up for a coffee is important. You may find talking to other carers helpful and comforting. Listening to their experiences and ways of coping with life as a carer can help you realize that you are not alone.

Find out more about helpful organisations and support groups:
www.bhf.org.uk/informationsupport/support/support-for-carers/caring-for-a-heart-patient

Emotions

Caring for someone can bring out different emotions you may not have experienced before. Everyone reacts differently, however you should remember that it is okay to have a bad day. Sharing how you feel with a friend, family member or health care professional may provide relief.

When caring for someone everyday it is natural to experience ups and downs. However if you are having more bad days than good, then speak to your doctor. Try talking things over with your loved one or a close friend, without knowing how you feel they can't help. Carers, like everyone can become depressed and may need help.

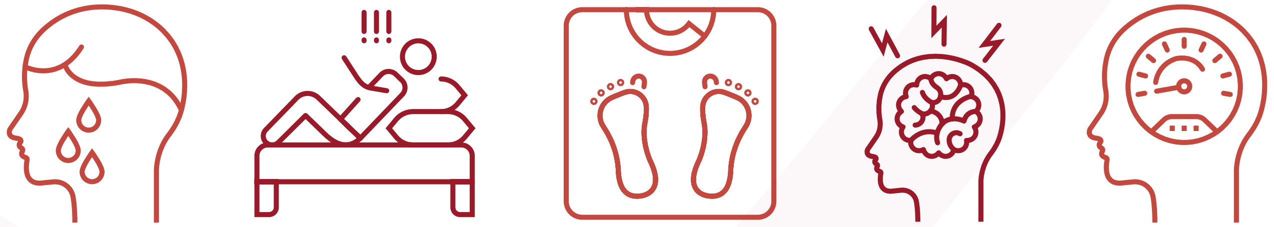

These are some signs to look out for:

- Feeling frequently sad or short-tempered
- Finding it difficult to sleep or wakening up very early
- Losing weight due to loss of appetite or weight gain
- Feeling worthless
- Losing interest in things that you used to enjoy

Stress

Life as a carer can be hectic. You may have to manage your home life and work life while being responsible for your loved one. It is important to recognise when things are becoming too much, in order to take the right steps to improve your health.

These are some signs to look out for:

- Waking up early or during the night
- Feeling tired and having no energy
- Poor concentration
- Feeling angry or irritable
- Experiencing low moods

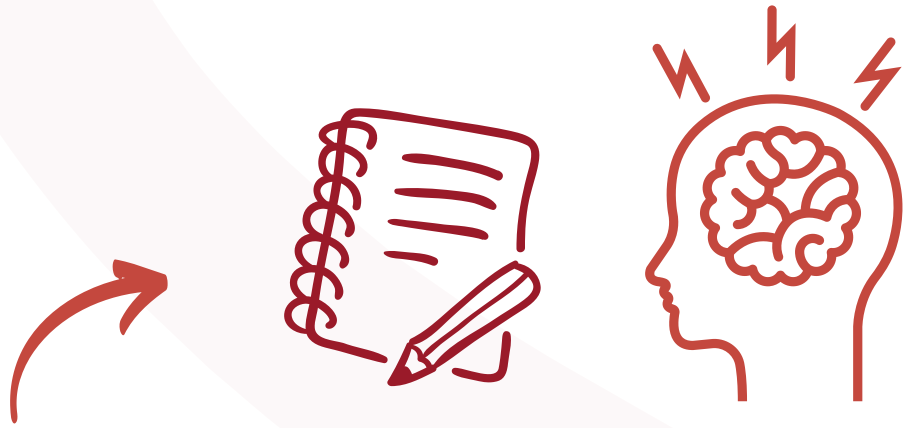

> "A key way of dealing with stress is to write down what you think the trigger is that is causing it. This will help you to identify the problem, address it and find the best way to deal with it."

Anxiety

It is normal to worry about your loved one's health and how you are managing as a carer. However, if your concerns are starting to affect your daily life then you should speak to your doctor or your loved one's heart failure nurse.

Some signs to look for are:

- Are you becoming upset more easily?
- Can you no longer control your worries?
- Do you have a tendency to think the worst in most situations?

Here are some things you can do to help if you start to experience any of these emotions:

- Try to make time for exercise- even a walk every day can clear your head.
- Set aside some time every day to do things you enjoy
- Take a break from caring, ask a relative to help out or look into local respite care
- Join a carer's support group
- Set realistic goals as trying to solve everything at once is hard
- Let your doctor know about how you are feeling at appointments

IF YOU EXPERIENCE ANY SIGNS OR FEELINGS DESCRIBED IN THIS CHAPTER THEN YOU SHOULD MAKE AN APPOINTMENT TO SEE YOUR DOCTOR TO DISCUSS AVAILABLE SUPPORT.

There are a number of organisations who can provide help in the home, to give you a break:

Crossroads Care NI
They provide help at home for carers.
TEL: **028 9065 3080**
EMAIL: **info@crossroadscare.co.uk**

HSC Trust / NHS
Your local HSC Trust / NHS can also provide services to help including residential care, day time and evening sitting, day centres, carer away days and also carer support groups.

British Heart Foundation
www.bhf.org.uk/informationsupport/support/support-for-carers/caring-for-a-heart-patient

Carers Trust
carers.org/help-for-carers/carer-services-near-you

Heart Failure Matters
www.heartfeilurematters.org

“Sometimes I would like to chat with somebody who is in a similar situation.”

For emotional support there are helplines you can call if you need someone to talk to or get some advice from:

Samaritans
TEL: **08457909090** (open 24 hrs)

Mind Infoline
TEL: **03001233393** (open 9am to 6pm mon to fri)

Anxiety UK helpline
TEL: **08444775774** (open 9:30am to 5:30pm mon to fri)

The Macmillan Support line
TEL: **0808 808 00 00** (open 9am to 8pm mon to fri)

CAUSE Carer helpline
TEL: **0845 6030291**

British Heart Foundation Heart Helpline
TEL: **0808 802 1234** (open 9am to 5pm mon to fri)
https://www.bhf.org.uk/helpline

Irish Heart Foundation helpline
TEL: **1800 25 25 50** (open 9am to 5pm mon to fri)

THERE IS ALWAYS SOMEONE OUT THERE TO TALK TO, WHETHER THAT IS A FAMILY MEMBER OR FRIEND, A SUPPORT GROUP, OR A HEALTH CARE PROFESSIONAL.

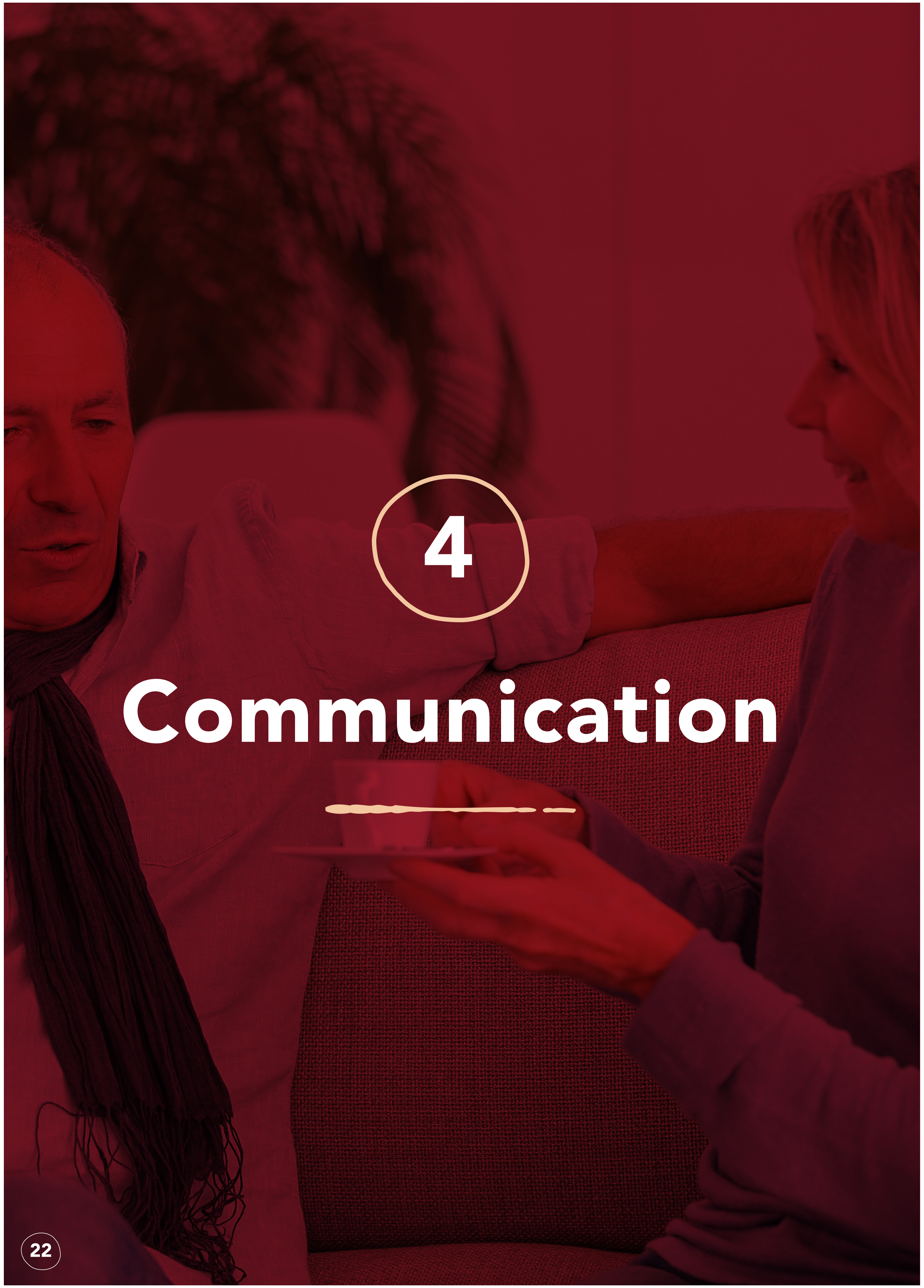

Communication is key when looking after someone with heart failure. Through communicating you can motivate your loved one to self-manage and regain some independence that may have been lost during their illness. It is also important to ask if you can provide information to the nurse or doctor at hospital appointments. You may have valuable information about your loved one's condition that may help the healthcare team with the management of symptoms.

> **"I should have said, I actually wanted to talk to a nurse or doctor"**

Some helpful tips to remember:

- Encourage your loved one to talk about how they are feeling and what you can do to help.
- Discuss what things they feel they can do that will not tire them out.
- Try to encourage your loved one to do as much as they can by themselves.
- Write down a list of any questions or concerns before an appointment with a nurse or doctor.
- Bring medication list to each appointment.
- Try to plan activities you both enjoy and your loved one can manage. For example, a trip to the park or coffee in a café.

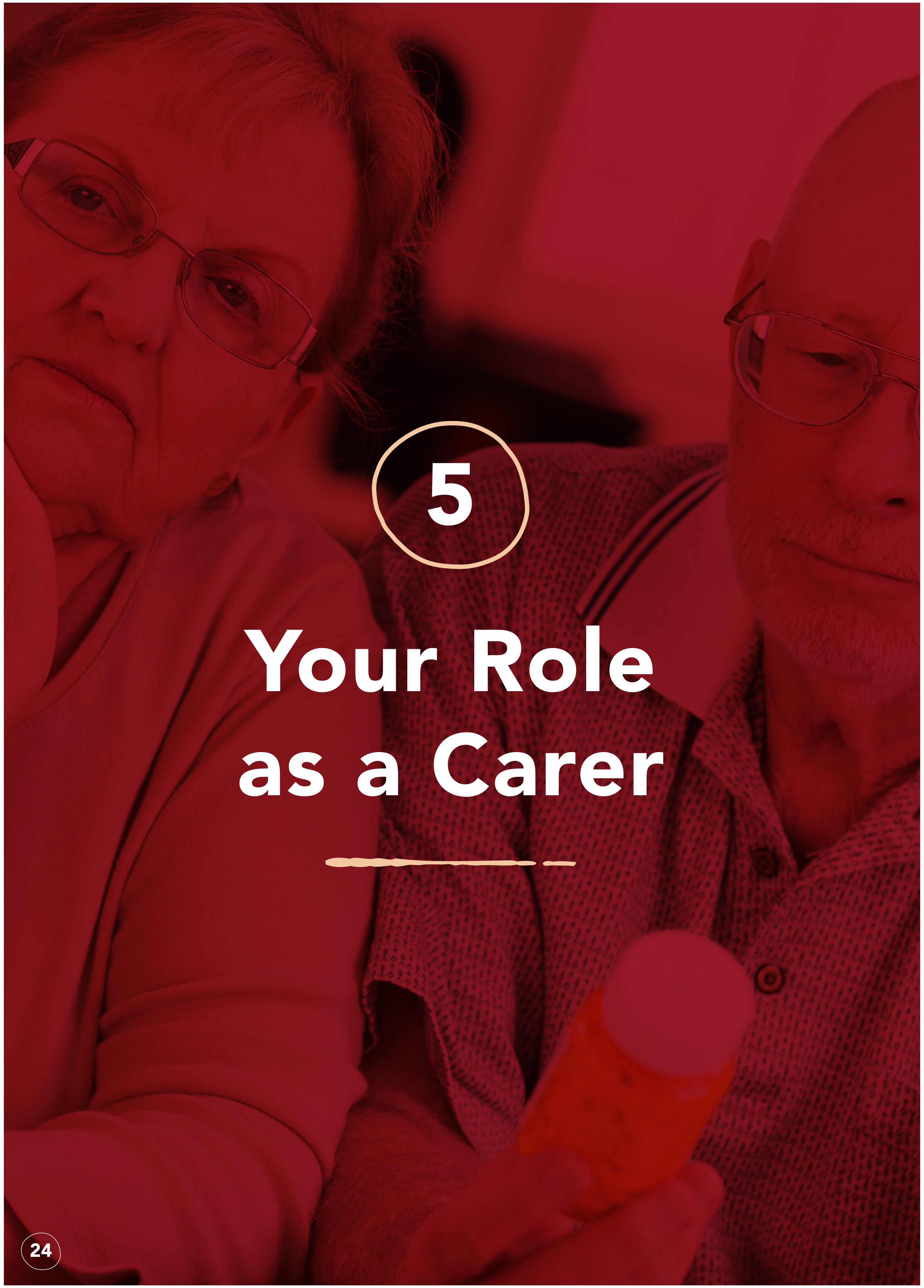

5 Your Role as a Carer

Your role is very important and by supporting your loved one you are making a real difference to their lives. You, the caregiver are key to keeping your loved one well, but to do that you must look after yourself first.

Caring for someone with heart failure can have its ups and downs. For some it may involve adapting to a new way of daily life and the responsibility of caring for someone. This may be in addition to the daily pressures of a busy family and home life. There is support available and accepting this will help you, it is not a sign of weakness.

> **Self-management is an important part of heart failure treatment. It is how patients care for themselves each day to prevent their heart failure from worsening. This can include taking medication, diet and exercise, monitoring symptoms, and contacting a nurse or doctor if / when needed.**

> **"Self-management by patients can help prevent hospital admissions, ease symptoms, and improve quality of life. Very often, it is the carer who supports the patient to achieve good self-management."**

Realise that you are making your loved one's life better and easier to manage. By helping your loved one be more independent, this will give you a chance to maintain or improve your own health.

Financial help

Finances are often a worry for many carers and can cause strain on relationships. However, there is support and advice available to help with this. You can request a Carer's Assessment to be carried out by your GP that will identify what areas you need support with and how best this can be provided.

A Carer's Assessment is usually a meeting with your GP or a member of your local Trust, where you will discuss things such as how much time you spend caring, what tasks you need help with and what aspects you have difficulty with. After the meeting there will be a letter sent out to you that outlines what support needs you have and what the Health & Social Care Trust / NHS can do to help you.

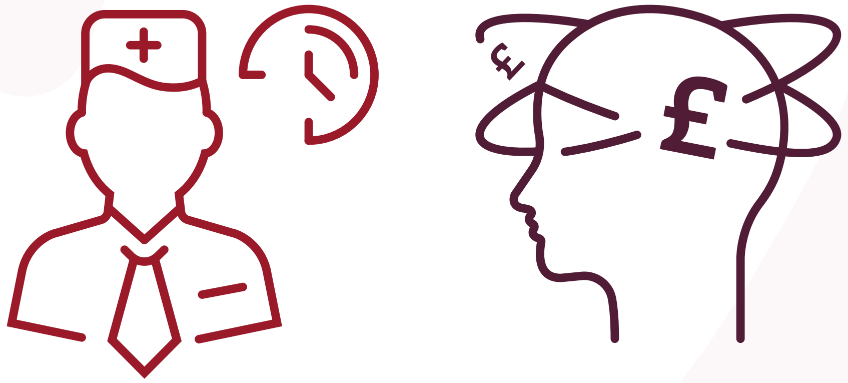

As well as financial support, they may be able to provide things like:

- disability equipment and adaptions to your home (e.g. a new shower fitted, stair lifts)
- help with housework and daily tasks
- a break from caring (respite services)

It is also important to let your friends and work colleagues know when you start caring for someone. This includes your employer as there may be times when you need to work flexibly or reduce your hours. You could need time off to accompany your loved one to hospital appointments. Discuss with your employer if they can offer you flexible working hours or change your shifts to better suit your needs.

For more information on how to arrange a Carer's Assessment please see:

Northern Ireland

www.nidirect.gov.uk/articles/assessments-carers

or

England or Scotland

www.nhs.uk/conditions/social-care-and-support-guide/support-and-benefits-for-carers/carer-assessments/

For information on the range of financial help and benefits available please contact:

Carers NI advice line TEL: 028 9043 9843

Belfast Carers Centre TEL: 0808 808 7777

Citizens Advice TEL: 028 9023 1120

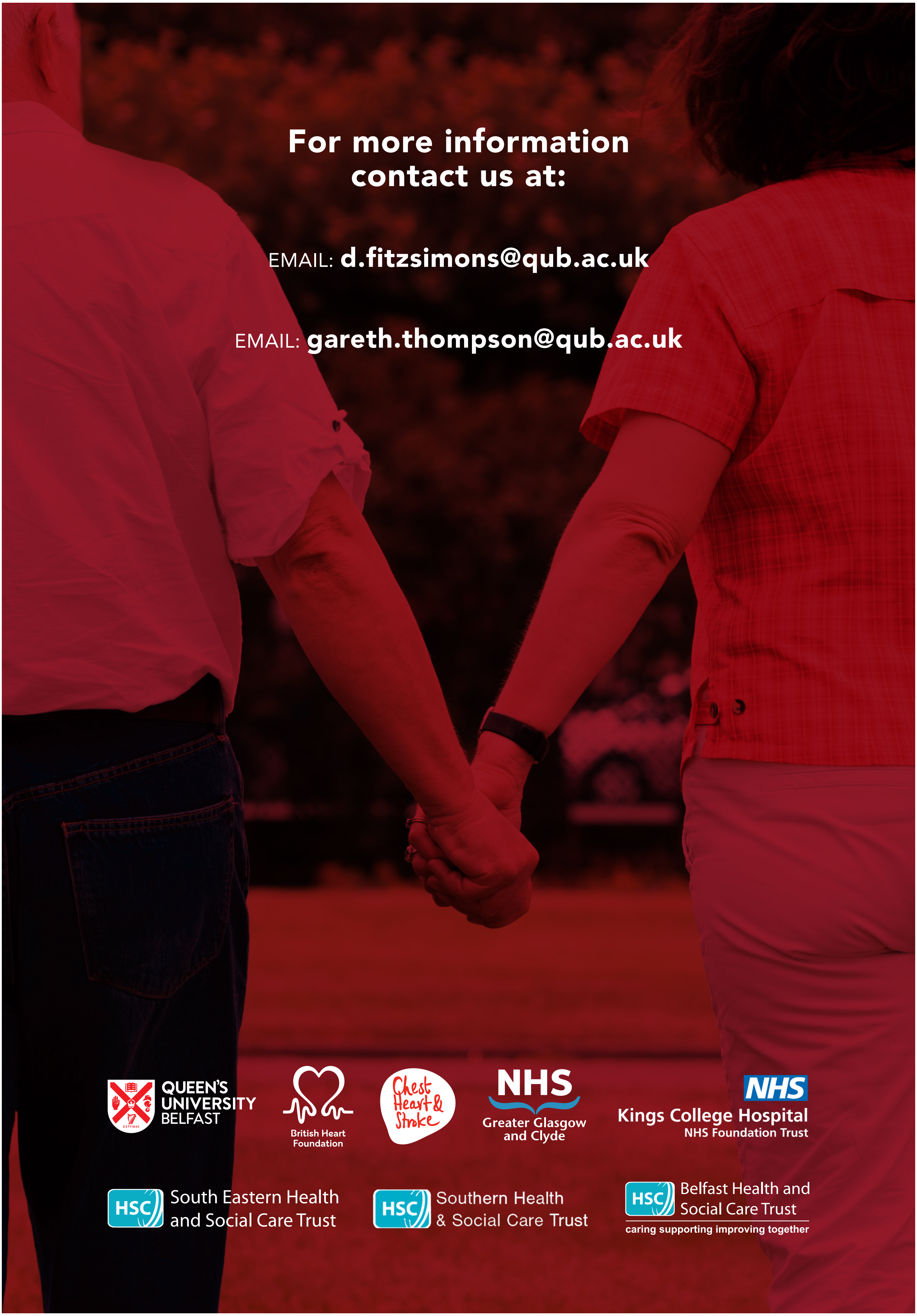

For more information contact us at:

EMAIL: **d.fitzsimons@qub.ac.uk**

EMAIL: **gareth.thompson@qub.ac.uk**

QUEEN'S UNIVERSITY BELFAST

British Heart Foundation

Chest Heart & Stroke

NHS Greater Glasgow and Clyde

NHS Kings College Hospital NHS Foundation Trust

HSC South Eastern Health and Social Care Trust

HSC Southern Health & Social Care Trust

HSC Belfast Health and Social Care Trust
caring supporting improving together
