# Supplementary material for: Evaluating the implementation and impact of the HEart faiLure carer support Programme (HELP) in the United Kingdom: A study protocol for a multi-centre, mixed-method, implementation study
Source: PLoS One. 2026 Apr 17;21(4):e0347037. doi: 10.1371/journal.pone.0347037 (PMC13089873; doi:10.1371/journal.pone.0347037)
Supplement: S4 Table — (DOCX) [file pone.0347037.s004.docx]

**S4 Table.** HELP Multi-Faceted Implementation Strategy Mapped to the NPT Framework.

| **NPT Construct** | **Implementation Strategies by Implementation Agent** | | | |
| --- | --- | --- | --- | --- |
|  | **HELP Delivery Staff (Specialist HF Nurses)** | **Referral Staff (Local Cardiology Teams)** | **Clinical Management (PIs across collaborating sites)** | **Carers** |
| **1. Coherence** (Does it makes sense?) | - Training  - Information from project documents  - Fidelity checklist | - Information from project documents  - Communication with Project Manager | - Members of the Steering Group  - Regular discussion with project team | - Information from project documents and intervention sessions  - Support from Project Manager |
| **2. Cognitive Participation**  (How do people engage?) | - Motivation and commitment established during training  - Documents and support available to facilitate delivery | - Project documents and Project Manager provide guidance  - Screening log to be completed | - Discussion with project team on potential importance of the intervention and guidance on what is required | - Encouragement from HELP delivery staff  - Attendance at online support group sessions  - Educational booklet and supplementary website to support  - Tailored support provided at online support group sessions |
| **3. Collective Action**  (How do people work with intervention?) | - Receive appropriate training  - Support from project team  - Fidelity checklist | - Actively referring participants  - Support from Project Manager | - Management displays support in meetings and encourages participation | - Attendance at online support group sessions as scheduled  - Engagement with educational booklet and supplementary website |
| **4. Reflexive Monitoring**  (How do people appraise intervention?) | - Appraised during meetings  - Fidelity checklist | - Appraised during meetings | - Appraised during meetings | - Opportunity for input / feedback across the online support group sessions |

**Legend:** NPT = Normalisation Process Theory; HELP = HEart faiLure carer support Programme; HF = Heart Failure; and PI = Principal Investigator.
