# Supplementary material for: Evaluating the implementation and impact of the HEart faiLure carer support Programme (HELP) in the United Kingdom: A study protocol for a multi-centre, mixed-method, implementation study
Source: PLoS One. 2026 Apr 17;21(4):e0347037. doi: 10.1371/journal.pone.0347037 (PMC13089873; doi:10.1371/journal.pone.0347037)
Supplement: S5 Text — (DOCX) [file pone.0347037.s005.docx]

**S5 Text. Topic Guides for Semi-Structured Interviews with Participants.**

***Topic Guide for Interviews with Carers***

| **Corresponding CFIR Domains and NPT Constructs** | **Semi-Structured Interview Question** |
| --- | --- |
| ***CFIR:*** *Intervention Characteristics*  ***NPT:*** *Coherence, Cognitive Participation and Reflexive Monitoring* | - Can you talk about your overall experience with HELP?   - Which elements did you like / dislike?   - How was your experience with the educational booklet?   - What did you think of the content and format?  - Can you discuss any strengths or weaknesses?  - Is there anything we should improve?   - What did you think of the HELP website (if applicable)?   - What are your thoughts on this resource being a website?  - Was it difficult to access or use?  - What did you think of the content and format?  - Can you discuss any strengths or weaknesses?  - Is there anything we should improve?  - Did you have a preference between the educational booklet and website?   - What are your thoughts on the online support group sessions?   - Would you change anything about the length or number of sessions?  - What did you think of the topics discussed?  - How did you find having other carers take part?  - Can you discuss any strengths or weaknesses?  - Is there anything we should change?   - What are your thoughts on the online parts of HELP?   - Do you think this is suitable for other carers?  - Is there anything in general we should change? |
| ***CFIR****: Outer Setting*  ***NPT:*** *Coherence and Reflexive Monitoring* | - Do you think there is a need for carers to receive HELP?   - How well does the programme meet these needs and how?   - How did HELP impact you?   - Did it influence how you cared for your loved one with heart failure?  - How long did you follow the advice and information for after the programme? |
| ***CFIR:*** *Inner Setting*  ***NPT:*** *Coherence, cognitive participation, and collective actions* | - Do you think HELP is something that would be suitable for hospitals across the UK to deliver? |
| ***CFIR:*** *Characteristics of Individuals*  ***NPT:*** *Coherence, cognitive participation, and collective action* | - How did you find the nurse who delivered HELP?   - Did he / she seem suitably trained?  - Was there anything the nurse could have done better?  - What skills should a nurse delivering the programme have? |
| ***CFIR:*** *Implementation Process*  ***NPT:*** *Coherence, cognitive participation, collective action, and reflexive monitoring* | - Can you think of any challenges to delivering HELP to all carers across the UK?   - Can you think of anything we can do to help overcome this?   - How can we best encourage carers to take part in HELP?   - How can we effectively tell them about the programme?  - Can you think of anything that might stop carers taking part?  - How may we overcome this? |
| *Interview Close* | - Could you summarise your experience with HELP in a few words? - Is there anything else you would like to discuss? |

CFIR = Consolidated Framework for Implementation Research; NPT = Normalisation Process Theory; and HELP = HEart faiLure carer support Programme.

***Topic Guide for Interviews with Patients***

| **CFIR Domains and NPT Constructs** | **Semi-Structured Interview Question** |
| --- | --- |
| ***CFIR:*** *Intervention Characteristics*  ***NPT:*** *Coherence, Cognitive Participation and Reflexive Monitoring* | - Can you talk about your overall opinion of HELP?   - Which elements do you like / dislike?  - What do you think of the educational booklet and HELP website?  - What are your thoughts on the online support group sessions?   - What are your thoughts on the online parts of HELP?   - Do you think this is suitable for carers?   - Is there anything in general we should change about the programme? |
| ***CFIR****: Outer Setting*  ***NPT:*** *Coherence and Reflexive Monitoring* | - Do you think there is a need for carers to receive HELP?   - How well does the programme meet these needs and how?   - How did HELP impact your carer and you?   - Did it influence how your carer supported you?  - How long did you both follow the advice and information for after the programme? |
| ***CFIR:*** *Inner Setting*  ***NPT:*** *Coherence, cognitive participation, and collective actions* | - Do you think HELP is something that would be suitable for hospitals across the UK to deliver? |
| ***CFIR:*** *Characteristics of Individuals*  ***NPT:*** *Coherence, cognitive participation, and collective action* | - What skills should a nurse delivering the programme have? |
| ***CFIR:*** *Implementation Process*  ***NPT:*** *Coherence, cognitive participation, collective action, and reflexive monitoring* | - Can you think of any challenges to delivering HELP to all carers across the UK?   - Can you think of anything we can do to help overcome this?   - How can we best encourage carers to take part in HELP?   - How can we effectively tell them about the programme?  - Can you think of anything that might stop carers taking part?  - How may we overcome this? |
| *Interview Close* | - Could you summarise your opinion of HELP in a few words? - Is there anything else you would like to discuss? |

CFIR = Consolidated Framework for Implementation Research; NPT = Normalisation Process Theory; and HELP = HEart faiLure carer support Programme.

***Topic Guide for Interviews with Healthcare Professionals***

| **CFIR Domains and NPT Constructs** | **Semi-Structured Interview Question** |
| --- | --- |
| ***CFIR:*** *Intervention Characteristics*  ***NPT:*** *Coherence, Cognitive Participation, Collective action, and Reflexive Monitoring* | - Can you talk about your overall opinion of HELP?   - Which elements do you like / dislike?  - What do you think of the educational booklet and HELP website?  - What are your thoughts on the online support group sessions?   - What are your thoughts on the online parts of HELP?   - Do you think this is suitable for carers?   - Is there anything in general we should change about the programme? - How does HELP compare to other programmes of support for carers you are aware of?   - What are the advantages / disadvantages comparatively?   - Would any changes to the programme be needed to make it work at [insert site]?   - How well would HELP be received at [insert site]? |
| ***CFIR****: Outer Setting*  ***NPT:*** *Coherence and Reflexive Monitoring* | - Do you think there is a need for carers to receive HELP?   - How well would the programme meet these needs and how?  - Is there a local need at [insert site]?   - How do you think HELP would impact carers and patients? |
| ***CFIR:*** *Inner Setting*  ***NPT:*** *Coherence, Cognitive Participation, Collective action, and Reflexive Monitoring* | - What support is available at [insert site] to aid HELP adoption?   - Are there any local incentives for making HELP implementation successful?  - Are there any local barriers to HELP implementation? If so, can you think of ways to overcome these?   - To what extent does [insert site] set goals for supporting carers of patients with heart failure? - How do you think the culture at [insert site] would affect the implementation of HELP? |
| ***CFIR:*** *Characteristics of Individuals*  ***NPT:*** *Coherence, Cognitive Participation, Collective action, and Reflexive Monitoring* | - What is your opinion of supporting HELP implementation? - How confident would you be in HELP being routinely delivered at [insert site]?   - How confident do you think your colleagues would be?  - How can we best support this? |
| ***CFIR:*** *Implementation Process*  ***NPT:*** *Coherence, Cognitive Participation, Collective action, and Reflexive Monitoring* | - Can you think of any challenges to routinely delivering HELP across the UK?   - Can you think of anything we can do to help overcome this?   - Who would you identify as key stakeholders for supporting HELP adoption?   - How could we encourage their involvement?   - How would HELP fit within the workflow at [insert site]?   - Can you think of ways of supporting HELP to fit? |
| *Interview Close* | - Could you summarise your opinion of HELP in a few words? - Is there anything else you would like to discuss? |

CFIR = Consolidated Framework for Implementation Research; NPT = Normalisation Process Theory; and HELP = HEart faiLure carer support Programme.

***Topic Guide for Interviews with HELP Delivery Staff***

| **CFIR Domains and NPT constructs** | **Semi-Structured Interview Question** |
| --- | --- |
| ***CFIR:*** *Intervention Characteristics*  ***NPT:*** *Coherence, Cognitive Participation, Collective action, and Reflexive Monitoring* | - Can you talk about your overall opinion of HELP?   - Which elements do you like / dislike?  - What do you think of the educational booklet and HELP website?  - What are your thoughts on the online support group sessions?   - What are your thoughts on the online parts of HELP?   - Do you think this is suitable for carers?   - Is there anything in general we should change about the programme? - How does HELP compare to other programmes of support for carers you are aware of?   - What are the advantages / disadvantages comparatively?   - Did you have to make any changes to the programme to make it work at [insert site]?   - How well was HELP received at [insert site]? |
| ***CFIR****: Outer Setting*  ***NPT:*** *Coherence and Reflexive Monitoring* | - Do you think there is a need for carers to receive HELP?   - How well does the programme meet these needs and how?  - Is there a local need at [insert site]?   - How do you think HELP impacts carers and patients? |
| ***CFIR:*** *Inner Setting*  ***NPT:*** *Coherence, Cognitive Participation, Collective action, and Reflexive Monitoring* | - What support is available at [insert site] to aid HELP adoption?   - Are there any local incentives for making HELP implementation successful?  - Are there any local barriers to HELP implementation? If so, can you think of ways to overcome these?   - To what extent does [insert site] set goals for supporting carers of patients with heart failure? - How do you think the culture at [insert site] would affect the implementation of HELP? |
| ***CFIR:*** *Characteristics of Individuals*  ***NPT:*** *Coherence, Cognitive Participation, Collective action, and Reflexive Monitoring* | - What has been your motivation for wanting to support HELP implementation? - How confident were you about HELP delivery?   - How did you find the training before formal implementation?   - What skills does a nurse need to effectively deliver this programme?   - How can we best support this? |
| ***CFIR:*** *Implementation Process*  ***NPT:*** *Coherence, Cognitive Participation, Collective action, and Reflexive Monitoring* | - Did you experience any challenges with delivering HELP?   - Can you think of anything we can do to help overcome this?   - Who would you identify as key stakeholders for supporting HELP adoption?   - How could we encourage their involvement?   - How would HELP fit within the workflow at [insert site]?   - Can you think of ways of supporting HELP to fit? |
| *Interview Close* | - Could you summarise your opinion of HELP in a few words? - Is there anything else you would like to discuss? |

CFIR = Consolidated Framework for Implementation Research; NPT = Normalisation Process Theory; and HELP = HEart faiLure carer support Programme.
