# Supplementary material for: Evaluating the implementation and impact of the HEart faiLure carer support Programme (HELP) in the United Kingdom: A study protocol for a multi-centre, mixed-method, implementation study
Source: PLoS One. 2026 Apr 17;21(4):e0347037. doi: 10.1371/journal.pone.0347037 (PMC13089873; doi:10.1371/journal.pone.0347037)
Supplement: S6 Text — (DOCX) [file pone.0347037.s006.docx]

**Implementation Testing of the** **HEart faiLure carer support Programme (HELP) in the United Kingdom**

***Study Protocol***

# **Project Details**

## **Study Title**

Implementation Testing of the HEart faiLure carer support Programme (HELP) in the United Kingdom.

## **Funder**

**Name:** British Heart Foundation

**Programme:** Healthcare Innovation Fund

**Reference:** HI/C2/24/360034

**Contact:** Dr Iain Armstrong, Senior Programme Manager, British Heart Foundation,

Greater London House, 180 Hampstead Road, London, NW1 7AW

**Email:** [armstrongi@bhf.org.uk](mailto:armstrongi@bhf.org.uk)

## **Chief Investigator**

**Name:** Professor Donna Fitzsimons

**Post:** Professor

**Address:** Room No. 04.328, School of Nursing and Midwifery, Queen’s University Belfast, Medical Biology Centre, 97 Lisburn Road, Belfast, BT9 7BL

**Email:** [d.fitzsimons@qub.ac.uk](mailto:d.fitzsimons@qub.ac.uk)

## **Project Team**

1. Professor Judy Bradley (Alternative Chief Investigator), Director of Clinical Research Facility, School of Medicine, Dentistry, and Biomedical Sciences, Queen’s University Belfast
2. Dr Gareth Thompson (Research Fellow / Project Manager), School of Nursing and Midwifery, Queen’s University Belfast
3. Professor Loreena Hill (Professor of Nursing Research and Impact), School of Nursing and Paramedic Science, Ulster University
4. Professor Martin Dempster (Professor), School of Psychology, Queen’s University Belfast
5. Dr Patrick Stark (Expert Advisor - Statistics), Lecturer, School of Nursing and Midwifery, Queen’s University Belfast
6. Professor Mike Clarke (Expert Advisor - Research Methodology), Director of MRC Methodology Hub, School of Medicine, Dentistry, and Biomedical Sciences, Queen’s University Belfast
7. Dr Nicola Johnston (Clinical Contact), Consultant Cardiologist, Royal Victoria Hospital, Belfast Health and Social Care Trust
8. Dr Lana Dixon (Clinical Contact), Consultant Cardiologist, Royal Victoria Hospital, Belfast Health and Social Care Trust
9. Dr Patricia Campbell (Clinical Contact), Consultant Cardiologist, Craigavon Area Hospital, Southern Health and Social Care Trust
10. Dr Patrick Donnelly (Clinical Contact), Consultant Cardiologist, Ulster Hospital, South Eastern Health and Social Care Trust
11. Professor Theresa McDonagh (Clinical Contact), Consultant Cardiologist, King’s College Hospital, NHS Foundation Trust
12. Dr Susan Piper (Clinical Contact), Consultant Cardiologist, King’s College Hospital, NHS Foundation Trust
13. Mrs Yvonne Millerick (Clinical Contact), Nurse Consultant, Glasgow Royal Infirmary

## **Collaborator**

**Name:** United Kingdom Clinical Research Network

**Lead Contact:** Dr Paul Biagioni

**Email:** [paul.biagioni@nicrn.hscni.net](mailto:paul.biagioni@nicrn.hscni.net)

## **Project Contact**

**Name:** Dr Gareth Thompson (Research Fellow / Project Manager)

**Address:** School of Nursing and Midwifery, Queen’s University Belfast, Medical Biology Centre, 97 Lisburn Road, Belfast, BT9 7BL

**Email:** [gareth.thompson@qub.ac.uk](mailto:gareth.thompson@qub.ac.uk)

**Sponsor**

**Name:** Queen’s University Belfast

**Contact Person:** Dr Paula Tighe

**Address:** Research Governance, Ethics, and Integrity, Queen’s University Belfast, Belfast, BT7 1NN

**Email:** [p.tighe@qub.ac.uk](mailto:p.tighe@qub.ac.uk)

## **Study Management**

Abbreviations: DF = Donna Fitzsimons, JB = Judy Bradley, GT = Gareth Thompson, LH = Loreena Hill, MD = Martin Dempster, SHNs = Specialist Heart Failure Nurses, PS = Patrick Stark, MC = Mike Clarke, NJ = Nicola Johnston, LD = Lana Dixon, PC = Patricia Campbell, PD = Patrick Donnelly, TMcD = Theresa McDonagh, SP = Susan Piper, and YM = Yvonne Millerick.

- DF is the Chief Investigator and is an experienced cardiovascular nurse and researcher with expertise in HF. DF will provide oversight and be primarily responsible for project delivery (alternative Chief Investigator to deputise when required). DF can assume the responsibility of the Project Manager or HELP delivery staff (SHNs) in emergency circumstances.
- JB is the alternative Chief Investigator and has expertise with process evaluation and clinical trial management. JB can assume the responsibility of Chief Investigator if required with no negative impact to the study. Her role is also to provide additional expert oversight.
- GT has expertise with clinical trial management and delivery in cardiovascular populations. GT is the Project Manager who will conduct the daily running of the project, such as: training staff for potential participant identification, recruiting participants, data collection, administrative tasks, analysis, write-up, and dissemination of findings.
- LH is an experienced cardiovascular nurse with expertise in HF. LH will provide expert academic and clinical oversight and can assume the responsibility of the Project Manager or HELP delivery staff (SHNs) in emergency circumstances.
- MD is a psychologist and has expertise with clinical research methodology and psychological evaluations. MD will provide expert methodological and intervention oversight.
- SHNs (identities confirmed when start date finalised) will be responsible for HELP delivery and completion of relevant data collection items (*i.e.,* intervention delivery checklists and logs). SHNs will also maintain regular contact with the Project Manager and wider team to provide updates on intervention delivery.
- PS and MC are statistical and methodological experts, respectively, who will provide guidance and advice over the course of the project.
- NJ, LD, PC, PD, TMcD, SP, and YM are clinicians and will be the clinical leads across their respective sites. They will be responsible for overseeing potential participant identification and communicating with the Project Manager to support the recruitment strategy. They will also raise the project profile across their respective sites and provide expert clinical input over the project lifetime.

### **Contingency Plan**

The project team consist of experienced researchers with a track record of successful, large, multi-centre studies. We enjoy strong clinical and professional relationships with the collaborating centres, which will streamline project set-up and completion of early milestones. Project management will be overseen by an experienced Steering Group comprising important stakeholders (*i.e.,* academics, PPI members, and healthcare professionals), which will ensure diligent monitoring of progress towards project milestones, with early intervention should any problems arise. All members of the project team have full understanding of the project and associated requirements. DF and LH are trained in all aspects of project / intervention delivery should the project manager or HEart faiLure carer support Programme (HELP) delivery staff be unavailable in emergency circumstances. The analysis and writing of results can be transferred within the team, if necessary.

##### **Steering Group**

A Steering Group has been established for the monitoring and governance of HELP implementation. This group will be co-led by academics (Professor Donna Fitzsimons and Professor Loreena Hill) and a PPI member (Bernie Taylor, carer of a patient with HF) who was involved with the original co-design and refinement of HELP. The group will also comprise other key stakeholders involved with intervention development (patients, carers, healthcare professionals, and academics), carer champions (those who have received the intervention), staff responsible for routine delivery of HELP, and representatives from patient / carer support organisations (BHF and HF Warriors Northern Ireland). A range of protected characteristics (*i.e.,* age, socio-economic status, and ethnicity) will be present across the members to ensure equality and diversity. The Steering Group will meet virtually every 3 months over the project lifetime (2 years) to receive input from delivery staff on the performance of HELP in routine practice, which will enable critical reflection and identification of any required amendments (*i.e.,* context-specific tailoring). This Group will also review participant-facing materials, guide dissemination, and provide input on any project-related issues (*i.e.,* recruitment and retention). Following consensus agreement amongst the Steering Group, any formal change requests will be submitted to the BHF and Ethics / Governance Committees. Communication between meetings will occur as required.

##### **Intervention Oversight Group**

An Intervention Oversight Group co-chaired by Professor Mike Clark and Professor Martin Dempster will provide an arm’s length assessment of programme fidelity and oversee notes from weekly team meetings across all sites. This group will meet monthly and offer question and answer support to Dr Gareth Thompson (Project Manager) and all site personnel. Communication between meetings will occur as required.

##### **Operational Project Team**

The Operational Project Team (DF, JB, GT, and LH) will cooperate to provide the Project Manager (GT) with oversight and guidance for daily management and project delivery. Monthly meetings will be held and communication between meetings will occur as required.

##### **General Project Team**

The General Project Team (listed in ‘Project Details’ section) will oversee project management / delivery, evaluation methodology, and implementation procedures. Meetings will be held every two months and communication between meetings will occur as required.

#### **UK Clinical Research Network Adoption**

The project will seek adoption by the UK Clinical Research Network (UKCRN) across the collaborating regions (Northern Ireland, England, and Scotland), which will provide support with recruitment and extraction of relevant outcome data from medical records (discussed below).

# **Abstract**

## **Background**

Rates of heart failure (HF) are rising dramatically in the United Kingdom (UK) and worldwide. Many of these patients rely on support from family carers who are ill-prepared and supported for this role. Additionally, these carers often experience poor mental and physical health, which has been linked to their caring role and the health status of the patient they care for. To address these issues, the HEart faiLure carer support Programme (HELP) was co-designed with carers, healthcare professionals, and academics in the UK. Pilot testing of HELP demonstrated feasibility and acceptability, with evidence of a beneficial effect on preparedness and emotional well-being for carers of patients with HF. In line with UK Medical Research Council guidance, this project will advance HELP to implementation testing in a real-world environment.

## **Aim**

To assess the real-world implementation of HELP for carers of patient with HF across the UK.

## **Objectives**

1. Investigate the facilitators and barriers to HELP implementation.
2. Assess real-world patient and carer-related outcomes.
3. Estimate the economic cost of delivering HELP in the UK.

## **Methods**

The project design will be a mixed-method, multi-centre prospective cohort study, with nested process and economic evaluations. Specialist Heart Failure Nurses will be trained by the project team to deliver HELP across 3 UK-based sites (Northern Ireland, England, and Scotland) to approximately 180 carers of patients with HF. The carers targeted will be those who require additional support (caring for a patient with symptomatic HF). These carers will be recruited via patients identified by local cardiology teams at in- and out-patient appointments. HELP includes an educational booklet and supplementary website, along with 6 online educational support sessions delivered weekly. Data will be collected on the following variables: 1) Implementation fidelity (*e.g.,* what was implemented & how closely this reflected what was intended), 2) Contextual factors (*e.g.,* barriers to and facilitators of implementation), 3) Carer and patient-related outcomes (*e.g.,* hospital admissions, GP visits, quality of life, carer burden, and carer preparedness), and 4) Cost of HELP implementation. This data will be collected from carers, patients, and staff through questionnaires, checklists / logs, medical records, and interviews across 4 time points: 1) Baseline, 2) Intra-HELP, 3) Post-HELP, and 4) 6-month follow-up. Quantitative data will be reported with descriptive statistics and mean difference (95% Confidence Interval). Qualitative data will be analysed using Framework analysis. An integrative analysis will be conducted to identify synergies between the quantitative and qualitative data.

## **Potential Impact**

The information generated will outline the capacity and requirements for regional implementation of HELP. These findings will be shared with policy makers and commissioners to inform the large-scale, roll-out and embedding of HELP in routine clinical practice, which addresses a critical area of unmet need for carers of patients with HF.

# **Study Protocol**

## **Background**

The worldwide prevalence rate for heart failure (HF) is approximately 64.3 million, with this number increasing significantly over the previous twenty-eight years secondary to an aging population (1). Effective self-management of symptoms by patients is recognised as a key strategy for reducing hospital readmissions and increasing patient quality of life (QoL) (2). However, as HF progresses, self-management of symptom burden becomes increasingly challenging, which results in patients relying on support from family carers (2). Previous research has comprehensively reported that carers experience diminished mental and physical QoL, which correlates with their caring role and the health status of the patient they support (3,4).

The vital role fulfilled by carers in supporting patients with self-management is reflected in the European Society of Cardiology guidelines for HF treatment (2). However, the literature documents that carers require improved support and information (3–7). A systematic review and meta-analysis conducted by our team demonstrated a scarcity of interventions designed to provide psychological and educational support to carers of patients with HF, with the few studies available showing insignificant results and poor reporting of the intervention development process (8). Moreover, there are no supportive interventions co-designed with carers to exclusively target their requirements within the United Kingdom (UK) (8).

### **HEart faiLure carer support Programme (HELP) Development and Feasibility Testing**

To address the significant area of unmet need, members of the project team systematically developed ‘The HEart faiLure carer support Programme (HELP)’ according to the UK Medical Research Council (MRC) framework for developing and evaluating complex interventions (9). Prior research conducted by the team identified the need for this intervention and highlighted required components (3,4):

1. Peer-support group supplemented by an educational booklet: Provide carers with psychological support and educational information on symptom management, self-care, and support services available
2. Carers prioritised: Healthcare professionals must recognise the role of carers and signpost them to support available
3. Support offered early in HF trajectory: Patient diagnosis should trigger initiating support and information for carers

The design of the support group was informed by a systematic review and meta-analysis performed by the team (8), which evaluated what components from previous interventions were the most successful at improving carer outcomes. It was concluded that 4-6 online support group sessions (approximately 60 minutes each) should be delivered, with the following educational topics covered:

1. Carer’s wellbeing
2. Communication and support
3. Understanding HF
4. Practical Skills for self-management

The development of the educational booklet was underpinned by scoping the written and online resources available to carers of patients with HF across the UK (*i.e.,* from key charities such as the British Heart Foundation (BHF), Irish Heart Foundation, Northern Ireland Chest Heart and Stroke, and Heart Failure Matters), along with an evaluation of the educational resources used by previous studies (8). The most common educational topics were identified, and a draft educational booklet was developed by the project team, which includes direct quotations from carers who participated in previous research (4) and the following five chapters:

1. What is HF?
2. Planning for the future
3. Looking after yourself
4. Communication
5. Your role as a carer

An interdisciplinary stakeholder group was established at the beginning of HELP development. This group comprised a consultant cardiologist (n = 1), specialist HF nurses (n = 3), carers of patients with HF (n = 1), representation from a voluntary organisation for carers (n = 1), and members of the wider project team with previous caring experience (n = 4). This group was directly involved with informing and refining the design of the intervention components, which ensured co-design was a central feature throughout the developmental process.

Following HELP development, a convergent mixed-method study was conducted to evaluate the feasibility and acceptability of the intervention in Northern Ireland. Participants received HELP over 6 weeks, which involved an educational booklet and 6 support group sessions delivered via ZOOM by the research team (healthcare professionals and academics). Quantitative measures (pre-post, validated questionnaires and feedback questionnaires) assessed recruitment and attrition rates, intervention completion, and usefulness of the intervention. Qualitative measures (focus groups) explored the acceptability of the intervention. In total, 51 participants were eligible, and 22 (43%) consented to take part. Of those 22 participants, 18 (89%) completed the intervention, representing favourable adherence and low attrition. Participants had a mean age of 58 years, and the majority were female (n = 16, 89%), married (61%, n = 11), were either spouses or adult children, and their education ranged from no qualifications to a higher degree. Participants highly rated the usefulness of the intervention, with the integration of quantitative and qualitative data generating 3 key findings:

1. Improved carer preparedness
2. Strong support from group environment
3. Improved emotional wellbeing

This study demonstrated that delivering HELP to carers of patients with HF is feasible and has the potential to improve carer preparedness, emotional wellbeing, and make them feel empowered in their caring role. These improvements for carers may have a positive impact on enhancing patient health (*i.e.,* reduced hospital readmissions and improved QoL) as carers are able to provide more effective support and assist with self-care activities more confidently (2).

### **Rationale**

HELP is the first co-designed service for carers of patients with HF across the UK, which addresses an important area of unmet need. Whilst our pilot work demonstrated feasibility, acceptability, and a positive impact on carers, the results do not inform policy-makers or service commissioners what factors influence successful implementation and whether HELP would generate the same outcomes in the ‘real-world’ setting. In line with MRC guidance (10), funding has been secured from the BHF to conduct a comprehensive process evaluation of HELP implementation in real-world clinical settings (delivery by local healthcare professionals) across the UK. Process evaluation methodology is an established method of understanding implementation by investigating (11):

1. Fidelity (the degree to which the intervention was delivered as intended)
2. Context (barriers to and facilitators of implementation)
3. Potential mechanisms of impact

As the potential mechanisms of impact were explored in the pilot phase (12), this stage of the project will focus on fidelity and context in the real-world setting. This will generate an understanding of the requirements for successful HELP implementation, how adaptations or site differences influence outcomes, and identify any necessary alterations. These findings will inform large-scale, national implementation of a novel intervention that provides essential psychoeducational support to the carers of patients with HF across the UK.

## **Aim**

To conduct process and economic evaluations of HELP implementation for carers of patients with HF across real-world, clinical settings in the UK.

### **Objectives**

1. Identify the facilitators and barriers to HELP implementation
2. Evaluate patient and carer outcomes
3. Estimate the economic cost of HELP implementation in the UK

## **Methods**

### **Study Design and Frameworks**

A mixed-method, implementation study adopting a multi-centre prospective cohort study design, with nested process and economic evaluations will be conducted. These assessments will be guided by the integration of the Consolidated Framework for Implementation Research (CFIR) and Normalisation Process Theory (NPT) (13), which provides a holistic insight into the macro (*e.g.,* system-level) and micro (*e.g.,* individual-level) factors that contribute to and enhance implementation outcomes (14). The study will follow the Standards for Reporting Implementation Studies (StaRi) (15).

The protocol is based on four key components, which contribute to completing the objectives:

1) Fidelity of implementation: what was implemented and how closely this reflected what was intended (*i.e.,* the original HELP intervention) (Objective 1)

2) Contextual factors: barriers to and facilitators of implementation, as perceived by the carers, healthcare professionals, and delivery staff involved (Objective 1)

3) Patient & carer-related outcomes: whether, and to what extent, improvements in patient and carer outcomes are evident and what is the long-term adherence (Objective 2)

4) Economic cost: implementation costs for HELP delivery across the UK (Objective 3)

A study flow diagram is presented in Figure 1.

**Figure 1. Study Flow Diagram.**


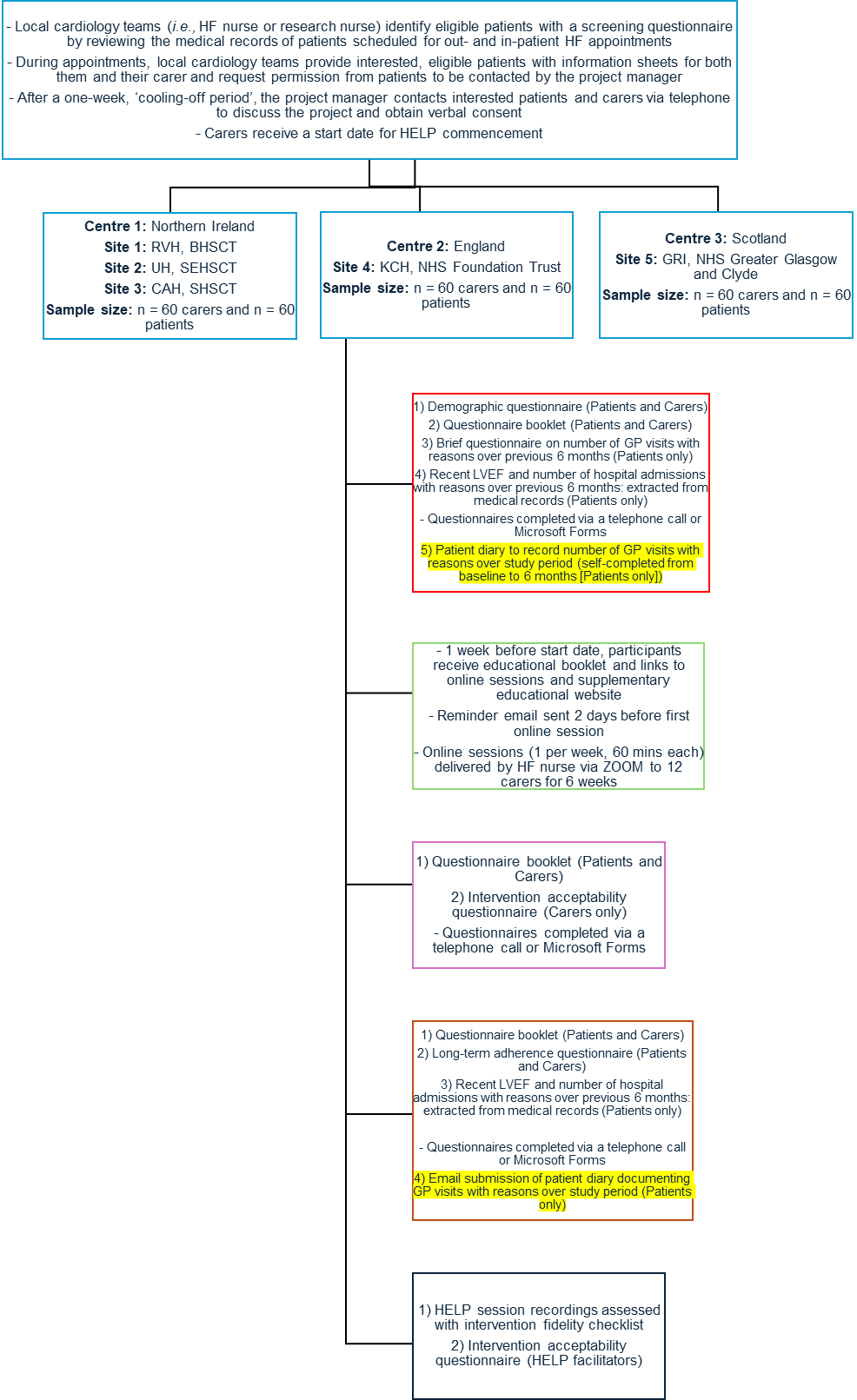


1) Intervention deviations recorded in implementation log

2) Facilitator log completed after each session

3) Delivery costs and time input recorded in implementation and facilitator logs

4) Weekly HELP impact questionnaire (Patients and Carers)

1) HELP facilitator competency assessed with checklist

2) Training costs recorded in implementation log

- Cases of carers and patients who agreed to a possible interview will be purposively selected and contacted

- Interested cases receive information sheets via email

- Following a 1-week, ‘cooling-off’ period, the project manager will contact patients and carers to receive verbal consent and arrange a time for a telephone interview

- Telephone interviews conducted with n = 10 carers and n = 10 patients

- Clinical staff from the project team supply interested colleagues (HF consultants and HF nurses) with information sheets for interviews

- After a 1-week, ‘cooling-off’ period, interested potential participants will contact the project manager to provide verbal consent and arrange a time for a telephone interview

- Participants provide demographic details via Microsoft Forms

- Telephone interviews conducted with n = 3 HELP facilitators, n = 5 HF nurses, and n = 5 HF consultants

**Baseline**

**Post-Intervention (6 weeks)**

**6 Months Post-Intervention**

**Study End**

**Participant Identification & Recruitment**

**HELP Delivery**

- 5 rollouts of HELP (6 weeks per run) at each centre over a 12-month recruitment and delivery period

- 12 carers recruited per run (60 carers per centre; 180 carers in total)

- Programme runs separated by 4-week recruitment windows

**Legend:** UK = United Kingdom; HELP = HEart faiLure carer support Programme; RVH = Royal Victoria Hospital; BHSCT = Belfast Health and Social Care Trust; UH = Ulster Hospital; SEHSCT = South Eastern Health and Social Care Trust; CAH = Craigavon Area Hospital; SHSCT = Southern Health and Social Care Trust; KCH = King’s College Hospital; NHS = National Health Service; GRI = Glasgow Royal Infirmary; GP = General Practitioner; HF = heart failure, and LVEF = left ventricular ejection fraction.

#### **Setting**

HELP will be delivered virtually by local, HF nurses across 3 centres (5 sites) in the UK:

**Centre 1: Northern Ireland**

**Site 1:** Royal Victoria Hospital, Belfast Health and Social Care Trust

**Site 2:** Ulster Hospital, South Eastern Health and Social Care Trust

**Site 3:** Craigavon Area Hospital, Southern Health and Social Care Trust

**Centre 2: England**

**Site 4:** King's College Hospital, NHS Foundation Trust

**Centre 3: Scotland**

**Site 5:** Glasgow Royal Infirmary, NHS Greater Glasgow and Clyde

These sites will constitute early adopters of HELP and will be designated as ‘Beacon Sites’. The Beacon Sites will be used to model programme implementation, which will inform and support routinisation / embedding of innovative practice (16). Sites were selected due to their ability to commit to HELP implementation, to diversify sample inclusion, and increase geographic spread. Moreover, the project team have strong existing relationships with the clinical and support staff at the collaborating sites, which will maximise the likelihood of fidelity, co-ordination, and seamless roll out.

#### **Sample and Recruitment**

#### **Carers**

The target population is family carers of people with symptomatic HF across the three collaborating centres (Northern Ireland, England, and Scotland).

##### **Inclusion Criteria**

1) Aged 18 years or older

2) Physical and mental ability to operate a digital device, engage comfortably in conversation with peers, and be able to read written materials

3) Caring (regular, unpaid care) for a person with a clinical diagnosis of HF who is receiving standard evidence-based HF therapies and has either HF symptoms or 1 episode of decompensation (symptoms requiring medical attention) in previous 6 months

4) Ability to speak and understand English

##### **Exclusion criteria**

1) Lacking capacity to give consent

2) Under the age of 18

This population successfully participated in the pilot study and will be targeted as they require additional support (caring for a patient with symptomatic HF). This will also standardise the characteristics of the programme recipients. Those caring for patients who are asymptomatic are less likely to benefit from HELP.

##### **Sample Size**

We will have 5 programme runs of HELP (6 weeks per run) across each of the 3 centres over a 12-month period, with 12 carers per run (60 carers per centre; 180 carers in total). To inform this recruitment timeframe, we scoped participant identification across in- and out-patient appointments at a collaborating site (Royal Victoria Hospital). 15 patients with symptomatic HF were identified in one week, with 13 (90%) of whom having an informal carer. Generalising this across the 3 centres results in approximately 156 eligible carers per month and 1,872 over the 12-month recruitment period. To achieve our required sample size (180 carers), we need to recruit < 10% of the total sample population, which is highly likely given the recruitment rate (43%) demonstrated in the pilot study (12). Moreover, our other research across the collaborating sites has delivered 75-100% recruitment rates (17). According to pilot data (12), a minimum of 67 participants is required to detect a large effect size for change in carer preparedness scores at baseline to follow-up (g = 0.8), with an alpha value of 0.05 and statistical power of 90%.

#### **Patients**

The patients who are receiving support from the recruited carers will be invited to provide outcome data, which will enable an assessment of the dyadic impact of HELP.

**Inclusion Criteria**

1) Aged 18 years or older

2) Physical and mental ability to engage comfortably in conversation and be able to read written materials

3) Clinical diagnosis of HF and receiving standard evidence-based HF therapies and has either HF symptoms or 1 episode of decompensation (symptoms requiring medical attention) in previous 6 months

4) Ability to speak and understand English

5) Receiving regular, unpaid support with managing his / her condition from a loved one or friend (e.g., a relative or spouse)

**Exclusion criteria**

1) Lacking capacity to give consent

2) Under the age of 18

3) Carer refused to participate in HELP

**Sample Size**

One patient per carer will be invited to enhance sample diversity (180 patients in total, maximum). Carers will remain eligible for participation regardless of patient enrolment.

**Carer and Patient Recruitment**

This project will comply with all regulatory requirements. Prior to initiation, ethical and governance approvals will be sought from QUB, NHS / HSC Research Ethics Committee (REC), and local NHS / HSC R&D offices using the Integrated Research Application System (IRAS). There will be 5 rollouts of HELP (6 weeks per run) at each of the 3 centres over a 12-month recruitment and delivery period (approx. 52 weeks). Approximately 12 carers will be recruited per run (60 carers per centre; 180 carers in total). Programme runs will be separated by 4-week windows (see Table 1), which will account for standard holidays, sickness, staff shortages, and provide adequate intervals between programme runs for recruitment (*i.e.,* filling group numbers).

**Table 1. HELP Delivery and Recruitment Schedule for each Centre.**

| **Weeks** | **1-4** | **5-10** | **11-14** | **15-20** | **21-24** | **25-30** | **31-34** | **35-40** | **41-44** | **45-50** |
| --- | --- | --- | --- | --- | --- | --- | --- | --- | --- | --- |
| ***HELP Delivery*** |  | **Run 1** |  | **Run 2** |  | **Run 3** |  | **Run 4** |  | **Run 5** |
| ***Recruitment*** |  |  |  |  |  |  |  |  |  |  |

To identify eligible patients, local cardiology teams (*i.e.,* HF nurse or research nurse) across the sites will use a screening questionnaire (see Appendix 1) to review the medical records of patients scheduled for out- and in-patient HF appointments. These staff will have the option of contacting eligible patients via phone call to confirm their willingness to be approached during scheduled appointments to discuss the study. If this contact is not possible due to time constraints, staff will approach eligible patients during appointments for first contact. During appointments, local cardiology teams will discuss the project and provide an information pack containing an invitation to participate and information sheets for both them and their carer (see Appendices 2 & 3). Patients will be asked to pass the carer information sheet to a loved one (*e.g.,* a relative or spouse) who regularly provides unpaid, support with managing their condition. If an appointment is virtual / telephone-based, local cardiology teams will request permission from patients to send the information packs via email (see Appendices 2 & 3). As HELP targets carers, patients will be informed by staff and the information packs that their participation is dependent on their carer also enrolling in the project, and also that carers may participate regardless of the patients’ decision. Interested patients will be asked to provide local cardiology staff with permission to pass their contact details to the project manager (GT) via encrypted email. After a one-week, ‘cooling-off period’, the project manager (GT) will contact interested patients via telephone to discuss the project and obtain verbal consent (see Appendix 4). During this call, patients will also provide the project manager (GT) with the contact details of their carer. Subsequently, the project manager (GT) will contact carers via telephone to confirm eligibility with a screening questionnaire (see Appendix 5) before discussing the project and obtaining verbal consent (see Appendix 6). Recruitment across the collaborating sites will ensure equality and diversity across protected characteristics (*i.e.,* education, socio-economic status, ethnicity, and pre-existing digital capability); sites in Northern Ireland cover areas rated from low to high on the social deprivation index and King’s College Hospital serves an ethnically diverse population (*i.e.,* black and Asian).

#### **Intervention Design**

The HELP intervention is a 6-week, psychoeducational programme consisting of two components (see Figure 2):

1. Online support group sessions

6 online sessions underpinned by standardised presentations, which are delivered over 6 weeks (1 session per week, 60 minutes each) and cover the topics displayed in Table 2.

**Table 2. Support Group Schedule.**

| Sessions | Content |
| --- | --- |
| Session 1: Introduction | - Group introductions - Icebreaker activity *(Share one interesting fact about yourself)* - Ground rules - Reminder to read Chapters 1 & 2 of educational booklet in preparation for Session 2 - Introduction to the supplementary website and guidance on access and usage |
| Session 2: Understanding HF | - Overview of the HF condition - Symptoms - Prognosis - Palliative care - Reminder to read Chapter 3 of educational booklet in preparation for Session 3 |
| Session 3: Personal wellbeing | - Importance of looking after your physical and emotional wellbeing - Coping strategies on how to deal with difficult emotions - Tips on how to look after yourself - Reminder to read Chapter 4 of educational booklet in preparation for Session 4 |
| Session 4: Communication and support | - Importance of communicating with your loved ones - Tips on how to communicate with healthcare professionals - Reminder to read Chapter 5 of educational booklet and bring medication list along to Session 5 |
| Session 5: Practical skills for self-management | - Importance of diet and controlling salt intake - Managing and keeping track of fluid retention - Overview of medications and how to manage them - Exercise - Tips on how to monitor symptoms at home |
| Session 6: Feedback session | - Discussion of experience and final comments / questions - Feedback on intervention components (content, delivery, and format) - Suggestions on how to improve the intervention |

Legend: HF = heart failure.

1. Educational booklet and supplementary website

The educational booklet is a co-designed, professionally produced, written document (see Appendix 7) that complements the support group sessions by facilitating self-directed learning across the following 5 chapters:

Chapter 1: What is HF?

Chapter 2: Planning for the future

Chapter 3: Looking after yourself

Chapter 4: Communication

Chapter 5: Your role as a carer

Each chapter is completed over the week leading to the corresponding support group session. To improve potential for scalability, the project team collaborated with Focus Games Ltd (GDPR registered and compliant) to produce a digitalised version (website) of the educational booklet to improve accessibility (*i.e.,* remote access on mobile phones / tablets), with PPI input from patients and carers underpinning the developmental process. In addition to the educational booklet, carers will receive access to this supplementary website (accessible via computers and mobile devices), which provides the option to receive the same information from the educational booklet in a potentially more engaging and interactive format (*i.e.,* animations, interactive content, and topic summaries). The website also provides access to the standardised presentations for the online support group sessions, which will enable carers to preview and study ahead of the live sessions. If interested, carers will have the option to submit questions via the website for the facilitator to answer at the next online support group session. Importantly, the website serves as an optional supplement to the educational booklet. For review purposes, the link to the website is provided in the cover letter.

The Transactional Theory of Stress and Coping (18) guided the selection of intervention components, outcomes, and expected mechanisms of impact. This theory proposes that when individuals appraise a situation as exceeding their resources, their wellbeing is compromised, which is consistent with evidence demonstrating how carers experience significant unmet needs, burden, and stress (4). It is also commonly used in the development of evidence-based interventions for carers (19). HELP will facilitate improved access to information and support from peers and healthcare professionals, which would act as key practical and emotional coping resources to alleviate stress, burden, and improve carer wellbeing, and ultimately empower carers in their caregiving role. The intervention components are mapped to the Theoretical Domains Framework, which facilitates theoretical grounding of the mechanisms of impact for behavior change (20). The applicability of the chosen theory and mechanisms of impact will be assessed throughout the data analysis process, with consideration given to whether the results are reflective of the theoretical underpinnings.

The NPT framework (13) was used to ensure implementation strategies were theoretically grounded across the following constructs: 1) coherence (*i.e.,* does the intervention make sense?), 2) cognitive participation (how do people engage?), 3) collective action (how do people work with the intervention?) and 4) reflexive monitoring (how do people appraise the intervention?). Implementation strategies mapped to the NPT framework were developed with oversight from the Steering Group (see Table 3). Moreover, with input from the Steering Group, we have developed a two-day online or in-person training programme to train staff responsible for HELP delivery, which covers essential skills for intervention implementation (*i.e.,* effective communication, building trusting relationships, managing conflict, delivering behaviour change techniques, and familiarisation with educational content). The training will be delivered by the project team and utilise an interactive approach, including content talks, case scenario discussions, role plays, recordings, and input from carers on their needs and requirements. Trained staff will receive access to the intervention content and standardised materials to support HELP delivery. Regular support will be provided by the project team, with frequent communication between the delivery staff and Project Manager for monitoring and prompt resolution of issues as required.

**Table 3. Implementation Strategies Mapped to the NPT Framework (13).**

| **NPT Construct** | **Implementation Strategies by Implementation Agent** | | | |
| --- | --- | --- | --- | --- |
|  | **HELP Facilitator (Specialist HF Nurse)** | **Referral Staff (Local Cardiology Teams)** | **Clinical Management (PIs across collaborating sites)** | **Carers** |
| **1. Coherence** (Does it makes sense?) | - Training  - Information from project documents  - Fidelity checklist | - Information from project documents  - Communication with Project Manager | - Members of the Steering Group  - Regular discussion with project team | - Information from project documents and intervention sessions  - Support from Project Manager |
| **2. Cognitive Participation**  (How do people engage?) | - Motivation and commitment established during training  - Documents and support available to facilitate delivery | - Project documents and Project Manager provide guidance  - Screening log to be completed | - Discussion with project team on potential importance of the intervention and guidance on what is required | - Encouragement from HELP Facilitators  - Attendance at online support group sessions  - Educational booklet and supplementary website to support  - Tailored support provided at online support group sessions |
| **3. Collective Action**  (How do people work with intervention?) | - Receive appropriate training  - Support from project team  - Fidelity checklist | - Actively referring participants  - Support from Project Manager | - Management displays support in meetings and encourages participation | - Attendance at online support group sessions as scheduled  - Engagement with educational booklet and supplementary website |
| **4. Reflexive Monitoring**  (How do people appraise intervention?) | - Appraised during meetings  - Fidelity checklist | - Appraised during meetings | - Appraised during meetings | - Opportunity for input / feedback across the online support group sessions |

Legend: NPT = Normalisation Process Theory; HELP = HEart faiLure carer support Programme; HF = Heart Failure; and PI = Principal Investigator.

**INPUTS**

- HELP is an evidence-based, psychoeducational approach to supporting carers of patients with HF. This intervention was co-designed with carers and healthcare professionals in the UK and consists of two components *(Intention, Environmental Context, and Resources)*:

**Programme Duration: 6 weeks**

- Deliverable by specialist HF nurses within HSC / NHS services *(Environmental Context and Resources; Social/Professional Role and Identity).*
- Training and supervision for HELP delivery staff to ensure intervention fidelity *(Knowledge and Skills; Optimism*; *Social/Professional Role and Identity; and Beliefs about Capabilities).*
- Willingness of organisation (HSC / NHS HF teams) to identify staff and deliver the intervention *(Goals; Intentions; Environmental Context and Resources).*

**CONTEXT**

Many patients with HF rely on support from family carers who are ill-prepared and supported for this role. As a result, these carers often experience poor mental and physical health, which has been linked to their caring role and the health status of the patient they care for.

**CLINICAL IMPLICATIONS**

Carers of patients with HF require psychoeducational support to improve their QoL and better prepare them for their caring role. However, there is a paucity of interventions co-designed with carers in the UK to exclusively target these requirements.

**ACTIVITIES**

- Psychoeducational support for carers on understanding HF and strategies for improving physical and emotional wellbeing for themselves and their loved ones *(Knowledge, Skills, Behavioural Regulation, Beliefs about Capabilities, Optimism, and Emotion).*
- Tailored advice, support, and signposting for overcoming problems related to caring for a person with HF *(Knowledge, Skills, and Goals).*
- Establishment of a peer-support network that facilitates collaboration and communication between carers *(Social Influences).*
- Practical support and troubleshooting with accessing and operating the online support group sessions and supplementary website *(Environmental Context and Resources).*
- Delivery of training to HELP facilitators and provision of standardised materials to guide the intervention and embed the skills in practice *(Skills; Social/Professional Role and Identity; Environmental Context and Resources).*

**MECHANISMS OF IMPACT**

- Improved knowledge and skills across key areas of caring for a person with HF (*i.e.,* symptom management, palliative care, and communication) *(Knowledge; Skills; Beliefs about Capabilities; Optimism).*
- Equipping carers with strategies to improve the physical and emotional wellbeing of their loved ones with HF and themselves *(Knowledge, Skills, Behavioural Regulation, Beliefs about Capabilities, Optimism, and Emotion).*
- Promoting collaboration and improved communication between carers, patients, and healthcare professionals *(Social Influences).*
- Equipping carers with tailored advice and support for overcoming problems related to caring for a person with HF *(Knowledge, Skills, and Goals).*
- Participation in a peer-support network that facilitates collaboration and communication between carers *(Social Influences).*
- Verbal education supplemented with digital and written resources to support and facilitate learning *(Knowledge, Skills, Goals).*
- Organisations provided with the training, skills, and standardised materials required for intervention delivery *(Skills; Social/Professional Role and Identity; Environmental Context and Resources).*

**1. Online Support Group Sessions**

- Delivered on ZOOM by specialist HF nurse

- 1 weekly session (60 mins each) with 12 carers

- Standardised presentations on different topics (approx. 20 mins)

- Group discussion and peer support (approx. 40 mins)

**2. Educational Booklet and Supplementary Website**

- Educational supplement for the support group sessions

- Self-directed learning between support group sessions

- Question submission (website)

- Previews of support group presentations (website)

**OUTCOMES**

**SHORT-TERM**

**Carers of patients with HF**

- Greater understanding of HF and the role of caring for someone with this condition (improved carer preparedness)
- Better equipped for caring role and supporting the patient with self-management (decreased carer burden)
- Improved communication with patients and healthcare professionals (increased social support)
- New skills to better manage physical and emotional wellbeing (decreased anxiety, depression, and stress; improved QoL, overall)

**Patients with HF**

- Improved support with self-management from carer (increased QoL)

**System**

- Staff trained to deliver a novel, evidence-based intervention
- Improved service delivery and support for carers of patients with HF

**LONG-TERM**

**Carers of patients with HF**

- Greater knowledge and skills with caring role and supporting the patient with self-management (improved carer preparedness and decreased carer burden)
- Equipped with strategies to manage physical and emotional wellbeing (decreased anxiety, depression, and stress; improved QoL, overall)
- Improved relationship with patient and healthcare professionals (increased social support)

**Patients with HF**

- Sustained support with self-management from carer (increased QoL)
- Fewer hospital admissions and GP visits

**System**

- Efficient use of resources: meeting needs of carers
- Organisational change in supporting carers
- Reduction in complications for patients (*i.e.,* hospital admissions / GP visits)

**Figure 2. HELP Logic Model.**

Legend: HF = Heart Failure; QoL = Quality of Life; UK = United Kingdom; HELP = HEart faiLure carer support Programme; HSC = Health and Social Care; NHS = National Health Service; and in parentheses () and italics = mapping to Theoretical Domains Framework.

#### **Intervention Delivery**

The online support group sessions will be delivered via ZOOM by a local, specialist HF nurse at each collaborating centre (3 in total; 1 nurse per Northern Ireland (based at Royal Victoria Hospital), England (based at King’s College Hospital), and Scotland (based at Glasgow Royal Infirmary)). These local, specialist HF nurses will be confirmed when the project start date is ascertained following acquisition of ethical and governance approvals. Clinical contacts have identified suitably qualified specialist HF nurses who will be offered this opportunity. Funding will be provided to the Trusts / NHS sites to cover time commitments (max. 0.5 WTE) of the nurses. Carers who provided consent will be contacted by the project manager (GT) to receive a start date for HELP commencement. One week before the first online support group session, participants will receive the educational booklet (see Appendix 7) via post and programme materials via email, which include: programme schedule (see Appendix 8), instructions on how to join the ZOOM sessions (see Appendix 9), and a link to the supplementary website for the option of accessing the information from the educational booklet in a digitalised format. Carers will be advised to read each chapter of the educational booklet, and if interested, the supplementary website before the corresponding support group session for familiarisation with content. A link and password to each ZOOM session will be issued via email one week before commencement, followed by a reminder email two days before the planned session. The project manager (GT) will remain in contact with all participants prior to the commencement of the support group sessions through email and telephone to provide ongoing support with accessing the website and ZOOM sessions.

The online sessions will be delivered by the specialist HF nurses using an informal approach to facilitate a comfortable environment for carers. Each session will consist of 12 carers and last approximately 60 minutes. The sessions will initiate with a presentation (approximately 20 minutes in duration) on the allocated topic, including questions and group discussions throughout. The support group sessions will provide an opportunity for tailored support, with questions submitted via the website answered and signposting to relevant information (*i.e.,* from charities such as HF Warriors Northern Ireland, Pumping Marvellous, and BHF). Participants will receive the option of having their camera switched on or off during the sessions and will be encouraged to use the chat function on ZOOM to ask questions if they did not feel comfortable speaking out in front of the group. The facilitator will monitor the ZOOM chat function throughout each session. The specialist HF nurses responsible for HELP delivery across the sites will use standardised material and receive training (discussed above) from the project team on consistent delivery and group engagement. Attendance at each support group session will be recorded (see Appendix 10).

#### **Data Collection**

Quantitative and qualitative data will be collected across four time points: 1) Baseline, 2) Intra-intervention, 3) Post-intervention (week 6), and 4) 6 months post-intervention.

##### **Demographic Characteristics**

To contextualise the results, carers will be asked to complete a demographic questionnaire (see Appendix 11) at baseline to provide information on age, gender, ethnicity, employment status, relationship status, disabilities, education level, relationship with patient, duration of care provision to patient, and post code (measure of socioeconomic status). Patients will also be requested to complete a demographic questionnaire (see Appendix 12) at baseline detailing age, date of birth, gender, ethnicity, employment status, relationship status, disabilities, education level, and post code (measure of socioeconomic status). Date of birth is collected for patients to allow correct identification when UKCRN nurses complete data extraction from medical records (*i.e.,* differentiation between patients with same name). Both carers and patients will receive the option of completion via a telephone call or Microsoft Forms under the guidance of the project manager (GT).

##### **Fidelity (Objective 1)**

Fidelity of HELP implementation will be assessed by evaluating what was implemented and how closely this reflected the ‘intervention-as-designed’ (what was intended). This assessment will be guided by the fidelity framework published by the National Institutes of Health Behaviour Change Consortium (21), which includes the following:

1) Study Design: deviation from the study protocol may negatively influence HELP implementation (21). Therefore, protocol / intervention operationalisation will be monitored by the Intervention Oversight Group (described above). This group will receive updates from the Project Manager (GT) on protocol / intervention implementation across all sites. Any protocol deviations will be discussed and a plan for future prevention established. This information will be documented in an implementation log (see Appendix 13).

2) Provider Training: competent intervention delivery staff increase the likelihood of protocol compliance (21). Following training, the competency of HELP delivery staff will be assessed during a ‘role play’ session, which will involve an intervention fidelity checklist that ascertains the presence or absence of each intervention component (see Appendix 14).

3) Intervention Delivery: the HELP sessions will be audio-recorded. Recordings will be assessed using the intervention fidelity checklist (see Appendix 15), which focusses on 1) Interactional style (*i.e.,* use of a carer-centred approach); 2) Intervention components covered (*i.e.,* required educational topics for each session) and those components omitted; 3) Components added that were not specified by the protocol (*i.e.,* an educational topic not pre-specified); 4) Dosage (session duration); and 5) Competency (maintenance of skill set learned in training [i.e., specialist psychoeducational support and facilitating group discussion]). HELP delivery staff will complete a facilitator log (see Appendix 16) following each session to document any complications or required amendments to intervention implementation. The feasibility of HELP operationalisation by delivery staff will also be qualitatively explored via interviews (discussed below).

4) Treatment Receipt & 5) Enactment: carers’ understanding of the information provided and ability to implement the skills and recommendations delivered by HELP in the real world will be investigated in interviews (discussed below).

**Contextual Factors (Objective 1)**

Contextual factors (*i.e.,* barriers and facilitators) influencing HELP implementation will be investigated via:

1) Individual interviews with carers, patients, healthcare professionals, and HELP delivery staff (discussed below).

2) Contextual data (*i.e.,* ‘background noise’ and protocol deviations) collated centrally in the implementation log (see Appendix 13).

3) Intervention acceptability questionnaires completed by carers (*i.e.,* suitability of intervention components, digital literacy, and opinions of supplementary website) post-intervention (see Appendix 17) and HELP facilitators (*i.e.,* acceptability of delivery) upon project completion (see Appendix 18). These questionnaires will be completed via a telephone call or Microsoft Forms, whichever preferable for the participant, under the guidance of the project manager (GT).

###### **Interviews Exploring Fidelity and Contextual Factors (Objective 1)**

**Design**

Individual, semi-structured interviews (approximately 60 minutes in duration) will be conducted with carers who received the intervention, patients, healthcare professionals, and HELP delivery staff. Carers and patients will be invited to participate 6 months post-HELP completion, with healthcare professionals and HELP delivery staff invited at the end of the study. A core set of questions in a “laddered style approach” will be used, which will ensure emerging ideas are explored (22). Interview questions will be based on the domains of CFIR (1. Intervention characteristics, 2. Outer setting, 3. Inner setting, 4. Characteristics of individuals, and 5. Implementation process) and NPT (1. Coherence, 2. Cognitive Participation, 3. Collective Action, and 4. Reflexive Monitoring) (13). In summary, interviews with carers (see Appendix 19) will focus on their experience with HELP, required improvements, barriers and facilitators to delivery, impact of HELP, and long-term adherence to support provided. Interviews with patients (see Appendix 20) will investigate their opinions of HELP, required improvements, barriers and facilitators to delivery, how HELP impacted the support from their carer, and long-term adherence to support provided. Finally, interviews with healthcare professionals (see Appendix 21) and HELP delivery staff (see Appendix 22) will explore their opinions of HELP, required improvements, and barriers and facilitators to delivery.

**Participants**

As part of the original consent process (see Appendices 4 & 6), carers and patients will either agree or refuse to be contacted about a possible interview 6 months post-HELP completion. HELP delivery staff and healthcare professionals (HF consultants and HF nurses) across the collaborating sites will be invited to participate and the end of the study and will be eligible if they are 1) Aged 18 and over and 2) Willing to provide informed consent.

**Sample Size**

Approximately 33 interviews will be conducted with carers (n = 10), patients (n = 10), HELP delivery staff (n = 3), and healthcare professionals (n = 10 in total; n = 5 HF nurses and n = 5 HF consultants). This sample size is based on recommendations in the literature for a study of this scope (23). A purposive sampling strategy (24) will be used to maximise representation and diversity across the following variables: age, gender, ethnicity, education, socio-economic status, relation to patient (carers only), left ventricular ejection fraction (LVEF; patients only), and programme completion rates (1. < 50%, 2. 50% – 70%, and 3. > 70% [carers only]). An equal number of cases will be recruited across the collaborating centres.

**Procedure**

6 months post-HELP, the project manager (GT) will contact selected cases (carers and patients) who agreed to be contacted about a possible interview. If they are still interested, the project manager will send them information sheets (see Appendices 23 & 24) via email. Following a 1-week, ‘cooling-off’ period, the project manager will contact patients and carers to answer any questions and receive verbal consent (see Appendices 25 & 26) before arranging a suitable time for a telephone interview. At the end of the study, the project manager (GT) will request for clinical staff from the project team to supply interested colleagues (healthcare professionals; HF consultants and HF nurses) with information sheets (see Appendix 27) for the interviews. The information sheets will advise potential participants to consider their willingness to participate for a 1-week, ‘cooling-off’ period before contacting the project manager (GT) who will answer any questions, receive verbal consent (see Appendix 28), and arrange a suitable time for a telephone interview. As HELP delivery staff are members of the project team, they do not require information sheets. The project manager will communicate directly with them to receive verbal consent (see Appendix 29) and arrange a suitable time for the interview. Prior to interviews, healthcare professionals and HELP delivery staff will be asked to provide demographic details (age, gender, ethnicity, education level, relationship status, occupation, and postcode [measure of socioeconomic status]) via Microsoft Forms (see Appendix 30), which will set the findings in context. All interviews will be audio-recorded before being transcribed verbatim by an external service who has completed a data sharing agreement with QUB.

##### **Patient and Carer-Related Outcomes (Objective 2)**

The effect of HELP on relevant patient and carer-related outcomes will be assessed using questionnaires and data from medical records. Data will be collected across four time points: 1) Baseline, 2) Intra-intervention (weeks 1 – 6), 3) Post-intervention (week 6), and 4) 6 months post-intervention. Both carers and patients will receive the option of questionnaire completion via a telephone call or Microsoft Forms under the guidance of the project manager (GT). Patient diaries will be emailed to these participants for self-completion over the study period (baseline to 6 months), with pseudonymised, completed diaries (electronic or scanned) emailed to the project manager at the final time point (6 months). With patient consent, data from medical records (LVEF and number of hospital admissions with reasons) will be extracted by UKCRN staff using a data extraction form (see Appendix 31).

**Baseline**

**Carers**

1) Questionnaire booklet (see Appendix 32) containing 6 short, validated questionnaires:

1. Zarit Burden Interview, Short Form (25): carer burden
2. Preparedness for Caregiving Scale (26): carer preparedness
3. 12-Item Short Form Survey (27): quality of life
4. Hospital Anxiety and Depression Scale (28): anxiety and depression
5. Perceived Stress Scale (29): stress
6. Multidimensional Scale of Perceived Social Support (30): social support

**Patients**

1) Questionnaire booklet (see Appendix 33) containing 2 validated questionnaires:

1. Minnesota Living with Heart Failure Questionnaire (31): disease-specific, health-related quality of life
2. EuroQol Five Dimension Questionnaire (32): generic, health-related quality of life

2) Brief questionnaire (see Appendix 44) on number of GP visits with reasons over previous 6 months.

3) Most recent LVEF measurement and number of hospital admissions with reasons over the previous 6 months: data extracted from medical records (see Appendix 31).

4) Patient diary documenting GP visits with reasons over study period (self-completed from baseline to 6 months [see Appendix 45]).

###### **Intra-Intervention (Weeks 1-6)**

**Carers**

1) The impact of HELP on carers’ behaviour each week over the 6-week programme will be assessed using a brief, bespoke questionnaire (Weekly HELP impact questionnaire: Carers; see Appendix 34). This data will be collected after each support group session.

**Patients**

1) The patients’ perspectives on how HELP has impacted the carers’ behaviour each week over the 6-week programme will be assessed using a brief, bespoke questionnaire (Weekly HELP impact questionnaire: Patients; see Appendix 35). This data will be collected after each support group session.

###### **Post-Intervention (Week 6)**

1) Carers and patients will complete the same questionnaire booklets as baseline (see Appendices 32 & 33), with carers also completing the intervention acceptability questionnaire (see Appendix 17).

###### **6 Months Post-Intervention**

**Carers**

1) Same questionnaire booklet as baseline and post-intervention (see Appendix 32).

2) Bespoke questionnaire examining long-term adherence to the support and advice provided by HELP (see Appendix 36).

**Patients**

1) Same questionnaire booklet as baseline and post-intervention (see Appendix 33).

2) Bespoke questionnaire examining long-term adherence to the support and advice provided by HELP (see Appendix 37).

3) Email submission of patient diary documenting GP visits with reasons over study period (see Appendix 45).

4) Most recent LVEF measurement and number of hospital admissions with reasons over the previous 6 months: data extracted from medical records (see Appendix 31).

##### **Economic cost of HELP implementation in the UK (Objective 3)**

The economic cost of HELP implementation in the UK will be estimated via the collection of the following data:

1) Implementation log (see Appendix 13) detailing training costs and time input for supervision of HELP facilitators.

2) Facilitator log (see Appendix 16) for each session documenting time, expenditure, resources required, and adaptations made for individual carers.

#### **Data Collection Summary**

Figure 3 displays a summary of data collection across the objectives of the project.


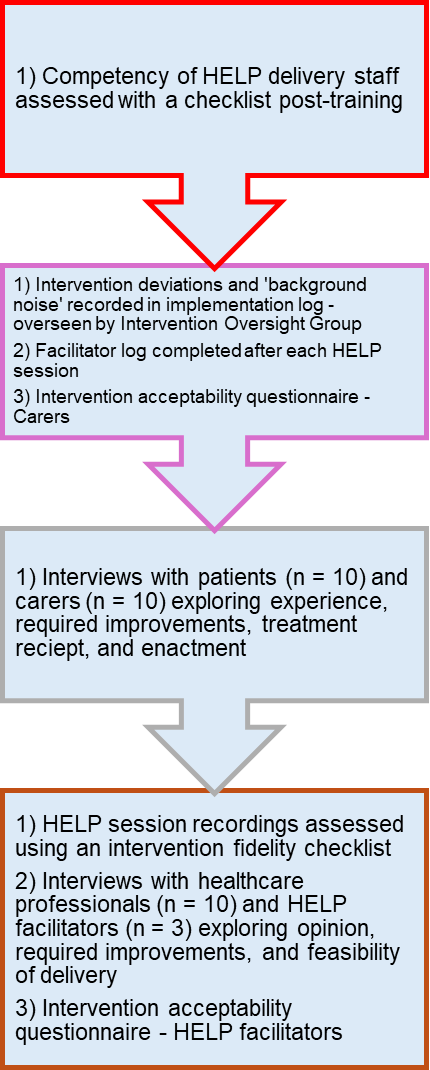


**Objective 1: Fidelity & Contextual Factors**


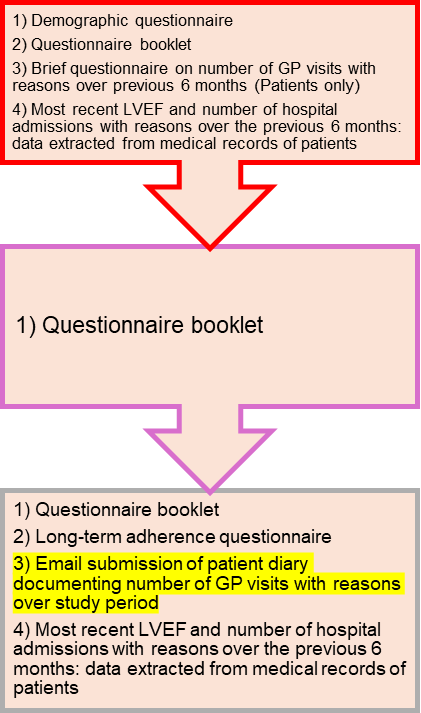

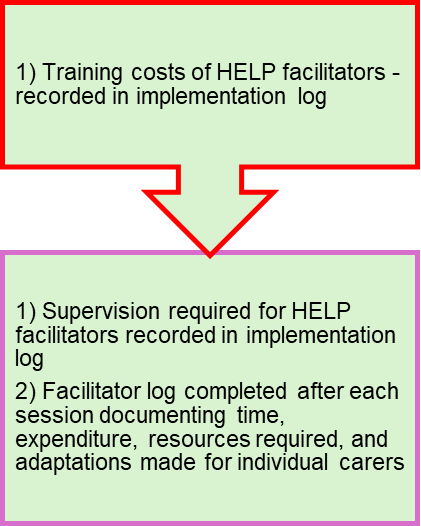


**Objective 2: Patient and Carer-Related Outcomes**

**Objective 3: Estimated Economic Cost of HELP Delivery**

**Baseline**

**Post-Intervention (6 weeks)**

**6 Months Post-Intervention**

**Study End**

**Figure 3. Data Collection Summary.**

**Intra-Intervention** **(Weeks 1-6)**

1) Weekly HELP impact questionnaire

##### **Case Report Form**

The project manager (GT) will record data collected from each carer and patient on a pseudonymised Case Report Form (see Appendices 38 & 39), which will serve as a consolidated resource that outlines the data collected for each participant.

### **Data Analysis**

#### **Quantitative Data Analysis**

Quantitative data will be managed on Statistical Package for the Social Sciences (IBM SPSS Statistics, Version 29). Descriptive statistics will be used to profile the sample. Continuous data will be presented as mean ± standard deviation, with discrete data reported as absolute numbers and percentages. Categorical data will be displayed as frequency / percentages. A within-group comparison from baseline to follow-up will be performed and presented as mean difference with 95% confidence interval, with results interpreted according to minimum clinically important differences, if available.

#### **Qualitative Data Analysis**

The audio-recordings of semi-structured interviews will be transcribed verbatim by an external service who has completed a data processing agreement, with transcripts pseudonymised. Qualitative data analysis will be managed on NVivo (QSR International Pty Ltd. Release 1). Interview transcripts will be analysed using adaptive framework analysis (33) based on the CFIR and NPT domains (13), whilst allowing the integration of data that could not be placed in pre-specified categories. An a priori codebook will be established based on the CFIR and NPT domains, which will guide the analysis. Definitions of CFIR and NPT domains will be tailored to the project, which will improve coder consistency. The project manager (GT) will perform the coding process by mapping major themes to the CFIR and NPT domains. Quotes from participants will be used to support the generation of themes. A second member of the project team (DF) will independently analyse and verify a random selection of transcripts. Subsequently, the results will be reviewed by the wider project team, with discrepancies discussed until consensus agreement is reached.

##### **Integration of Quantitative and Qualitative Data**

An integrative analysis will be conducted to bring together each analytic component (fidelity, context, and patient and carer-related outcomes). A triangulation protocol will be implemented according to the recommendations of Farmer et al. (34). The quantitative and qualitative data will be compared to ascertain if there is agreement, partial agreement, discrepancy, or silence between them. This assessment will be displayed in a convergence coding matrix. Key considerations are the identification of synergies across barriers and facilitators to HELP implementation (fidelity and context), along with patient and carer-related outcomes (*i.e.,* qualitative evidence of behaviour change supported by quantitative outcomes). Two members of the team (GT and DF) will integrate quantitative and qualitative data separately. Inter-rater reliability will be calculated (*i.e.,* percent agreement), with discrepancies discussed with the wider project team until consensus agreement.

#### **Economic Analysis**

The cost of HELP delivery across the collaborating centres will be estimated using resource utilisation data collected during the project and unit costs from relevant UK reference costs (35). Resource use will consist of time input from HELP facilitators, supervision for facilitators, training costs for facilitators, and consumables / adaptations required for HELP delivery. Delivery costs will be estimated at session and programme-level and reported in pounds sterling (£).

## **Discussion**

To the best of our knowledge, HELP represents the first co-designed service for carers of patients with HF across the UK, which addresses a critical area of unmet emotional and educational need (3,4,36). Whilst pilot work demonstrated feasibility and a positive impact on carer-related outcomes (12), the results do not inform policy-makers or service commissioners whether HELP would generate the same outcomes in a real-world, clinical setting (37). Complex interventions often require adaptation when they are transferred to the real-world setting to overcome contextual barriers, with a degree of intervention flexibility improving pragmatic effectiveness (38). Therefore, prior to large-scale, routine implementation, it is vital to have a clear understanding of the active components of HELP, and a knowledge of how closely intervention delivery in a real-world, clinical setting follows what is intended (13). Moreover, it is important to have a clear picture of the impact of any required contextual adaptations on intervention integrity and outcomes for participants (11).

The challenges related to real-world intervention implementation will be ameliorated by this project. In line with MRC guidance (10), a comprehensive, mixed-method process evaluation of HELP delivery by local healthcare professionals across the UK will be conducted. This will identify the factors underpinning successful HELP translation to clinical settings, with a focus on fidelity to the intended programme and how contextual factors / adaptations influence delivery and participant outcomes. The economic cost of HELP delivery will also be estimated. These findings will inform large scale, embedding of HELP in routine clinical practice, offer guidance to policy-makers and commissioners, and identify required contextual adaptations. Moreover, this project will establish Beacon Sites across three regions of the UK (Northern Ireland, England, and Scotland), which will support future HELP roll-out by modelling intervention implementation (16).

### **Potential Impact**

The findings may lead to large-scale, national implementation of a novel intervention that provides essential psychoeducational support to carers of patients with HF across the UK. If successful, the HELP concept may be expanded beyond the UK and generate parallel products / services that are applicable to patients with HF and other disease and carer populations.

## **Ethical Considerations**

This study will be conducted in accordance with Good Clinical Practice guidelines and adhere to the Declaration of Helsinki statement (1964). Ethical and governance approvals will be sought from QUB, NHS / HSC Research Ethics Committee, and local NHS / HSC R&D offices using IRAS. The project team have prior experience with successful ethical applications and understand the regulations and timescales involved. This is a low-risk intervention delivered by experienced clinical staff. We have consulted with Professor Mike Clarke (methodological expert), along with patients, carers, and healthcare professionals in the development of this protocol and the intervention to mitigate any areas of ethical concern. We will liaise with BHF (Funder) if any significant changes to the protocol are required.

### **Participant Identification and Recruitment**

This study has broad eligibility criteria and a simple recruitment strategy to allow ease of access for participants. A log will be used by the project manager (GT) to identify any complications with potential participant identification and recruitment (see Appendix 13). Training will be delivered by the project manager to local cardiology teams (*i.e.,* HF nurse or research nurse) with patient screening and approaching potential participants. These staff will receive prompt updates on any changes to the study protocol and eligibility criteria. They will also receive a flow diagram (see Appendix 40) and factsheet to answer commonly asked questions (see Appendix 41) for guidance.

The Steering Group (including PPI members) have advised on the development of all study documentation and participant-facing information to ensure accessibility and adherence to plain English guidelines. Whilst this project is limited to English-speaking carers and patients, our Steering Group will ensure the intervention is optimised for future language translation and access across socioeconomic classes (*i.e.,* access possible on a mobile phone without having a laptop / desktop). PPI input highlighted the requirement to clearly discuss time commitments and the use of questionnaires to obtain measurements with potential participants. Therefore, the project manager will be explicit during the consent process to ensure the potential participant has the required knowledge to provide informed consent. Verbal consent was chosen to maximise convenience for participants, with this decision based on PPI input and verbal consent being the preferred method for participants in the pilot study (12).

### **HELP Delivery / Digital Access**

HELP design and delivery are based on significant input from carers and professionals, which maximises suitability for recipients. The pilot results provide evidence of feasibility and acceptability for carers (12). HELP delivery staff will receive comprehensive training from the project team and standardised materials to ensure consistent, high-quality intervention delivery. The project team and Steering Group have considered digital poverty throughout the design of the study protocol. IT access / connectivity was not a barrier to participation in the pilot study (12), with the results demonstrating that carers viewed online sessions as accessible, acceptable, and beneficial. This supports the literature documenting the time and cost benefits of online sessions by avoiding travel to a venue in-person (39). In addition, it facilitates wider geographical spread and enables people in rural areas, those with physical disabilities or transport challenges to avail of the service. Moreover, online delivery carries environmental benefits by circumventing the carbon emissions of travel, which supports the ‘Greener NHS’ programme (40) and the UK Government’s ‘25 Year Environment Plan’ (41). Remote delivery also represents a more financially feasible method for future large-scale, routine implementation, and is consistent with the shift towards online delivery of healthcare services following COVID-19. The project team have significant experience with successfully conducting other large, multi-centre remote technology programmes (42). We will ensure participants are aware of IT requirements (*i.e.,* access to a smart phone / device or computer / laptop with a strong network signal) and will provide ongoing support (*i.e.,* walking carers through the process of accessing the supplementary website and joining online sessions before formal delivery). Should owning a digital device be a barrier to participation in the project, we have a stock of digital devices to provide to those without access. For these individuals, a digital device will be securely posted by the Project Manager to a participant’s home address, before being returned via post after intervention completion (postage cost covered by QUB). The devices supplied will be in good working condition, with memory / data erased (*i.e.,* factory default settings).

### **Anonymity**

The identification of eligible patients will be completed by local cardiology teams (direct healthcare team). These individuals will make primary contact with eligible patients, share information sheets, and receive permission from interested patients for them to be contacted by the project manager (GT). Interested patients will also pass information sheets to their carers for consideration. A one-week ‘cooling-off’ period will be implemented for potential participants to consider participation before being contacted by the project manager (GT). The project team will not access any records for potential participant identification. Medical records will only be accessed by UKCRN staff for the extraction of outcome data for consenting participants. Each participant will be assigned a Personal Identification Number (PIN), which will replace identifying information within datasets, such as name and hospital Numbers. Accessing the supplementary website will only require email address and project PIN. The email address will only be used for password reset purposes and will not be shared. No other personal data will be collected by the website. In the information sheets and during the consent process, participants will be informed that the supplementary website will track their activity (*i.e.,* time spent, and modules accessed) to contextualise engagement and potential changes in outcomes. This activity data will be linked with PIN. The website is managed by Focus Games Ltd who are a GDPR registered and compliant organisation.

### **Burden**

Project procedures and materials were informed by the pilot study and Steering Group to minimise participant burden and maximise suitability. All participant-facing materials are user-friendly in design and approved by the Steering Group. The number of questionnaires in the pilot study demonstrated no data completeness issues. This project will include the same questionnaires plus two additional, brief bespoke questionnaires, with all data collection items taking approximately 20 mins to complete in total at each time point. The Steering Group have deemed the proposed questionnaires acceptable. Data collection will be conducted remotely via telephone or Microsoft Forms, depending on participant preference, which offers the convenience of avoiding participant travel. Participants will be clearly informed during the consent procedure and by the information sheets that the website is an optional supplement to the educational booklet, which gives carers an opportunity, if interested, to receive the same educational information in a digitalised format. Participants who complete the project will receive electronic vouchers as compensation for their time and efforts.

### **Participant Safety**

The project team possess expertise with the ethical issues surrounding research with patients who have HF and their carers (43). We have identified that it is important to be sensitive to their needs and have established strategies to ensure participant safety and minimise any distress. The project team will remain flexible and mindful of the physical and emotional health of carers and patients. As a patient’s condition can vary on a daily basis, it may be difficult for carers and patients to attend and complete scheduled intervention or data collection sessions. Therefore, breaks will be offered during intervention and data collection sessions, and we will be prepared to reschedule data collection sessions to better suit participants, if necessary. This flexibility will enhance participation and retention, whilst supporting the participants.

Sensitive topics (*i.e.,* palliative / end-of-life care) discussed during the support group sessions may cause distress for the carers. HELP delivery staff will assess the preferences of carers when discussing such issues and employ terminology that carers are comfortable with. In the event of participant distress during HELP sessions or data collection, a protocol (see Appendix 42) will be implemented to ensure effective management. This will involve HELP delivery staff or the Project manager offering a break or changing the topic of conversation. If distress is moderate or severe, the session will be stopped, and the participant will be asked if there is a relative or friend he / she would like to contact. If necessary and with the participant’s permission, the Project Manager will verbally inform the participant’s direct healthcare team (*i.e.,* Cardiologist) for further support. The Project Manager will carry out a follow-up telephone call 24 hours later to check-in on the participant, and if he / she is still experiencing distress, encouragement to contact his / her GP / Cardiologist will be given. Participants will receive the contact details of the Project Manager and Chief Investigator in case help or support is needed.

Due to the unpredictable disease trajectory of HF, there is a possibility of patient death during the project. If such an event happens, the project team will handle this sensitively and give carers space before following up with them about willingness to continue with programme participation. Carers will also be signposted to local bereavement support, where applicable.

Given the brief nature of the questionnaires and scales, the Project Manager will calculate the corresponding scores on the same day as data collection. Participants who demonstrate low overall scores will be immediately highlighted by the Project Manager to the direct healthcare team (*i.e.,* Cardiologist). This will allow rapid identification of those patients or carers in need of additional support. It will then be at the discretion of the direct healthcare team as to what extra support is provided to these individuals. Participants will be clearly informed by the information sheets and during the consent process that the question submission function of the website is unsuitable for urgent medical queries. This disclaimer will be presented on the website, with advice on contacting emergency services for such issues.

### **Study Withdrawal**

Participants will be informed of the voluntary nature of their inclusion in this project and their ability to withdraw at any point, without compromising their current clinical care or that of their loved one. As stated in the information sheets, if a participant withdraws 2 weeks after the time of initial data collection, his / her data may be used for analysis (with the participant’s consent). The reason for withdrawal will be noted for review. As HELP targets carers, they can continue participating, if interested, in the event of patient withdrawal. However, as the project is focused on carers, carer withdrawal will also result in patient withdrawal. The information sheets will clearly specify these requirements.

### **Data Protection and Management**

The requirements of the General Data Protection Regulation and Data Protection Act (2018) [<https://www.hra.nhs.uk/planning-and-improving-research/policies-standards-legislation/data-protection-and-information-governance/gdpr-guidance/>] will be adhered to, as per the QUB Research Data Management Policy [<https://www.qub.ac.uk/directorates/InformationServices/TheLibrary/CustomerService/PoliciesandRegulations/ResearchDataManagementPolicy/>]. All information will be stored in a secure manner in compliance with local NHS / Trust governance requirements. We will only acquire information that is required to undertake the project. All paper data will be stored in a Master File and Site Files in locked filing cabinets, inside locked offices within the School of Nursing and Midwifery, QUB and the collaborating centres, respectively. All electronic data will be stored on an encrypted and password protected computer, inside a locked office within the School of Nursing and Midwifery, QUB. Individual data files will also be password protected. All audio-recordings of interviews and support group sessions will be destroyed after the transcripts have been checked for accuracy. Audio-recordings of verbal consent will be stored on an encrypted and password protected computer, inside a locked office within the School of Nursing and Midwifery, QUB.

Personal data will be pseudonymised. A PIN system will be used, whereby each participant is allocated a unique number. This number will replace identifying information within datasets, such as: name and hospital number. This will help preserve anonymity when the data is reviewed by the project team. A master list that links PINs to personal information (*i.e.,* name and contact details) will be stored securely and separately from other data in a locked office within the School of Nursing and Midwifery, QUB. Transcriptions of interview and support group session audio-recordings will be redacted and transferred through secure processes (*i.e.,* linked with PIN). We will only use trusted staff who have completed a data sharing agreement with QUB for transcribing audio-recordings. All data presented in publications will be anonymised to protect participant confidentiality.

Measures will be taken to protect data transfer between QUB and the collaborating sites. When discussing the project with eligible, interested patients, local cardiology teams (*i.e.,* HF nurse or research nurse) will document patient permission to be contacted by the Project Manager and record their contact details on a form (see Appendix 43), which is stored in the Site File. These staff will only communicate the names and contact details of willing patients to the Project Manager by encrypted email. If applicable, the email addresses used by local cardiology teams to send information packs to potential participants will not be shared with the project team. For the extraction of data from medical records, the Project Manager will send an encrypted email to UKCRN staff to provide the names, date of birth (for differentiation between patients with the same name), and PINs of consenting patients for whom data is required. When completed, UKCRN staff will store original versions of the data extraction form (see Appendix 31) in the Site File and send scanned, anonymised and password-protected versions to the Project Manager via encrypted email. UKCRN staff will telephone the Project Manager to inform him of the password for accessing these documents. Upon confirmation of receipt by the Project Manager, UKCRN staff will delete any saved, scanned versions on their emails and devices (*i.e.,* desktop computer or laptop).

Consent will be obtained from participants for data preservation and sharing in an anonymised format. As per the QUB Research Data Management Policy [<https://www.qub.ac.uk/directorates/InformationServices/TheLibrary/CustomerService/PoliciesandRegulations/ResearchDataManagementPolicy/>], data will be retained in an anonymised format for a minimum of 5 years following publication if they are of continuing value to the researchers and the wider research community. Upon project completion, data sets will be allocated a DOI and transferred to QUB’s research information management system (Pure) for long-term preservation, which will contain links to the project and relevant publications. All data that may identify participants will be destroyed following QUB procedures. Data will be exclusive to the team until project completion. After which, data may be shared in an anonymised format with participant consent. The Chief Investigator (DF) will make the decision about whether to supply data to a potential new user. Before data is shared, a data-sharing agreement will be issued and signed by appropriate authorities, which will prohibit any attempt by external users to (a) identify participants form the released data or otherwise breach confidentiality and (b) make unapproved contact with any participants. The MRC Policy and Guidance on Sharing of Research Data from Population and Patient Studies [<https://www.ukri.org/publications/mrc-guidance-on-sharing-research-data-from-population-and-patient-studies/>] will be adhered to. The Chief Investigator (DF) will be the first point of contact for all queries in relation to the data and will also have overall responsibility for the production and maintenance of metadata. The project manager (GT) will have responsibility for project-wide data management, metadata creation, data security, and quality assurance of data. Preparation and upload of the data will be carried out by the project team with the support of QUB Information Services staff.

## **Dissemination**

The protocol and results of this study will be disseminated via publications in peer-reviewed journals and scientific conferences (national and international). We will link with BHF to publicise the project and findings. We will utilise opportunities within QUB to disseminate project information and results, for instance, post-doc events and the School of Nursing and Midwifery Annual Conference. Members of the Steering Group (*i.e.,* patients and carers) will be involved with dissemination of results and co-authorship of papers/abstracts where possible. We will arrange a dissemination event for key stakeholders, which will ensure attention is drawn to the capacity and requirements for scaling-up and regional implementation of HELP. To achieve this, we will harness our strong relationships with healthcare professionals; professional organisations and medical charities (*i.e.,* European Society of Cardiology, BHF, Northern Ireland Chest Heart and Stroke, and Association of Cardiovascular Nursing & Allied Professions), innovation networks (*i.e.,* Northern Ireland Clinical Research Network); and policy-makers and commissioners across the UK.

## **End of Study**

This study will end following the completion of data collection and analysis, which will occur approximately 18 months after ethics and governance approvals.

## **Project Milestones**

**Table 4.** Project Milestones

| **Date** | **Milestone** | **Project Month** | **Person Responsible** |
| --- | --- | --- | --- |
| February 2025 - July 2025 | Ethics and Governance Approvals | 1 - 6 | Project Manager |
|  | Publicising Study Initiation |  | Entire Team |
|  | Employment and Training of HELP Delivery Staff (n = 3 Specialist HF Nurses; n = 1 at each Centre) |  | Project Manager and CI |
|  | Securing UKCRN Adoption |  |  |
|  | Training UKCRN Staff and Clinical Collaborators |  |  |
|  | Writing and Publishing Protocol Paper |  | Entire Team |
| August 2025 – July 2026 | Recruitment and HELP Delivery | 7 - 18 | Project Manager and HELP Delivery Staff |
| August 2025 – January 2027 | Data Collection from Patients, Carers, and HELP Delivery Staff (1. Baseline, 2. Post-Intervention, and 3. 6-Month Follow-Up) | 7 - 24 | Project Manager |
| October 2026 - January 2027 | Qualitative Data Collection from Staff | 20 - 24 | Project Manager |
| August 2025 – January 2027 | Data Analysis | 7 - 24 | Entire Team |
| Post-Project | Dissemination of Findings | Post-Project | Entire Team |

HELP = HEart faiLure carer support Programme, HF = Heart Failure, UKCRN = United Kingdom Clinical Research Network, and CI = Chief Investigator.

## **Project Deliverables**

**Table 5.** Project Deliverables.

| **Deliverable** | **Context** | **Month of Delivery** | **Person Responsible** |
| --- | --- | --- | --- |
| Protocol Paper | 1. Publication in peer-reviewed scientific journal | 6 | Entire Team |
| Dissemination of Findings | 1. Publication in peer-reviewed scientific journal  2. Abstract submissions and presentations at scientific conferences (local and international)  3. Dissemination event for key stakeholders (*i.e.,* policy makers and commissioners) | Post-Project | Entire Team |
| HELP Resource | 1. Professionally produced and refined HELP resource | 12 | Entire Team |

HELP = HEart faiLure carer support Programme.

## **Project Timeline**


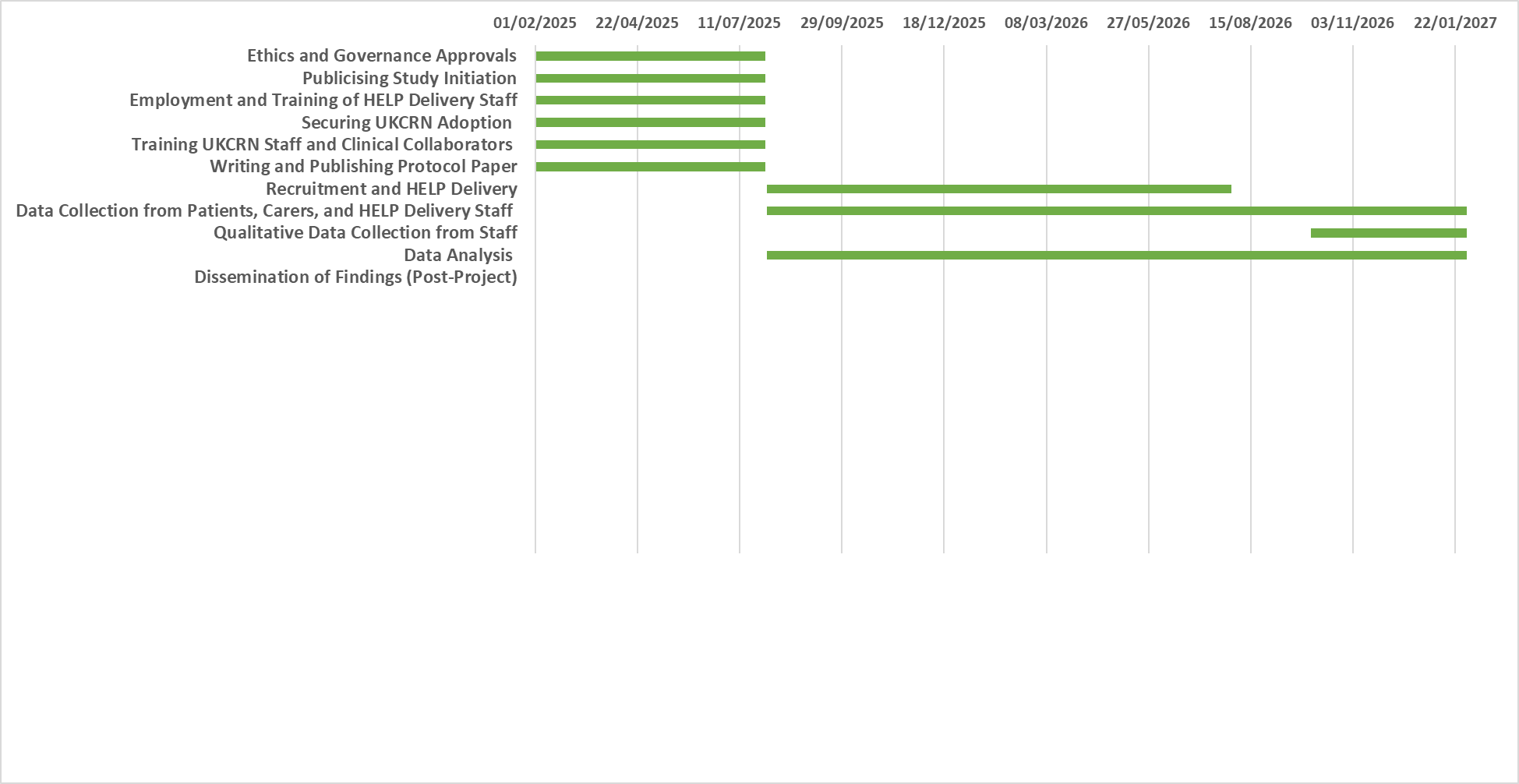


## **Reference List**

1. Lippi G, Sanchis-Gomar F. Global epidemiology and future trends of heart failure. AME Med J. 2020;5:15–15. doi: 10.21037/amj.2020.03.03

2. McDonagh TA, Metra M, Adamo M, Gardner RS, Baumbach A, Böhm M, et al. 2021 ESC Guidelines for the diagnosis and treatment of acute and chronic heart failureDeveloped by the Task Force for the diagnosis and treatment of acute and chronic heart failure of the European Society of Cardiology (ESC) With the special contribution of the Heart Failure Association (HFA) of the ESC. Eur Heart J. 2021;42(36):3599–726. <https://doi.org/10.1093/eurheartj/ehab368>

3. Fitzsimons D, Doherty LC, Murphy M, Dixon L, Donnelly P, McDonald K, et al. Inadequate Communication Exacerbates the Support Needs of Current and Bereaved Caregivers in Advanced Heart Failure and Impedes Shared Decision-making. Journal of Cardiovascular Nursing. 2019;34(1):11–9. doi: 10.1097/JCN.0000000000000516

4. McIlfatrick S, Doherty LC, Murphy M, Dixon L, Donnelly P, McDonald K, et al. ‘The importance of planning for the future’: Burden and unmet needs of caregivers’ in advanced heart failure: A mixed methods study. Palliat Med. 2018;32(4):881–90. <https://doi.org/10.1177/0269216317743958>

5. Wingham J, Frost J, Britten N, Jolly K, Greaves C, Abraham C, et al. Needs of caregivers in heart failure management: A qualitative study. Chronic Illn. 2015;11(4):304–19. <https://doi.org/10.1177/1742395315574765>

6. Grant JS, Graven LJ. Heart failure caregivers’ support services: Implications for palliative care. Prog Palliat Care. 2020;28(5):318–25. <https://doi.org/10.1080/09699260.2020.1716146>

7. Doherty LC, Fitzsimons D, McIlfatrick SJ. Carers’ needs in advanced heart failure: A systematic narrative review. European Journal of Cardiovascular Nursing. 2016;15(4):203–12. <https://doi.org/10.1177/1474515115585237>

8. Cassidy L, Hill L, Fitzsimons D, McGaughey J. The impact of psychoeducational interventions on the outcomes of caregivers of patients with heart failure: A systematic review and meta-analysis. Int J Nurs Stud. 2021;114:103806. <https://doi.org/10.1016/j.ijnurstu.2020.103806>

9. O’Cathain A, Croot L, Duncan E, Rousseau N, Sworn K, Turner KM, et al. Guidance on how to develop complex interventions to improve health and healthcare. BMJ Open. 2019;9(8):e029954. <https://doi.org/10.1136/bmjopen-2019-029954>

10. Skivington K, Matthews L, Simpson SA, Craig P, Baird J, Blazeby JM, et al. Framework for the development and evaluation of complex interventions: gap analysis, workshop and consultation-informed update. Health Technol Assess. 2021;25(57):1–132. <https://doi.org/10.3310/hta25570>

11. Damschroder LJ, Aron DC, Keith RE, Kirsh SR, Alexander JA, Lowery JC. Fostering implementation of health services research findings into practice: a consolidated framework for advancing implementation science. Implementation Science. 2009;4(1):50. doi: 10.1186/1748-5908-4-50

12. Cassidy L. The HEart faiLure carer support Programme: A feasibility study of the HELP intervention for carers. 2022. <https://pure.qub.ac.uk/en/studentTheses/the-heart-failure-carer-support-programme>

13. Schroeder D, Luig T, Finch T.L, et al. Understanding implementation context and social processes through integrating Normalization Process Theory (NPT) and the Consolidated Framework for Implementation Research (CFIR). Implement Sci Commun. 2022; 3(13). <https://doi.org/10.1186/s43058-022-00264-8>

14. King DK, Shoup JA, Raebel MA, Anderson CB, Wagner NM, Ritzwoller DP, et al. Planning for Implementation Success Using RE-AIM and CFIR Frameworks: A Qualitative Study. Front Public Health. 2020;8. <https://doi.org/10.3389/fpubh.2020.00059>

15. Pinnock H, Barwick M, Carpenter CR, Eldridge S, Grandes G, Griffiths CJ, et al. Standards for Reporting Implementation Studies (StaRI) Statement. BMJ. 2017;i6795. doi: 10.1136/bmj.i6795

16. Greenhalgh T, Robert G, Macfarlane F, Bate P, Kyriakidou O. Diffusion of Innovations in Service Organizations: Systematic Review and Recommendations. Milbank Q. 2004;82(4):581–629. doi: 10.1111/j.0887-378X.2004.00325.x

17. Thompson G, Caughers G, Bradley J, Donnelly P, Mooney M, Fitzsimons D. The feasibility of delivering cardiac brief intervention to patients following ST-elevation myocardial infarction: Protocol for a pilot randomised controlled trial. PLoS One. 2024;19(7):e0306406. <https://doi.org/10.1371/journal.pone.0306406>

18. Lazarus R, Folkman S. Stress, appraisal, and coping. Springer Publishing Company; 1984.

19. Hudson P. A conceptual model and key variables for guiding supportive interventions for family caregivers of people receiving palliative care. Palliat Support Care. 2003;1(4):353–65. doi: 10.1017/s1478951503030426

20. Cane J, O’Connor D, Michie S. Validation of the theoretical domains framework for use in behaviour change and implementation research. Implement Sci. 2012;7:37.

21. Borrelli B. The assessment, monitoring, and enhancement of treatment fidelity in public health clinical trials. J Public Health Dent. 2011;71(s1). <https://doi.org/10.1111/j.1752-7325.2011.00233.x>

22. Price B. Laddered questions and qualitative data research interviews. J Adv Nurs. 2002;37(3):273–81. <https://doi.org/10.1046/j.1365-2648.2002.02086.x>

23. Hennink M, Kaiser BN. Sample sizes for saturation in qualitative research: A systematic review of empirical tests. Soc Sci Med. 2022;292:114523. <https://doi.org/10.1016/j.socscimed.2021.114523>

24. Campbell S, Greenwood M, Prior S, Shearer T, Walkem K, Young S, et al. Purposive sampling: complex or simple? Research case examples. Journal of Research in Nursing. 2020;25(8):652–61. <https://doi.org/10.1177/1744987120927206>

25. Rajabi Mashhadi MT, Mashhadinejad H, Ebrahimzadeh MH, Golhasani-Keshtan F, Ebrahimi H, Zarei Z. The Zarit Caregiver Burden Interview Short Form (ZBI-12) in spouses of Veterans with Chronic Spinal Cord Injury, Validity and Reliability of the Persian Version. Arch Bone Jt Surg. 2015;3(1):56–63. doi: 10.22038/abjs.2015.3795

26. Archbold PG, Stewart BJ, Greenlick MR, Harvath T. Mutuality and preparedness as predictors of caregiver role strain. Res Nurs Health. 1990;13(6):375–84. doi: 10.1002/nur.4770130605

27. Ware JE, Kosinski M, Keller SD. A 12-Item Short-Form Health Survey. Med Care. 1996;34(3):220–33. doi: 10.1097/00005650-199603000-00003

28. Zigmond AS, Snaith RP. The Hospital Anxiety and Depression Scale. Acta Psychiatr Scand. 1983;67(6):361–70. <https://doi.org/10.1111/j.1600-0447.1983.tb09716.x>

29. Cohen S, Kamarck T, Mermelstein R. A Global Measure of Perceived Stress. J Health Soc Behav. 1983;24(4):385. <https://doi.org/10.2307/2136404>

30. Zimet GD, Dahlem NW, Zimet SG, Farley GK. The Multidimensional Scale of Perceived Social Support. J Pers Assess. 1988;52(1):30–41. <https://doi.org/10.1207/s15327752jpa5201_2>

31. Rector TS, Cohn JN. Assessment of patient outcome with the Minnesota Living with Heart Failure questionnaire: reliability and validity during a randomized, double-blind, placebo-controlled trial of pimobendane. Am Heart J. 1992;124(4):1017-25. doi: 10.1016/0002-8703(92)90986-6

32. Devlin NJ, Brooks R. EQ-5D and the EuroQol Group: Past, Present and Future. Appl Health Econ Health Policy. 2017;15(2):127–37. doi: 10.1007/s40258-017-0310-5

33. Ward DJ, Furber C, Tierney S, Swallow V. Using Framework Analysis in nursing research: a worked example. J Adv Nurs. 2013;69(11):2423–31. <https://doi.org/10.1111/jan.12127>

34. Farmer T, Robinson K, Elliott SJ, Eyles J. Developing and Implementing a Triangulation Protocol for Qualitative Health Research. Qual Health Res. 2006;16(3):377–94. <https://doi.org/10.1177/1049732305285708>

35. Jones K, Burns A. Unit Costs of Health and Social Care 2021. 2021. <https://kar.kent.ac.uk/92342/25/Unit%20Costs%20Report%202021%20-%20Final%20version%20for%20publication%20%28AMENDED2%29.pdf>.

36. Remawi BN, Gadoud A, Preston N. The experiences of patients with advanced heart failure, family carers, and health professionals with palliative care services: a secondary reflexive thematic analysis of longitudinal interview data. BMC Palliat Care. 2023; 22(115). <https://doi.org/10.1186/s12904-023-01241-1>

37. Moore GF, Audrey S, Barker M, Bond L, Bonell C, Hardeman W, et al. Process evaluation of complex interventions: Medical Research Council guidance. BMJ. 2015;350. doi: https://doi.org/10.1136/bmj.h1258

38. Hawe P, Shiell A, Riley T. Complex interventions: how “out of control” can a randomised controlled trial be? BMJ. 2004;328(7455):1561–3. doi: https://doi.org/10.1136/bmj.328.7455.1561

39. Loi SM, Tropea J, Gaffy E, Panayiotou A, Capon H, Chiang J, et al. START-online: acceptability and feasibility of an online intervention for carers of people living with dementia. Pilot Feasibility Stud. 2022;8(1):41. <https://doi.org/10.1186/s40814-022-00999-0>

40. NHS England. Greener NHS. 2024. <https://www.england.nhs.uk/greenernhs/>

41. Department for Environment, Gove M. 25 Year Environmental Plan. 2023. <https://www.gov.uk/government/publications/25-year-environment-plan>

42. O’Neill B, McAuley D, Willis A, Ji C, Jenkins K, Connolly B, et al. Remote multicomponent rehabilitation compared to standard care for survivors of critical illness after hospital discharge: a randomised controlled assessor-blind clinical and cost-effectiveness trial with internal pilot (iRehab). 2024. <https://www.fundingawards.nihr.ac.uk/award/NIHR132871>

43. Fitzsimons D, Strachan PH. Overcoming the challenges of conducting research with people who have advanced heart failure and palliative care needs. European Journal of Cardiovascular Nursing. 2012;11(2):248–54. <https://doi.org/10.1016/j.ejcnurse.2010.12.002>
