# Supplementary material for: Evaluating the implementation and impact of the HEart faiLure carer support Programme (HELP) in the United Kingdom: A study protocol for a multi-centre, mixed-method, implementation study
Source: PLoS One. 2026 Apr 17;21(4):e0347037. doi: 10.1371/journal.pone.0347037 (PMC13089873; doi:10.1371/journal.pone.0347037)
Supplement: S7 Text — (PDF) [file pone.0347037.s007.pdf]

West Midlands - South Birmingham Research Ethics Committee
2 Redman Place
Stratford
London
E20 1JQ

Telephone: 0207 104 8029

Please note: This is the favourable opinion of the REC only and does not allow you to start your study at NHS sites in England until you receive HRA Approval

07 August 2025

Professor Donna Fitzsimons
Professor of Nursing
Queen's University Belfast
School of Nursing and Midwifery
Medical Biology Centre
97 Lisburn Road, Belfast, Northern Ireland
BT9 7BL

Dear Professor Fitzsimons

**Study title:**	**Implementation Testing of the HEart faiLure carer support Programme (HELP) in the United Kingdom.**
**REC reference:**	**25/WM/0110**
**Protocol number:**	**N/A**
**IRAS project ID:**	**353770**

Thank you for your letter received on 07 August 2025, responding to the Research Ethics Committee's (REC) request for further information on the above research and submitting revised documentation.

Confirmation of ethical opinion

On behalf of the Committee, I am pleased to confirm a favourable ethical opinion for the above research on the basis described in the application form, protocol and supporting documentation

as revised, subject to the conditions specified below.

Good practice principles and responsibilities

The UK Policy Framework for Health and Social Care Research sets out principles of good practice in the management and conduct of health and social care research. It also outlines the responsibilities of individuals and organisations, including those related to the four elements of research transparency:

1. registering research studies
2. reporting results
3. informing participants
4. sharing study data and tissue

Conditions of the favourable opinion

The REC favourable opinion is subject to the following conditions being met prior to the start of the study.

Confirmation of Capacity and Capability (in England, Northern Ireland and Wales) or NHS management permission (in Scotland) should be sought from all NHS organisations involved in the study in accordance with NHS research governance arrangements. Each NHS organisation must confirm through the signing of agreements and/or other documents that it has given permission for the research to proceed (except where explicitly specified otherwise).

Guidance on applying for HRA and HCRW Approval (England and Wales)/ NHS permission for research is available in the Integrated Research Application System.

For non-NHS sites, site management permission should be obtained in accordance with the procedures of the relevant host organisation.

Sponsors are not required to notify the Committee of management permissions from host organisations

Registration of Clinical Trials

All research should be registered in a publicly accessible database and we expect all researchers, research sponsors and others to meet this fundamental best practice standard.

It is a condition of the REC favourable opinion that **all clinical trials are registered** on a public registry before the first participant is recruited and no later than six weeks after. For this purpose, ‘clinical trials’ are defined as:

- clinical trial of an investigational medicinal product
- clinical investigation or other study of a medical device
- combined trial of an investigational medicinal product and an investigational medical device
- other clinical trial to study a novel intervention or randomised clinical trial to compare interventions in clinical practice.

A 'public registry' means any registry on the WHO list of primary registries or the ICMJE list of registries provided the registry facilitates public access to information about the UK trial.

Failure to register a clinical trial is a breach of these approval conditions, unless a deferral has been agreed by the HRA (for more information on registration and requesting a deferral see: Research registration and research project identifiers).

Where a deferral is agreed we expect the sponsor to publish a minimal record on a publicly accessible registry. When the deferral period ends, the sponsor should publish the full record on the same registry, to fulfil the condition of the REC favourable opinion.

If you have not already included registration details in your IRAS application form you should notify the REC of the registration details as soon as possible.

Where the study is registered on ClinicalTrials.gov, please inform deferrals@hra.nhs.uk and the Research Ethics Committee (REC) which issued the final ethical opinion so that our records can be updated.

Publication of Your Research Summary

We will publish your research summary for the above study on the research summaries section of our website, together with your contact details, no earlier than three months from the date of this favourable opinion letter. Where a deferral is agreed, a minimum research summary will still be published in the research summaries database. At the end of the deferral period, we will publish the full research summary.

Should you wish to provide a substitute contact point, make a request to defer, or require further information, please visit: Research summaries - Health Research Authority (hra.nhs.uk)

It is the responsibility of the sponsor to ensure that all the conditions are complied with before the start of the study or its initiation at a particular site (as applicable).

After ethical review: Reporting requirements

The attached document “After ethical review – guidance for researchers” gives detailed guidance on reporting requirements for studies with a favourable opinion, including:

- Notifying substantial amendments
- Adding new sites and investigators
- Notification of serious breaches of the protocol
- Progress and safety reports
- Notifying the end of the study, including early termination of the study
- Final report
- Reporting results

The latest guidance on these topics can be found at Managing your approval - Health Research Authority (hra.nhs.uk)

Ethical review of research sites

NHS/HSC sites

The favourable opinion applies to all NHS/HSC sites taking part in the study, subject to confirmation of Capacity and Capability (in England, Northern Ireland and Wales) or management permission (in Scotland) being obtained from the NHS/HSC R&D office prior to the start of the study (see "Conditions of the favourable opinion" below).

Non-NHS/HSC sites

I am pleased to confirm that the favourable opinion applies to any non-NHS/HSC sites listed in the application, subject to site management permission being obtained prior to the start of the study at the site.

Approved documents

The final list of documents reviewed and approved by the Committee is as follows:

Document	*Version*	*Date*
Covering letter on headed paper [Covering Letter]	1	10 December 2024
Evidence of Sponsor insurance or indemnity (non NHS Sponsors only) [Evidence of Indemnity]		01 August 2024
Evidence of Sponsor insurance or indemnity (non NHS Sponsors only) [Evidence of Indemnity]		07 August 2025
Interview schedules or topic guides for participants [Interview Topic Guide_Carers]	1	10 December 2024
Interview schedules or topic guides for participants [Interview Topic Guide_Patients]	1	10 December 2024
Interview schedules or topic guides for participants [Interview Topic Guide_Healthcare Professionals]	1	10 December 2024
Interview schedules or topic guides for participants [Interview Topic Guide_HELP Delivery Staff]	1	10 December 2024
IRAS Application Form [IRAS_Form_14052025]		14 May 2025
Letter from funder [Letter From Funder]	1	11 September 2024
Letter from sponsor [Letter From Sponsor]	N/A	12 May 2025
Letters of invitation to participant [Invitation to Participate in Interview_Patients]	1	10 December 2024
Letters of invitation to participant [Invitation to Participate_Patients]	2	20 June 2025
Letters of invitation to participant [Invitation to Participate_Carers]	2	20 June 2025
Letters of invitation to participant [Invitation to Participate in Interview_Healthcare Professionals]	2	20 June 2025
Letters of invitation to participant [Invitation to Participate in Interview_Carers]	2	20 June 2025
Non-validated questionnaire [Screening Questionnaire_Patients]	1	10 December 2024
Non-validated questionnaire [Intervention Acceptability Questionnaire_Carers]	1	10 December 2024
Non-validated questionnaire [Intervention Acceptability Questionnaire_HELP Facilitators]	1	10 December 2024
Non-validated questionnaire [Screening Questionnaire_Carers]	1	10 December 2024

Non-validated questionnaire [Demographic Questionnaire_Patients]	1	10 December 2024
Non-validated questionnaire [Demographic Questionnaire_Healthcare Professionals and HELP Delivery Staff]	1	10 December 2024
Non-validated questionnaire [Weekly HELP Impact Questionnaire_Carers]	2	20 June 2025
Non-validated questionnaire [Weekly HELP Impact Questionnaire_Patients]	2	20 June 2025
Non-validated questionnaire [HELP Long-Term Adherence Questionnaire_Carers]	2	20 June 2025
Non-validated questionnaire [HELP Long-Term Adherence Questionnaire_Patients]	2	20 June 2025
Non-validated questionnaire [Demographic Questionnaire_Carers]	2	20 June 2025
Other [Template Delegation Log]	1	10 December 2024
Other [HELP Support Group schedule]	1	10 December 2024
Other [Implementation Log]	1	10 December 2024
Other [HELP Educational Booklet]	1	12 May 2025
Other [Instructions to Join ZOOM sessions]	1	10 December 2024
Other [Attendance Record_Support Group Sessions]	1	10 December 2024
Other [Intervention Fidelity Checklist]	1	10 December 2024
Other [Facilitator Log]	1	10 December 2024
Other [Case Report Form_Carers]	1	10 December 2024
Other [Case Report Form_Patients]	1	10 December 2024
Other [Distress Protocol]	1	10 December 2024
Other [Flow Diagram of Potential Participant Identification]	2	20 June 2025
Other [Factsheet for Potential Participant Identification]	2	20 June 2025
Other [Patient Permission to be Contacted Form]	2	20 June 2025
Other [Data Extraction Form_Patient Clinical Data]	2	20 June 2025
Other [Distress Protocol]	2	20 June 2025
Other [Response to Provisional Opinion]	1	07 August 2025
Other [SWGR Responses]	1	07 August 2025
Participant consent form [Verbal Consent Script_Patients]	2	20 June 2025
Participant consent form [Verbal Consent Script_Carers]	2	20 June 2025
Participant consent form [Verbal Consent Script_Carer Interviews]	2	20 June 2025
Participant consent form [Verbal Consent Script_Patient Interviews]	2	20 June 2025
Participant consent form [Verbal Consent Script_Healthcare Professional Interviews]	2	20 June 2025
Participant consent form [Verbal Consent Script_HELP Delivery Staff Interviews]	2	20 June 2025
Participant information sheet (PIS) [Patient Information Pack]	2	20 June 2025
Participant information sheet (PIS) [Carer Information Pack]	2	20 June 2025
Participant information sheet (PIS) [Carer Information Pack_Interviews]	2	20 June 2025
Participant information sheet (PIS) [Patient Information Pack_Interviews]	2	20 June 2025
Participant information sheet (PIS) [Healthcare Professional	2	20 June 2025

Information Pack_Interviews]		
Research protocol or project proposal [Study Protocol]	1	10 December 2024
Research protocol or project proposal [Study Protocol]	2	20 June 2025
Summary CV for Chief Investigator (CI) [CV for CI]	1	15 January 2025
Summary, synopsis or diagram (flowchart) of protocol in non technical language [Study Flow Diagram]	1	10 December 2024
Validated questionnaire [Questionnaire Booklet_Carers]	1	10 December 2024
Validated questionnaire [Questionnaire Booklet_Patients]	1	10 December 2024

Statement of compliance

The Committee is constituted in accordance with the Governance Arrangements for Research Ethics Committees and complies fully with the Standard Operating Procedures for Research Ethics Committees in the UK.

User Feedback

The Health Research Authority is continually striving to provide a high quality service to all applicants and sponsors. You are invited to give your view of the service you have received and the application procedure. If you wish to make your views known please use the feedback form available on the HRA website: [Quality assurance - Health Research Authority (hra.nhs.uk)]

HRA Learning

We are pleased to welcome researchers and research staff to our HRA Learning Events and online learning opportunities– see details at: [Learning - Health Research Authority (hra.nhs.uk)]

**IRAS project ID: 353770**	**Please quote this number on all correspondence**

With the Committee's best wishes for the success of this project.

Yours sincerely
PP

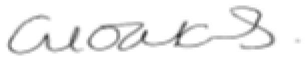

Dr Kathryn Kinmond
Vice Chair

Email: southbirmingham.rec@hra.nhs.uk

Enclosures: "After ethical review – guidance for researchers"

Copy to: Ms Paula Tighe

Lead Nation
approvals@hra.nhs.uk
