# Supplementary material for: Evaluating the implementation and impact of the HEart faiLure carer support Programme (HELP) in the United Kingdom: A study protocol for a multi-centre, mixed-method, implementation study
Source: PLoS One. 2026 Apr 17;21(4):e0347037. doi: 10.1371/journal.pone.0347037 (PMC13089873; doi:10.1371/journal.pone.0347037)
Supplement: S8 Text — (PDF) [file pone.0347037.s008.pdf]

Professor Donna Fitzsimons
Professor of Nursing
Queen's University Belfast
School of Nursing and Midwifery
Medical Biology Centre
97 Lisburn Road, Belfast, Northern Ireland
BT9 7BL

Email: approvals@hra.nhs.uk
HCRW.approvals@wales.nhs.uk

17 November 2025

Dear Professor Fitzsimons

HRA and Health and Care Research Wales (HCRW) Approval Letter

**Study title:**	**Implementation Testing of the HEart faiLure carer support Programme (HELP) in the United Kingdom.**
**IRAS project ID:**	**353770**
**Protocol number:**	**N/A**
**REC reference:**	**25/WM/0110**
**Sponsor**	**Queen's University Belfast**

I am pleased to confirm that **HRA and Health and Care Research Wales (HCRW) Approval** has been given for the above referenced study, on the basis described in the application form, protocol, supporting documentation and any clarifications received. You should not expect to receive anything further relating to this application.

Please now work with participating NHS organisations to confirm capacity and capability, in line with the instructions provided in the "Information to support study set up" section towards the end of this letter.

How should I work with participating NHS/HSC organisations in Northern Ireland and Scotland?

HRA and HCRW Approval does not apply to NHS/HSC organisations within Northern Ireland and Scotland.

If you indicated in your IRAS form that you do have participating organisations in either of these devolved administrations, the final document set and the study wide governance report (including this letter) have been sent to the coordinating centre of each participating nation. The relevant national coordinating function/s will contact you as appropriate.

Please see IRAS Help for information on working with NHS/HSC organisations in Northern Ireland and Scotland.

How should I work with participating non-NHS organisations?
HRA and HCRW Approval does not apply to non-NHS organisations. You should work with your non-NHS organisations to obtain local agreement in accordance with their procedures.

What are my notification responsibilities during the study?

The standard conditions document "*After Ethical Review – guidance for sponsors and investigators*", issued with your REC favourable opinion, gives detailed guidance on reporting expectations for studies, including:

- Registration of research
- Notifying amendments
- Notifying the end of the study

The HRA website also provides guidance on these topics, and is updated in the light of changes in reporting expectations or procedures.

Who should I contact for further information?
Please do not hesitate to contact me for assistance with this application. My contact details are below.

Your IRAS project ID is **353770**. Please quote this on all correspondence.

Yours sincerely,

Joanna Strickland

Approvals Specialist

Email: approvals@hra.nhs.uk

Copy to: *Ms Paula Tighe*

List of Documents

The final document set assessed and approved by HRA and HCRW Approval is listed below.

Document	*Version*	*Date*
Contract/Study Agreement template [mNCA_Clean]	2	07 August 2025
Covering letter on headed paper [Covering Letter]	1	10 December 2024
Evidence of Sponsor insurance or indemnity (non NHS Sponsors only) [Evidence of Indemnity]		07 August 2025
Interview schedules or topic guides for participants [Interview Topic Guide_Carers]	1	10 December 2024
Interview schedules or topic guides for participants [Interview Topic Guide_Patients]	1	10 December 2024
Interview schedules or topic guides for participants [Interview Topic Guide_Healthcare Professionals]	1	10 December 2024
Interview schedules or topic guides for participants [Interview Topic Guide_HELP Delivery Staff]	1	10 December 2024
IRAS Application Form [IRAS_Form_14052025]		14 May 2025
IRAS Application Form XML file [IRAS_Form_14052025]		14 May 2025
Letter from funder [Letter From Funder]	1	11 September 2024
Letter from sponsor [Letter From Sponsor]	N/A	12 May 2025
Letters of invitation to participant [Invitation to Participate_Patients]	2	20 June 2025
Letters of invitation to participant [Invitation to Participate_Carers]	2	20 June 2025
Letters of invitation to participant [Invitation to Participate in Interview_Healthcare Professionals]	2	20 June 2025
Letters of invitation to participant [Invitation to Participate in Interview_Carers]	2	20 June 2025
Letters of invitation to participant [Invitation to Participate in Interview_Patients]	1	10 December 2024
Non-validated questionnaire [Weekly HELP Impact Questionnaire_Carers]	2	20 June 2025
Non-validated questionnaire [Weekly HELP Impact Questionnaire_Patients]	2	20 June 2025
Non-validated questionnaire [HELP Long-Term Adherence Questionnaire_Carers]	2	20 June 2025
Non-validated questionnaire [HELP Long-Term Adherence Questionnaire_Patients]	2	20 June 2025
Non-validated questionnaire [Demographic Questionnaire_Carers]	2	20 June 2025
Non-validated questionnaire [Screening Questionnaire_Patients]	1	10 December 2024
Non-validated questionnaire [Intervention Acceptability Questionnaire_Carers]	1	10 December 2024
Non-validated questionnaire [Intervention Acceptability	1	10 December

Questionnaire_HELP Facilitators]		2024
Non-validated questionnaire [Screening Questionnaire_Carers]	1	10 December 2024
Non-validated questionnaire [Demographic Questionnaire_Patients]	1	10 December 2024
Non-validated questionnaire [Demographic Questionnaire_Healthcare Professionals and HELP Delivery Staff]	1	10 December 2024
Organisation Information Document [Outline OID]	2	07 August 2025
Other [Flow Diagram of Potential Participant Identification]	2	20 June 2025
Other [Factsheet for Potential Participant Identification]	2	20 June 2025
Other [Patient Permission to be Contacted Form]	2	20 June 2025
Other [Data Extraction Form_Patient Clinical Data]	2	20 June 2025
Other [Distress Protocol]	2	20 June 2025
Other [Response to Provisional Opinion]	1	07 August 2025
Other [SWGR Responses]	1	07 August 2025
Other [Template Delegation Log]	1	10 December 2024
Other [HELP Support Group schedule]	1	10 December 2024
Other [Implementation Log]	1	10 December 2024
Other [HELP Educational Booklet]	1	12 May 2025
Other [Instructions to Join ZOOM sessions]	1	10 December 2024
Other [Attendance Record_Support Group Sessions]	1	10 December 2024
Other [Intervention Fidelity Checklist]	1	10 December 2024
Other [Facilitator Log]	1	10 December 2024
Participant consent form [Verbal Consent Script_Patients]	2	20 June 2025
Participant consent form [Verbal Consent Script_Carers]	2	20 June 2025
Participant consent form [Verbal Consent Script_Carer Interviews]	2	20 June 2025
Participant consent form [Verbal Consent Script_Patient Interviews]	2	20 June 2025
Participant consent form [Verbal Consent Script_Healthcare Professional Interviews]	2	20 June 2025
Participant consent form [Verbal Consent Script_HELP Delivery Staff Interviews]	2	20 June 2025
Participant information sheet (PIS) [Patient Information Pack]	2	20 June 2025
Participant information sheet (PIS) [Carer Information Pack]	2	20 June 2025
Participant information sheet (PIS) [Carer Information Pack_Interviews]	2	20 June 2025

Participant information sheet (PIS) [Patient Information Pack_Interviews]	2	20 June 2025
Participant information sheet (PIS) [Healthcare Professional Information Pack_Interviews]	2	20 June 2025
Research protocol or project proposal [Study Protocol]	2	20 June 2025
Schedule of Events or SoECAT [SoE]	2	07 August 2025
Summary CV for Chief Investigator (CI) [CV for CI]	1	15 January 2025
Summary, synopsis or diagram (flowchart) of protocol in non technical language [Study Flow Diagram]	1	10 December 2024
Validated questionnaire [Questionnaire Booklet_Carers]	1	10 December 2024
Validated questionnaire [Questionnaire Booklet_Patients]	1	10 December 2024

IRAS project ID	353770

Information to support study set up

The below provides all parties with information to support the arranging and confirming of capacity and capability with participating NHS organisations in England and Wales. This is intended to be an accurate reflection of the study at the time of issue of this letter.

Types of participating NHS organisation	Expectations related to confirmation of capacity and capability	Agreement to be used	Funding arrangements	Oversight expectations	HR Good Practice Resource Pack expectations
Research activities and procedures as per the protocol and other study documents will take place at participating NHS organisations.	Research activities should not commence at participating NHS organisations in England or Wales prior to their formal confirmation of capacity and capability to deliver the study in accordance with the contracting expectations detailed.	An Organisation Information Document has been submitted but the sponsor is intending to use a separate agreement. The sponsor has supplied the unmodified model non-commercial study agreement v2.3 July 2022 and intends to use this with participating NHS organisations.	Study funding arrangements are detailed in A65 of the IRAS form.	A Principal Investigator should be appointed at participating NHS organisations.	Where an external individual who does not already hold an NHS employment contract will be conducting any of the research activities that will be undertaken at this site type then they would be expected to hold an Honorary Research Contract. External staff holding pre-existing NHS employment contracts should obtain a Letter of Access. These should confirm Occupational Health Clearance. These should confirm enhanced DBS checks and appropriate barred list checks.

Other information to aid study set-up and delivery

This details any other information that may be helpful to sponsors and participating NHS organisations in England and Wales in study set-up.
The applicant has indicated that they intend to apply for inclusion on the NIHR CRN Portfolio. Please note that there was a delay in issuing the HRA Approval due to a delay in the confirmation of the study wide governance report from Northern Ireland to the HRA.
